# Recent gene selection and drug resistance underscore clinical adaptation across *Candida* species

**DOI:** 10.1038/s41564-023-01547-z

**Published:** 2024-01-04

**Authors:** Miquel Àngel Schikora-Tamarit, Toni Gabaldón

**Affiliations:** 1 Barcelona Supercomputing Centre (BSC-CNS), Barcelona, Spain; 2 Institute for Research in Biomedicine (IRB Barcelona), The Barcelona Institute of Science and Technology, Barcelona, Spain; 3 Catalan Institution for Research and Advanced Studies (ICREA), Barcelona, Spain; 4 Centro Investigación Biomédica En Red de Enfermedades Infecciosas, Barcelona, Spain

**Keywords:** Pathogens, Evolutionary genetics, Fungi

## Abstract

Understanding how microbial pathogens adapt to treatments, humans and clinical environments is key to infer mechanisms of virulence, transmission and drug resistance. This may help improve therapies and diagnostics for infections with a poor prognosis, such as those caused by fungal pathogens, including *Candida*. Here we analysed genomic variants across approximately 2,000 isolates from six *Candida* species (*C. glabrata*, *C. auris*, *C. albicans*, *C. tropicalis*, *C. parapsilosis* and *C. orthopsilosis*) and identified genes under recent selection, suggesting a highly complex clinical adaptation. These involve species-specific and convergently affected adaptive mechanisms, such as adhesion. Using convergence-based genome-wide association studies we identified known drivers of drug resistance alongside potentially novel players. Finally, our analyses reveal an important role of structural variants and suggest an unexpected involvement of (para)sexual recombination in the spread of resistance. Our results provide insights on how opportunistic pathogens adapt to human-related environments and unearth candidate genes that deserve future attention.

## Abstract (web summary)

Phylogenetics and convergence-based genome-wide association studies were used to analyse genomic variants across approximately 2,000 isolates from six *Candida* species to reveal how *Candida* fungal pathogens adapt to drugs and clinical environments.

## Main

Fungal infections pose a serious health threat, affecting more than one billion people and causing approximately 1.5 million deaths each year^1,2^. The problem is growing due to insufficient diagnostic and therapeutic options^3,4^, increasing numbers of susceptible patients^1,5^, the expansion of pathogens partly linked to climate change^6,7^ and the alarming rise of antifungal drug resistance^4,8,9^. *Candida* species are a major cause of severe hospital-acquired infections^1^, prompting the classification of some species (*Candida auris*, *Candida albicans*, *Candida glabrata*, *Candida tropicalis* and *Candida parapsilosis*) as critical or high-priority targets by the World Health Organization^2^.

A promising strategy to improve current therapies is to understand the evolutionary mechanisms of adaptation to antifungal drugs as well as to the human host. *Candida* pathogens have highly dynamic genomes (both within species^10–12^ and within patient^13,14^), which probably underlie these adaptive processes^13,15–18^. For example, in vitro evolution studies have pinpointed genome-wide changes underlying drug resistance^19–21^. In addition, analyses of serial clinical isolates^13,14^, genome-wide association studies (GWAS)^22,23^ and population genomics research^11,12,24^ have partially clarified the clinical relevance of resistance mechanisms. Similarly, directed evolution experiments in mice^25–27^, the analysis of paired clinical isolates^13^ and population genomics studies^12,28^ have explored host adaptation mechanisms involving virulence, adhesion or filamentous growth. Furthermore, some studies used ratios between non-synonymous and synonymous variation (such as *π*_N_/*π*_S_) to infer signatures of selection, which are useful to predict genes involved in clinical adaptation where the relevant phenotypes (such as drug susceptibility or cell adhesion within a patient) are not measurable^12,29–31^.

**Fig. 1:**
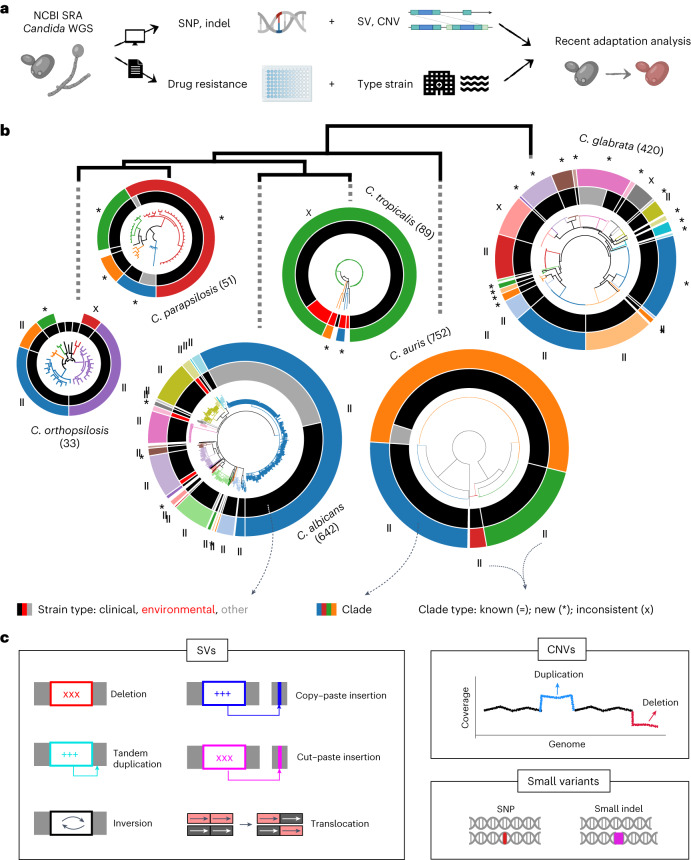
A genome dataset to study recent evolution across major *Candida* species. **a**, Overview of the data-generation process. To study the genome-wide signs of recent selection and drug resistance, we processed available whole-genome sequencing datasets from the National Center for Biotechnology Information Sequence Read Archive (NCBI SRA) for *C. glabrata*, *C. auris*, *C. albicans*, *C. tropicalis*, *C. parapsilosis* and *C. orthopsilosis*. We used these data to identify SNPs, indels, CNVs and SVs in each strain. In addition, we manually curated the associated literature to obtain antifungal drug-susceptibility data and information about the type of strain (that is, clinical or environmental). WGS, whole-genome sequencing. **b**, SNP-based trees for all strains of each species (Methods). The size of each tree is proportional (in logarithmic scale) to the number of strains (indicated in parentheses). The clades inferred here are represented in different colours in the branches and outer strips. Symbols were used to indicate how each clade overlaps with clades defined in other recent population studies (*C. albicans*^28^, *C. auris*^11^, *C. glabrata*^12^, *C. tropicalis*^24^ and *C. orthopsilosis*^36^): =, known (one-to-one match); *, new; and X, inconsistent (it is inconsistent with previous clade definitions; Methods). Supplementary Table 1 includes all the clade definitions as well as the trees in Newick format. The inner strip represents the type of strain, where ‘other’ refers to strains with engineered genomes or strains resulting from directed evolution experiments. In this inner strip, the width of each colour indicates the number of strains of each type in each clade but they are not displayed in the order of the tree. Branches with support < 95 were collapsed. The species tree (top) was obtained using OrthoFinder. **c**, Variant types identified in this study. Structural variants are complex rearrangements identified with a breakpoint-detection algorithm, whereas CNVs are variants generating large duplications and deletions inferred from changes in coverage (Methods).

However, our understanding of how *Candida* species adapt in a clinical context is limited due to many reasons. First, most clinical studies include small sample sizes and/or lack rigorous statistical testing of the associations between genotypes and adaptive changes. Second, most studies involve only *C. albicans*, leaving open questions in other species^2^. Third, despite the importance of structural variants (SVs; such as deletions, duplications, inversions and/or translocations; Fig. 1)^32–34^, their contribution to clinically relevant adaptation remains largely unexplored. Fourth, similarities in adaptation mechanisms across species remain elusive because most studies focus on only one species and use different methods. This is key to understanding the epidemiology of these pathogens as well as enabling personalized treatments and prevention strategies. Fifth, many exploratory clinical studies focus only on known adaptive mechanisms (that is, known drug-resistance genes, as discussed previously^23^), which means that there may be unexplored factors. Finally, current studies of selection consider all variants within a gene, which may reflect ancient adaptation unrelated to the clinics. It may be important to only analyse recently emerged variants, as they are more likely to reflect clinically relevant selective pressures (as proposed in ref. ^35^).

To address these gaps, we used approximately 2,000 available genomes from major *Candida* species to investigate two open questions in clinical adaptation. First, we used phylogenetics and *π*_N_/*π*_S_-inspired tools to infer the genes with signatures of recent and potentially clinically relevant selection in *C. glabrata*, *C. auris*, *C. albicans*, *C. tropicalis*, *C. parapsilosis* and *C. orthopsilosis*. Second, we used convergence-based GWAS to infer the genomic drivers of resistance to echinocandins, polyenes and azoles in *C. glabrata*, *C. auris* and *C. albicans*. In both cases we measured the contribution of various variant types, including SVs. Our analyses revealed both expected and novel adaptive mechanisms, including those convergently acting in several species.

## Results and discussion

### Public sequences allow the study of recent evolution in *Candida*

**Extended Data Fig. 1:**
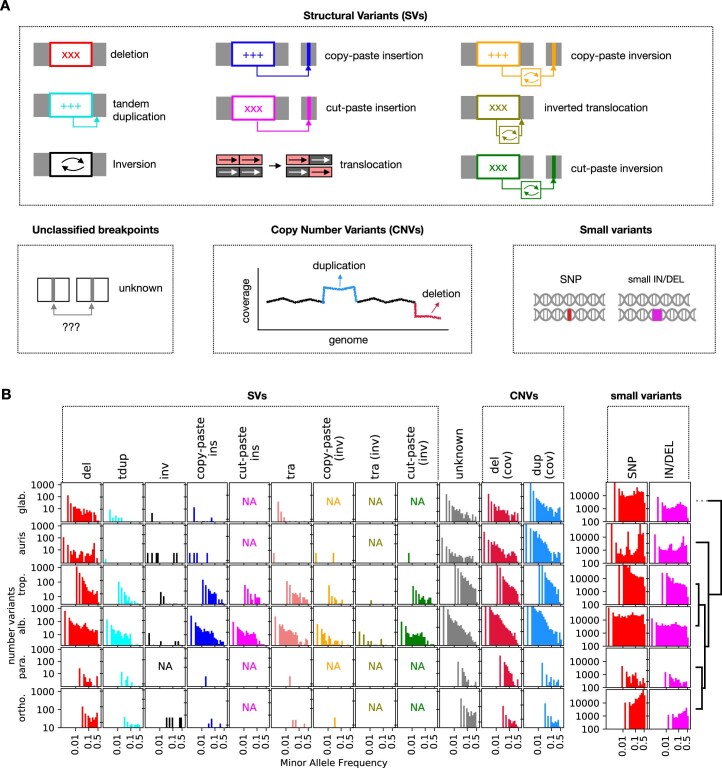
Our dataset includes different variant types. **a**, Representation of the types of variants identified in this work. SVs are complex variants where we could find the precise underlying rearrangements and breakpoint positions. Unclassified breakpoints are also a type of SV where we do not know the exact type of underlying rearrangement, either because it is an unknown type of SV or because we missed other breakpoints that could explain the SV. Conversely, CNVs are variants generating large (>600 bp) duplications and deletions (inferred from changes in coverage) with unknown underlying rearrangements. **b**, Distribution of Minor Allele Frequencies across all variant types and species. ‘NA’ indicates that a given variant type could not be found in that species.

To identify genes under recent selection in *Candida* pathogens, we retrieved all publicly available short-read whole-genome sequencing data for pure isolates (that is, clinical and environmental) of six major species and identified four variant types: single-nucleotide polymorphisms (SNPs), small insertions and deletions (indels), SVs and copy-number variants (CNVs; Methods, Fig. 1 and Extended Data Fig. 1). We enriched genomic information with strain metadata from the literature, including isolation source and antifungal drug susceptibility, where available (Fig. 1b and Supplementary Table 1). This dataset, comprising 1,987 high-quality samples (available at https://candidamine.org), is unprecedented in terms of the types of variants and number of strains considered^11,12,24,28,36^.

To provide a phylogenetic framework to our analysis, we inferred a strain tree (Fig. 1b and Supplementary Table 1) and used a systematic approach to identify genetically divergent monophyletic clades in each species (Methods and Supplementary Fig. 1a). A comparison with previously defined clades (Methods) revealed an overall consistency, underscoring the validity of our clade-definition approach, but also showed that our dataset encompasses a higher intraspecific diversity. In summary, we generated a dataset with unprecedented power to study the signs of selection and drug-resistance mechanisms in major *Candida* pathogens.

### Structural variants underlie intraspecific variation

**Fig. 2:**
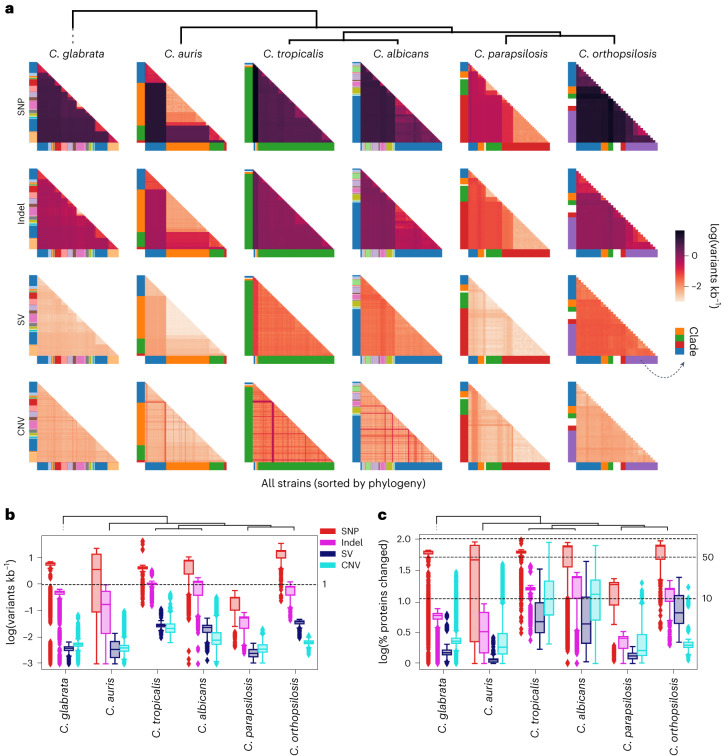
All variant types contribute significantly to intraspecific diversity. **a**, Overview of the genetic distance (variants kb^−1^) patterns across all species generated by each variant type. Each row and column represents a strain ordered as in the strain tree and coloured by clade (see Fig. 1b); each cell corresponds to the genetic distance (log-transformed) between all pairs of strains. We added a pseudocount of 0.001 variants kb^−1^ for the logarithmic calculations. **b**, The same as in **a** but as a boxplot. Each cell in **a** corresponds to one point in the distributions shown here. Thus, there are $\frac{n!}{2!(n-2)!}$ points for each box in a given species, where *n* represents the number of strains (see Fig. 1b). For instance, for a species with five strains we would have $\frac{5!}{2!(5-2)!}=10$ comparisons. These data correspond to biological replicates, as each point corresponds to a pairwise comparison between two strains. **c**, Distribution of the predicted percentage of proteins that are altered by the different variant types across all pairs of strains. Each point of the distribution corresponds to a pair of strains, shown in a boxplot as in **b**. We added a pseudocount of 1% of genes affected for the logarithmic calculations. **b**,**c**, Boxplots: the box represents the interquartile range of the distribution, from the first to the third quartile, with the line representing the median. The whiskers extend to points that lie within 1.5× the interquartile range of the first and third quartiles, and values outside this range are shown as independent points.

To determine the relevance of considering different variant types in subsequent analyses, we quantified their relative contribution to genetic diversity. Such comparative analysis across *Candida* species is lacking, as most previous studies have focused on SNPs and used specific methodologies. For each variant type, we measured the genetic distance (variants kb^−1^) between all pairs of isolates within a given species. We found that most species span high levels of diversity so that some distant conspecific strains have a genetic distance of about 10 SNPs kb^−1^ (1% divergence) or higher (Fig. 2a,b). In some species (that is, *C. orthopsilosis*^36^ and *C. albicans*^10^), this could be attributed to their hybrid nature. For non-hybrid species (*C. glabrata* and *C. auris*), this indicates that their diversification predates human colonization, which must have occurred in parallel in divergent clades for each species. *C. parapsilosis* is an exception to this trend, pointing to a more recent origin of this lineage.

**Extended Data Fig. 2:**
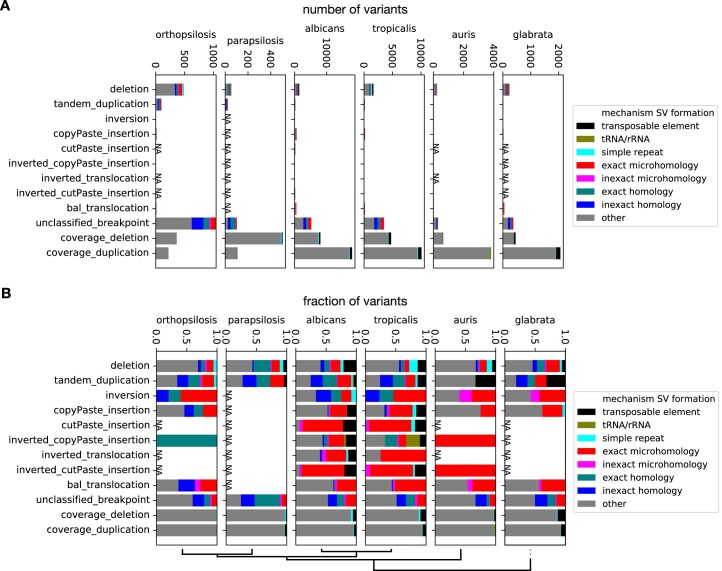
Proportion of SVs and CNVs attributable to different mechanisms of formation. **a**, Absolute number of variants, and **b**, the fraction of variants relative to each species and type of variant. Variants potentially biased by simple repeats were discarded, including CNVs largely overlapping simple repeats and SVs where the breakpoints are around such repeats. This explains why there are some variants classified as ‘simple repeats’ (e.g. deletions that involve a region with simple repeats but where the breakpoints do not overlap them). Variants classified as ‘other’ could not be assigned to any of the other categories. Note that the categories ‘exact microhomology’, ‘inexact microhomology’, ‘exact homology’ and ‘inexact homology’ refer to variants likely generated via homologous recombination. In addition, we interpret that the variants not attributable to any of these categories (‘other’) are likely generated by Non-Homologous End Joining repair pathways. See Methods for more details.

Regarding non-SNP variants, we found that the SV and indel distances correlate to SNP distances (Fig. 2a), which suggests that they were accurately called. Conversely, the CNV and SNP distances were not always correlated (Fig. 2a), which could be attributed to inaccurate definitions of CNV boundaries, probably complicating distance metrics. As expected, SNPs were quantitatively the most common variant type, followed by indels—one order of magnitude less prominent—and then SVs and CNVs at much lower frequencies (Fig. 2b). Despite their lower abundance, SVs and CNVs can affect a significant fraction of protein-coding genes (Fig. 2c), highlighting their relevance. We investigated the mechanisms underlying the formation of SVs and CNVs, and found that most variants are unrelated to repetitive elements or rearrangements derived from homologous recombination (Extended Data Fig. 2 and Methods). This suggests that non-homologous-end-joining DNA repair pathways^37,38^ could be the main driver of SVs and CNVs in *Candida* species, consistent with such repair often resulting in rearrangements^39^. In summary, we found that all variant types are quantitatively important and therefore should not be overlooked in subsequent analyses.

### Signatures of recent selection reveal adaptation mechanisms

To infer the signatures of recent clinically relevant selection, we took advantage of the predominance of clinical strains in our collection. We reasoned that recently acquired variants in clinical isolates may be enriched in those acquired in a clinical context and could therefore inform on selective pressures related to adaptation to human-related environments. The standard approach of calculating *π*_N_/*π*_S_ ratios^12,31,40,41^ is not suitable for our aim for the following reasons. First, we focus on recently acquired variants and *π*_N_/*π*_S_ considers all mutations in a gene, thereby also detecting ancient selection. Second, considering only recent variants poses a statistical challenge to reliably calculate *π*_N_/*π*_S_, given that many genes have few recent variants and thus a *π*_S_ of zero. Third, *π*_N_/*π*_S_ cannot be applied to indels, SVs and CNVs, which we deem important.

**Fig. 3:**
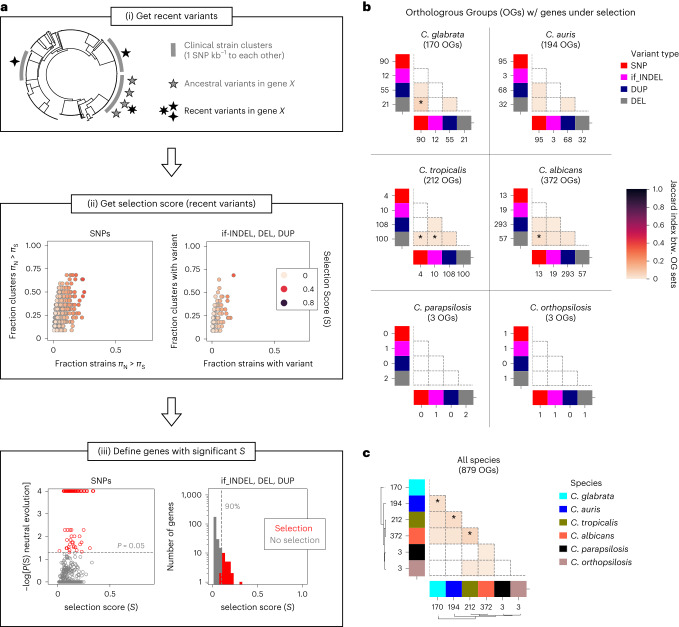
Genome-wide signatures of recent selection in clinical isolates of *Candida* species. **a**, Schematic representation of our pipeline for measuring recent selection for each gene by different variant types, using *C. glabrata* as an example. (i) We first defined recent variants as those that were acquired during the diversification of monophyletic clusters of close clinical strains (where all strains have ≤1 SNP kb^−1^ to each other). An example for gene *X* that has three variants, including some that were recently acquired, is shown. The grey stripes represent the relevant strain clusters for this gene. (ii) We then calculated the selection score (*S*), proposed here, which measures whether a gene (each point) has an excess of recurrent, recent functionally relevant variants (non-synonymous SNPs, in-frame indels (if_INDEL), gene duplications (DUP) or gene truncations (DEL)). For SNPs (left), *S* takes into account which strains have a typical hallmark of positive selection (*π*_N_ > *π*_S_). Thus, we defined *S* as the harmonic mean between the fraction of strains with ${\pi }_{\mathrm{N}} > {\pi }_{\mathrm{S}}$ (*x* axis) and the fraction of clusters with at least one strain that has ${\pi }_{\mathrm{N}} > {\pi }_{\mathrm{S}}$ (*y* axis). In the scatter plots we show these values for *C. glabrata* genes. For the other variant types (if_INDEL, DEL and DUP; right) we defined *S* as the harmonic mean between the fraction of strains with a variant in that gene (*x* axis) and the fraction of clusters with at least one strain that has a variant (*y* axis). *S* measures the ‘excess of recurrent variants’ in these variant types. The example shows the results of DEL variants in *C. glabrata*. (iii) Finally, we defined ‘genes under selection’ as those that had a significantly high *S* value. For SNPs (left), we defined genes under selection as those that had a low empirical probability of observing *S* under a neutral model of evolution (false-discovery rate (FDR)-corrected probability *P*(*S*) < 0.05; Methods). The scatterplot shows, for each *C. glabrata* gene, the *S* and −log_10_[FDR-corrected *P*(*S*)] values with significant genes under selection in red. For other variant types (right), we defined genes under selection as those that had an *S* value above the 90th percentile of all genes (red). The list of genes and OGs under selection are in Supplementary Table 2. In addition, Extended Data Figs. 3 and 4 show these distributions for all species and types of variants. **b**, Distribution of the number of gene families (Orthologous Groups, OGs) with genes under selection by different variant types across species. The numbers of such OGs are provided. The heatmaps show the overlap between OGs with genes under selection by different variant types, measured as the Jaccard distance. To infer the significance of having a given number *n* of overlapping OGs across genes under selection by different variant types, we calculated the empiric probability (*P*) of having *n* or more overlapping OGs when taking random genes from each set of compared genes (Methods). For example, there are 25 genes under selection by DELs (from 21 OGs) and 92 genes under selection by SNPs (from 90 OGs) in *C. glabrata* (top left). There are six OGs with genes under selection by both SNPs and DELs, and the probability of having ≥6 overlapping OGs when taking 25 and 92 random genes is 0.0001. Thus, the *P* values come from an empirical one-sided statistical approach. **c**, Distribution of the numbers of OGs with genes under selection (by any variant type) across species. The heatmap shows the overlaps between such OGs as in **b**. *P < 0.05.

**Extended Data Fig. 3:**
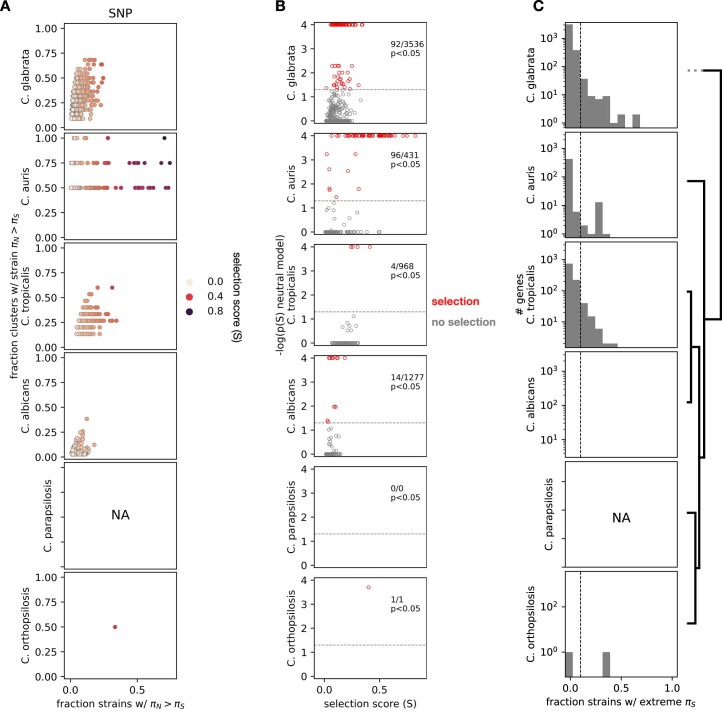
Distribution of selection scores by SNPs across *Candida* species. **a**, Distribution of the selection score *S* and definition of genes with significantly high *S*, shown as in Fig. 3a, for all species and SNPs. Note that the *p(S)* represents the FDR-corrected empirical probability of having an *S* higher or equal to the observed one under a model of neutral evolution (see Methods), which represents a one-sided test. In **b**, the text inset shows the number of genes that are considered to be ‘under selection’, out of all the genes for which we could measure *S*. We only measured *S* values for genes with enough variability to make calculations, which explains why some distributions have few or zero (plots with ‘NA’) genes. We only considered genes with ${\pi }_{N} > {\pi }_{S}$ in ≥2 clusters and ≥3 strains, and we discarded genes where the null model may not be reasonable (see **c**). Note that Supplementary Table 2 includes all these measurements. **c**, Distribution of samples with extreme *π*_*S*_ according to the neutral evolution model. To validate this neutral model, used to define p(*S*) in **b**, we reasoned that the real, observed π_S_ should fall within the simulated, empirical distribution of neutral π_S_ generated by the model. This panel shows the distribution across genes of the fraction of strains with extreme *π*_S_ according to the neutral model (see Methods). We only considered genes with a fraction < 0.1 (dashed line) for all analyses, including the data from **a** and **b**.

**Extended Data Fig. 4:**
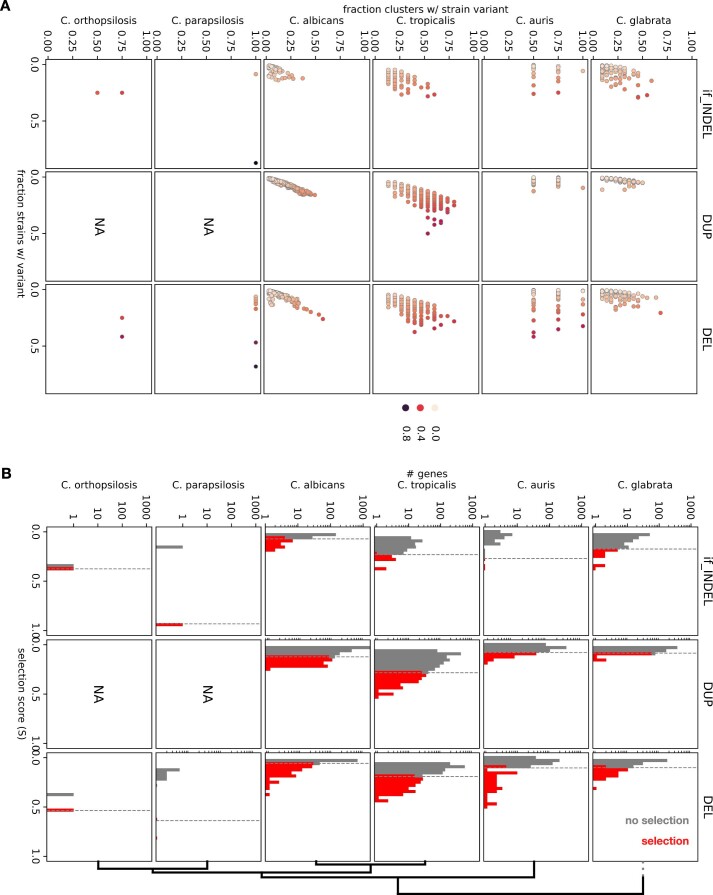
Distribution of selection scores by non-SNP variants across *Candida* species. **a**, Distribution of the selection score *S* and definition of genes with significantly high *S*, shown as in Fig. 3a for all species and types of variants. In **b**, the text inset shows the number of genes that are considered to be ‘under selection’, out of all the genes for which we could measure *S*. We only measured *S* values for genes with enough variability to make calculations, which explains why some distributions have few or zero (plots with ‘NA’) genes. We only considered genes with recent variants in ≥2 clusters and ≥3 strains. Note that Supplementary Table 2 includes all these measurements.

To overcome these drawbacks, we developed a *π*_N_/*π*_S_-inspired method that detects genes with an excess of recent functionally relevant variants (non-synonymous SNPs, in-frame indels, gene duplications or truncations; Methods, Fig. 3a and Extended Data Figs. 3,4). Duplications could be SVs or CNVs, and deletions could be nonsense SNPs, frameshifting indels, SVs or CNVs. Note that an excess of deletions in a gene could reflect either positive selection acting on deletions or recent relaxation of purifying selection. To focus on recent variants, we identified monophyletic clusters comprising only clinical strains with high genetic relatedness (Supplementary Fig. 1) and only considered variants inferred to have appeared within the cluster. These clusters probably represent clonally propagating lineages that evolved in human-associated environments (as they are closely related and recurrently isolated from patients), and therefore recent mutations may reflect selective pressures related to adaptation to the host, hospital environments or antifungal drugs. We used these variants to define genes under recent selection as those with an excess of recurrent functionally relevant variants.

We detected several recently selected genes using our approach, belonging to 879/7,499 orthologous groups (OGs, a proxy for gene families) (Fig. 3b and Supplementary Table 2). The low numbers in *C. orthopsilosis* and *C. parapsilosis* probably reflect reduced statistical power due to few strains and low intraspecific diversity, respectively. Thus, further sequencing efforts will be needed to fully understand the signs of selection in these species. Most OGs are affected by a single variant type, with few exceptions that suggest complex evolutionary interactions (sometimes antagonistic) among the OG members (Fig. 3b and Supplementary Results). We found several expected genes related to virulence and drug resistance, providing support for the validity of our approach (Fig. 3b and Supplementary Table 2). Some examples include *ALS* genes in *C. albicans* (implicated in adhesion and biofilm formation^42^); *TAC1b*, *ERG11* and *MRR1* in *C. auris* (related to azole resistance^11,21,43,44^); *PDR1* in *C. glabrata* (implicated in azole resistance^19,45^); *EPA* genes in *C. glabrata* (related to adhesion^32,46^); a drug exporter in *C. orthopsilosis* (gene *CORT_0G00240*) or filamentous growth proteins in *C. tropicalis* (genes *CTRG_00655* and *CTRG_03085*). In addition, significant overlaps between these genes and those with recurrent mutations across clonal within-patient serial isolates were observed, providing support for the idea that these genes are often involved in clinical adaptation (Supplementary Results). Furthermore, there were signs of selection on all variant types in most species, suggesting that considering SVs and CNVs is relevant. This gene catalogue constitutes a valuable resource to validate the clinical relevance of evolutionary mechanisms inferred from future non-clinical studies (that is, in vitro evolution^19,21^, virulence in animal models^27^ or high-throughput genotype–phenotype screenings^47,48^).

To understand the similarities in selective processes across species, we screened for OGs with a gene affected by selection in multiple taxa. Only 68/879 such OGs were identified, suggesting that each species has unique signatures of selection (Fig. 3c). Although this could be partly attributed to different sampling criteria and statistical power across taxa, it is consistent with generally different infection mechanisms in each species, which is also reflected in mostly non-overlapping transcriptional profiles on host interactions^49,50^. However, in many instances the number of overlapping OGs was higher than expected by chance (*P* < 0.05; Methods, Fig. 3c and Supplementary Table 2), pointing to convergent adaptive mechanisms in *Candida* pathogens. Relevant example genes within these OGs include *ALS* genes from *C. albicans* and *C. auris*, *OPT2* and *OPT3* (transporters related to pseudohyphal growth and fluconazole presence) in *C. albicans* and *C. tropicalis*, *MRR1a* in *C. auris* and *C. tropicalis* (related to drug resistance), *FLO8* and *MSS11* (related to pseudohyphal growth) in *C. glabrata* and *C. auris*, *MDS3* (virulence factor) in *C. albicans* and *C. auris*, *CST6* (associated with azole resistance^22^) in *C. glabrata* and *C. auris*, and *WOR4* (related to phenotype switching) in *C. albicans* and *C. auris*.

**Fig. 4:**
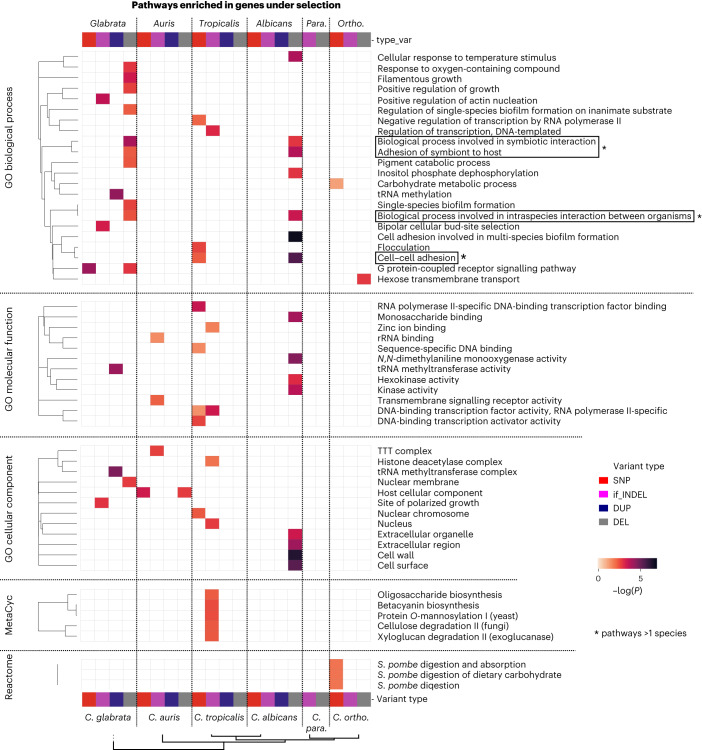
Species-specific and conserved functions are enriched among genes under recent selection. Heatmap representing the GO terms, MetaCyc and Reactome annotations that were enriched in genes under recent selection in different species by different variant types. The enrichment *P* values were calculated using a one-sided Fisher’s exact test, followed by FDR-based correction. Only pathways with an FDR-corrected *P* < 0.05 were considered as significant and shown here; this *P* value is shown in the colour map. The GO terms are clustered by Lin’s semantic similarity for ease of comparison. In addition, we ran a REVIGO-like redundancy-reduction algorithm to only keep representative terms for this plot (Methods). Conversely, the Reactome and MetaCyc pathways are clustered according to the Jaccard distance between the OGs affected in different sets of genes. Pathways enriched in genes under selection in >1 taxa are indicated with asterisks. *C. para*., *C. parapsilosis*; *C. ortho*., *C. orthopsilosis.* Supplementary Table 2 contains all of the related enrichments.

We performed enrichment analyses on functional annotations and identified 1,074 domains, 151 gene ontology (GO) terms, five MetaCyc and three Reactome pathways that were enriched across all gene sets (Fig. 4, Supplementary Fig. 2 and Supplementary Table 2), including hyphal growth, biofilm formation, transcriptional regulation, response to temperature, cell adhesion, carbohydrate metabolism, cell wall and membrane regions (Fig. 4). Most enriched functional groups are unique to a single species (991/1,074 domains, 143/151 GO terms, and all MetaCyc and Reactome pathways), suggesting that each species has unique signatures of recent selection also at the pathway and domain level (Fig. 4). These species-specific enrichments reflect the distinct adaptive paths affecting each of these *Candida* pathogens (discussed in Supplementary Results). However, there are several convergently affected pathways and domains, which may reflect conserved adaptive mechanisms (Fig. 4 and Supplementary Fig. 2). Relevant examples include a zinc-dependent transcription factor domain in *C. tropicalis*, *C. albicans* and *C. auris*; disordered regions in *C. tropicalis*, *C. albicans* and *C. glabrata*, and hyphally regulated cell wall proteins in *C. tropicalis*, *C. albicans* and *C. auris* (Supplementary Fig. 2). Several GO terms related to adhesion (‘biological process involved in symbiotic interaction’, ‘adhesion of symbiont to host’ and ‘cell–cell adhesion’) were also enriched in genes with selected deletions from *C. tropicalis*, *C. albicans* and *C. glabrata*, suggesting recurrent rewiring of these functions (Fig. 4). Further research is needed to associate these functions with possible adaptive advantages. For instance, disordered proteins can generate new traits in yeast^51^ and the deletion of adhesion genes could modulate host attachment, biofilm formation or immune evasion^52–55^, therefore improving survival. In summary, our results suggest hundreds of gene families (about 10% of all families) and pathways under recent selective pressure, often in a single species. This may be explained by the natural niche of these pathogens being massively different to the human host. In addition, we found convergently selected families and pathways that may be at the core of recent adaptation and constitute interesting therapeutic targets. Future experiments should validate these results and pinpoint the most important drivers of recent adaptation.

### Convergence GWAS to study antifungal resistance

**Fig. 5:**
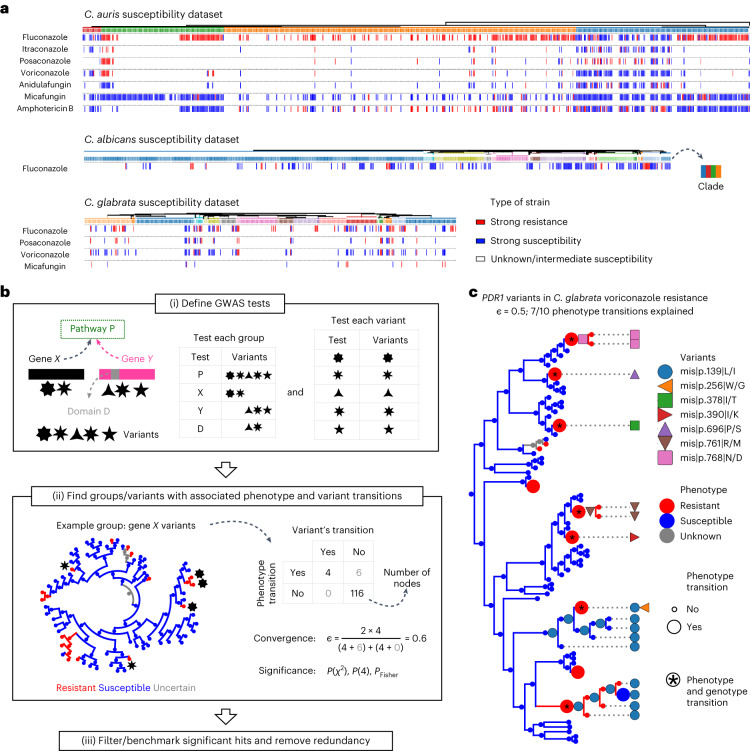
Genome-wide genotype–phenotype associations underlying resistance towards antifungal drugs. **a**, Drug-susceptibility data were available for a fraction of our strains (Supplementary Table 1), which motivated us to perform a convergence-based GWAS study to understand the genomic determinants of this phenotype. These plots show the distribution of the available drug-susceptibility data across the tree of each species for which we performed such a GWAS. We only considered strains with either strong susceptibility or strong resistance; we discarded those with intermediate susceptibility or unavailable data. We only performed a GWAS on these datasets because we could find ≥5 transitions from strong susceptibility to strong resistance or vice versa in the evolutionary history of these strains. The clades are colour coded (as in Fig. 1b), showing how each dataset covers the diversity of each species. Supplementary Table 1 includes all these data. **b,** Schematic view of the GWAS pipeline. (i) First, we defined the GWAS tests to be performed, which included one test for each variant and one test for different groups of collapsed variants (to take into account that different variants may drive resistance by altering the same gene, domain or pathway). (ii) We then tried to find groups (or single variants) where transitions in the variants were significantly associated with phenotype transitions. An example group, ‘gene *X*’, which has two variants (black stars) associated with changes in voriconazole resistance in *C. glabrata* is shown. In the tree the colours (equivalent to **a**) represent the resistance state of each node of (inferred with ASR). To measure the strength and significance of the association, we generated a two-by-two table with the number of nodes that have a transition in the resistance phenotype and/or a transition in any of the variants of the group (‘gene *X*’ in this case). In this example there are four nodes with both a transition in the phenotypes and in some variants. The strength of the association was approximated with the convergence statistic *ε*, and the significance was inferred with various *P* values for each group, such as $P({\chi}^{2})$, $P(4)$ or ${P}_{\mathrm{Fisher}}$. For example, *P*(4) is the empiric probability of having ≥4 nodes with both variant and phenotype transitions by chance (Methods). (iii) Finally, we used information on known drug-resistance genes to choose a filtering strategy for each dataset (such as which *P* values to consider), resulting in the final set of high-confidence GWAS associations (hits). In addition, we kept only non-redundant hits (see Methods and Supplementary Table 3). **c**, Visual representation of an example high-confidence GWAS hit—that is, variants in the gene *PDR1* that are correlated to voriconazole resistance in *C. glabrata*. The tree represents the strains with available voriconazole-susceptibility information. At each node, the resistance phenotype (resistant, susceptible or unknown) and presence of different variants (all missense mutations) are indicated. In ancestral nodes, these phenotypes or variants were inferred with ASR. To illustrate relevant transitions, the size of the sphere indicates whether the node has a phenotype transition (so that the phenotype in the node is different from the parent phenotype); phenotype-transition nodes that also have a transition in the variants (genotype transition) are indicated. For clarity, only *PDR1* variants that are correlated to resistance in some nodes are shown. In this case there are ten phenotype transitions, seven of which are also correlated to a transition in *PDR1*.

Drug susceptibility is a measurable phenotype that has been determined for a sizeable fraction of the strains used in our study (Supplementary Table 1 and Fig. 5a), which motivated us to find genomic changes underlying the drug-resistance phenotype in clinical isolates. For this, we performed a convergence-based GWAS, which uses ancestral state reconstruction (ASR) to find variant changes that are significantly associated with transitions in drug-resistance phenotypes in their reconstructed evolutionary histories^56,57^. Given the peculiarities of our dataset, we developed a custom pipeline, inspired by the hogwash synchronous algorithm^58^ (Methods and Fig. 5b). In addition, we tested the association between groups of collapsed variants and the phenotype to take into account that different variants may drive drug resistance by altering the same feature (a gene or a pathway). To focus on key associations, we only analysed species–drug combinations with at least five sharp transitions (from high susceptibility to high resistance or vice versa; Methods and Supplementary Fig. 3). This resulted in 12 species–drug datasets including seven compounds from all main classes (azoles, echinocandins and polyenes) and covering most clades of *C. albicans*, *C. glabrata* and *C. auris* (Supplementary Table 1 and Figs. 1b, 5a). To ensure high-confidence hits, we used a conservative approach that minimized the false positives expected from such multiple testing and chose the GWAS algorithm parameters and filtering criteria based on previous expectations of resistance genes (Methods and Supplementary Fig. 4). To remove redundancy, we kept the strongest and most-specific association among overlapping high-confidence variants, genes, domains and pathways (Methods and Supplementary Table 3). As an example of a significant association, we found that small variants affecting *PDR1* (drug-efflux regulator^45^) are correlated with voriconazole resistance in *C. glabrata* (Fig. 5c and Supplementary Table 3). In Supplementary Results we discuss results that do not meet this stringent selection but that we deem interesting.

**Fig. 6:**
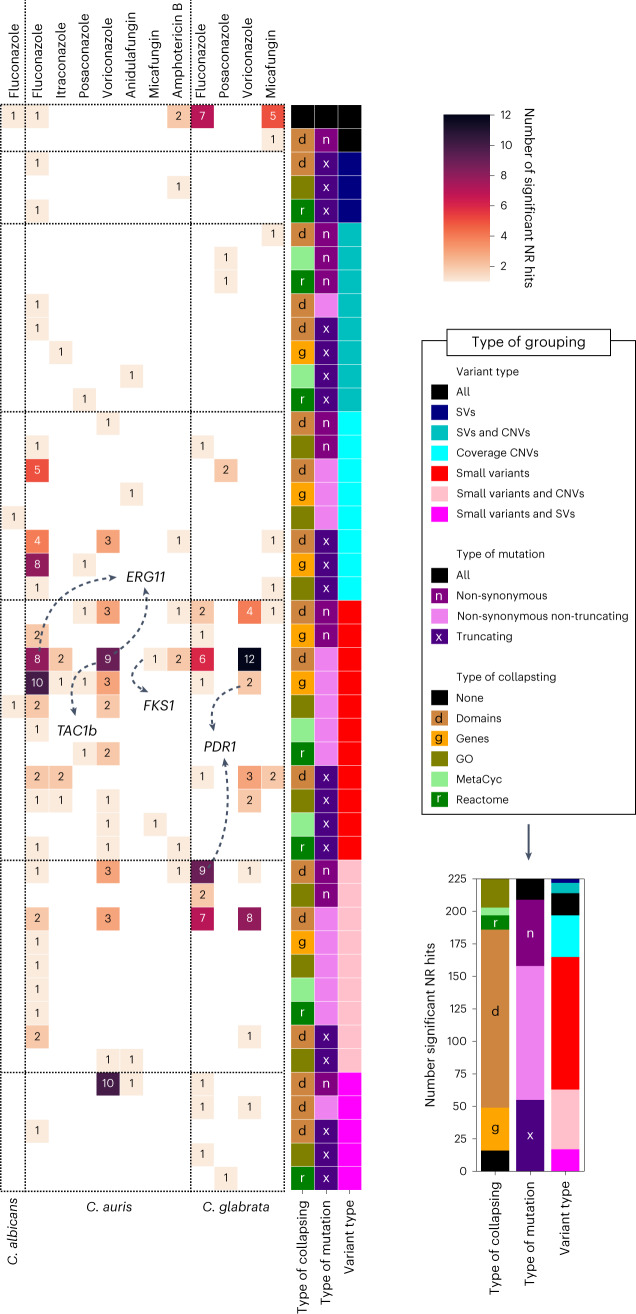
Hundreds of GWAS hits underlie known and potentially novel mechanisms of drug resistance. The heatmap (left) shows the number of high-confidence non-redundant (NR) GWAS hits (or groups) obtained for each dataset (columns) when using different variant grouping strategies (rows). To consider different ways of grouping variants, we performed one ‘grouped’ GWAS for different combinations of the variant type (SVs, CNVs, small variants or any combination thereof), mutation type (non-synonymous, non-synonymous non-truncating or truncating) and collapsing level (domains, genes or pathways (GO, Reactome or MetaCyc)). For example, in one of these GWAS we tested the genotype–phenotype association for each gene (type of collapsing, genes), considering truncating (type of mutation, truncating mutations) small variants and SVs (variant type, small variants and SVs). We thus ran a total of 113 GWAS analyses for each species and drug—one for the single variants (variant type, all variants; type of collapsing, none) and 112 for different combinations of collapsing modes. Each row in the heatmap corresponds to one of these GWAS analyses, restricted to those that yield some high-confidence hits. These grouping strategies yielded redundant results (e.g. a significant variant may drive a significant association in the genes affected by that variant) so that we only kept (and show here) the strongest most-specific association among sets of redundant hits. For example, if we had a gene that is significant when considering either small variants (with *ε* = 0.3) or small variants and SVs (with *ε* = 0.4), we would keep the hit that considers small variants and SVs, as it has the highest *ε*. Similarly, if there was a significant gene (with *ε* = 0.3) and a significant variant altering that gene (with *ε* = 0.3), we would keep the variant as it is more specific. This redundancy reduction ensures that the numbers of hits by different collapsing strategies are informative (that is, hits involving SVs around a gene will only appear here if they yield stronger associations than the hits that only consider small variants in the same gene). The small inset plot (right) summarizes the number of unique hits (for instance, if a gene is found in two datasets it will only count as one hit here) obtained when considering different grouping strategies, which provides information on the most important ones. In addition, the arrows point to hits involving known drug-resistance genes.

**Extended Data Fig. 5:**
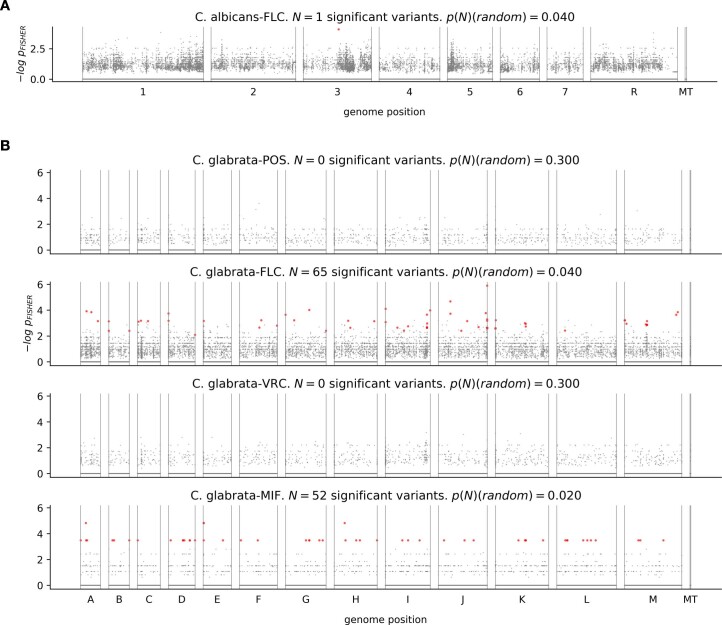
Individual variants are associated with drug resistance in some GWAS datasets of *C. albicans* and *C. glabrata*. **a**,**b**, Manhattan plots showing the Fisher p value (one-sided) of the genotype–phenotype association for each variant (including SNPs, INDELs, CNVs and SVs) along the genomes of *C. albicans* (**a**) and *C. glabrata* (**b**). The x axis represents the genomic position of each variant, with chromosomes separated by vertical lines (where ‘MT’ is the mitochondrial chromosome). Each subplot represents one species / drug for which we performed GWAS. The drugs analysed are fluconazole (FLC), posaconazole (POS), voriconazole (VRC), and micafungin (MIF). Red points indicate variants that passed all the GWAS high-confidence filters (see Methods and Supplementary Fig. 4b). Note that, to maximize the computational efficiency we only tested associations for a subset of relevant variants: those with ${n}_{Gt,Pt}\ge 2$, ${n}_{Gnt,Pnt}\ge 1$ and an odds ratio (of the genotype-vs-phenotype transition table) ≥ 1 (see Methods). For all the other variants we set a *p* = 1, which explains why i) there are many dots with -*log* p=0 and ii) the variant density varies across datasets. In addition, the title of each subplot shows the number of such significant variants (*N*), and *p(N)* represents the empirical probability of observing *N* or more variants under a null model of no association (see Methods). This *p(N)* is relevant because it reflects how well our filtering strategy addresses the multiple testing burden. For instance, we consider that a *p(N)* < 0.05 suggests that this burden is minimal (see Methods). Note that we use the fisher p value because it is appropriate for such a visualization, although it was not always used for the definition of significant hits.

**Extended Data Fig. 6:**
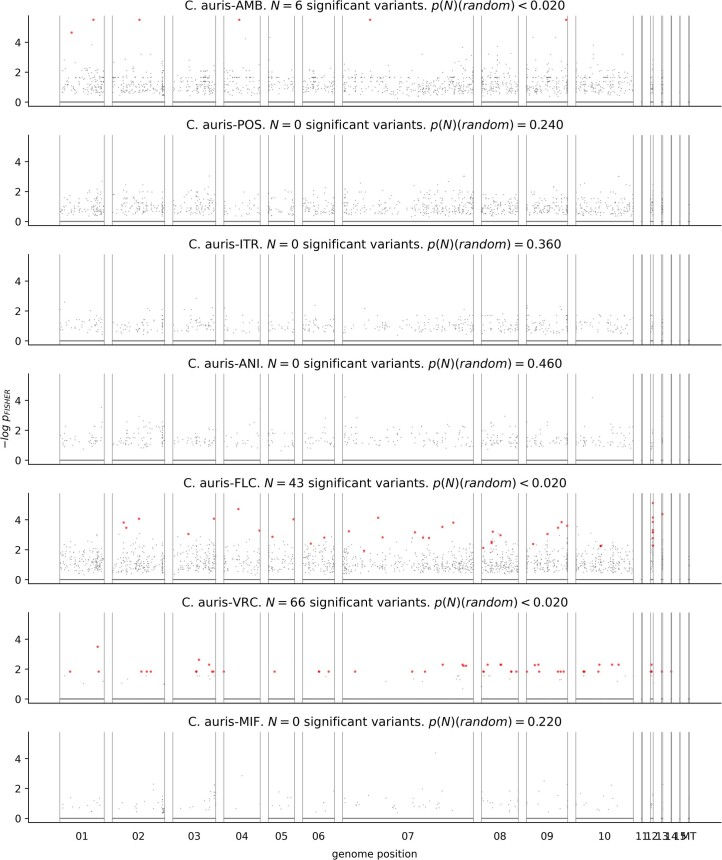
Individual variants are associated with drug resistance in some GWAS datasets of *C. auris*. Equivalent to Extended Data Fig. 5, but for *C.auris* datasets. The drugs analysed are fluconazole (FLC), itraconazole (ITR), posaconazole (POS), voriconazole (VRC), anidulafungin (ANI), micafungin (MIF) and amphotericin B (AMB).

**Extended Data Fig. 7:**
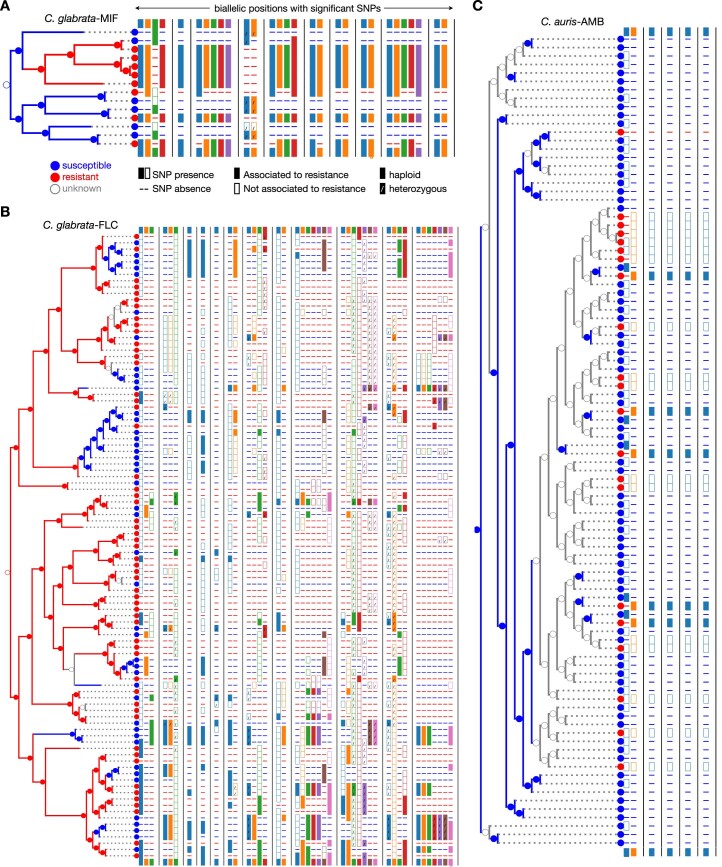
Variants associated with drug resistance are linked to each other. **a**–**c**, Presence/absence pattern of all SNPs significantly associated with resistance (red points in Extended Data Figs. 5, 6) across strains for three example GWAS datasets. These figures show how sets of linked SNPs segregate together with resistance transitions, which was essential to understand the role of recombination in the spread of resistance (see Results and Discussion). Each column represents a genomic position (only biallelic positions are shown) with one of these SNPs, and the grey vertical lines separate variants from different chromosomes. If a strain has a filled box for a given position/variant it means that the variant transition is correlated to a resistance transition in that strain or one of the ancestors. If a strain has an empty box it means that it has the variant, but it is not related to a resistance transition in that strain. The box colours of each column are to ease visualization of where each SNP is in each strain, and they do not reflect any distinct information. The ‘/’ indicates SNPs that are heterozygous and found in duplicated regions. The circles of each node represent resistance (red), susceptibility (blue) or uncertain susceptibility (grey), according to ancestral state reconstruction. The nodes with a support <70 have non-filled circles. In **c** we only show a clade of the tree with the significant SNPs.

**Extended Data Fig. 8:**
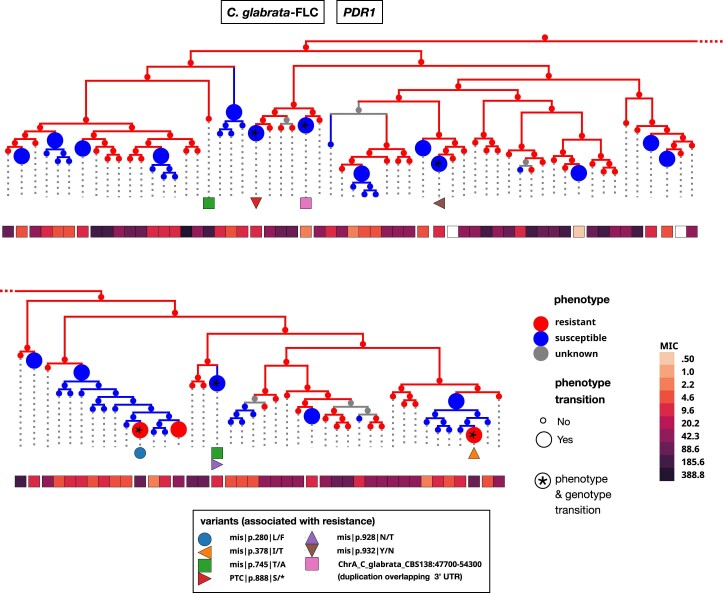
Genotype-phenotype correlation in *PDR1* for the *C.* glabrata–fluconazole GWAS. Representation of *PDR1* variants (those associated with resistance) and susceptibility measurements for the *C. glabrata/*fluconazole (FLC) dataset, shown as in Fig. 5c. The strain tree is split across two boxes to ease visualization. This plot exemplifies the complexity of GWAS signals for these phenotypes, as some *PDR1* variants are associated to a gain of resistance (the missense substitutions mis|p.280|L/F and mis|p.378|I/T), while others are correlated to its loss (the premature termination codon PTC|p.888|S/*, the duplication affecting the 3’ UTR and substitutions mis|p.928|N/T and mis|p.932|Y/N). The colour scale shows the fluconazole MIC for each of these strains. Check the legend of Fig. 5c for further clarification about the other elements in this plot.

Unexpectedly, in some cases the Manhattan plots showing variant–phenotype correlations suggested the existence of linked variants—that is, variants distributed across the genome jointly segregating with the phenotype (Extended Data Figs. 5,6,7 and Supplementary Results). Such a distribution may be explained by recent inter-strain recombination partly underlying the emergence of drug resistance. This is consistent with previous studies suggesting sexual (or parasexual) cycles in these species^12,28,59^ and points to a possible role of (para)sexual recombination in the spread of antifungal resistance. A possible role of recombination makes the detection of causal variants slightly more difficult, as they may be linked to passenger variants unrelated to the phenotype. We therefore focused on protein-altering variants, which are more likely to underlie changes in drug resistance^19,60,61^. When considering all types of groupings, 227 non-redundant significant associations (hits) affecting 130 OGs and 38 pathways across all 12 datasets were identified, with variations across datasets probably reflecting differences in sample size (Supplementary Table 3 and Fig. 6). Close examination of these hits underscored the importance of considering SVs/CNVs and domain/pathway grouping of variants (Supplementary Results and Extended Data Fig. 8).

In summary, our multispecies genotype–phenotype association study revealed genome-wide determinants of drug resistance to all major drug classes. Beyond our analysis, this is a valuable resource to validate that the resistance mechanisms found in future studies are meaningful in clinical isolates, as we illustrate for a recent in vitro evolution study^19^ (Supplementary Results).

### GWAS analysis suggests drivers of drug resistance

**Extended Data Fig. 9:**
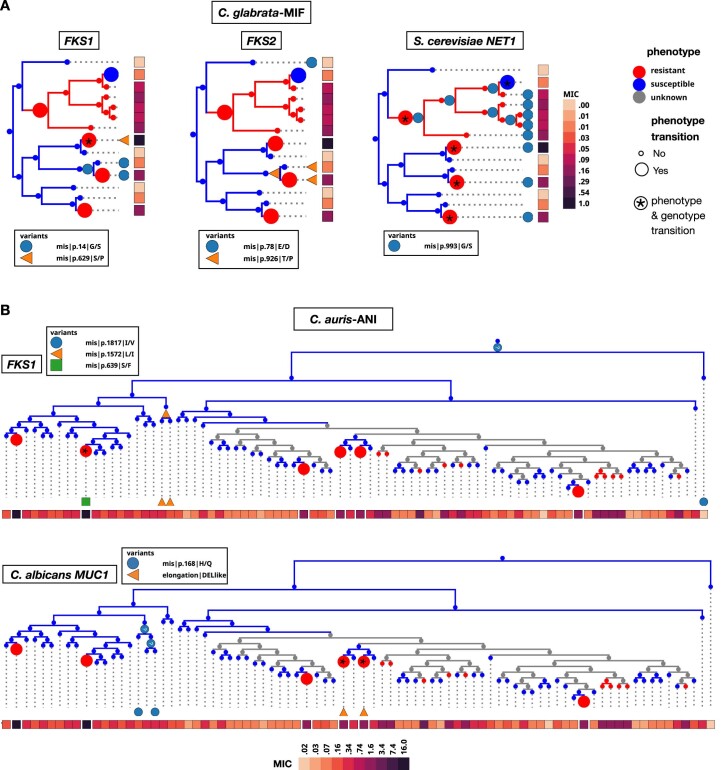
Genotype-phenotype correlation for echinocandin GWAS. **a**, Representation of relevant variants and resistance phenotypes for the *C. glabrata/*micafungin (MIF) dataset, shown as in Extended Data Fig. 8. The left and middle plots show *FKS1/FKS2* variants, which are not associated with resistance. The right plot refers to the top hit in this dataset: a G993S variant in *NET1*. **b**, The same as in **a**, but for the *C. auris/*anidulafungin (ANI) dataset. The upper plot shows *FKS1* variants, and the lower plot refers to the top hit in this dataset: small variants and an SV (rearrangement in the transcript that does not break the gene) in *B9J08_003526* (ortholog of *C. albicans’ MUC1*).

To validate our GWAS strategy and gain insights into known mechanisms of antifungal drug resistance, we checked the GWAS results for expected driver genes (Fig. 6, Extended Data Figs. 8,9, Supplementary Table 3 and Supplementary Results). Our analysis confirmed that *ERG11* (target of azoles^62^) is associated with fluconazole resistance in *C. albicans* as well as fluconazole and voriconazole resistance in *C. auris*, *TAC1b* (drug-efflux regulator^60^) underlies pan-azole resistance in *C. auris*, *FKS* (echinocandin target^63^) mutations are probable drivers of strong pan-echinocandin resistance in *C. auris* and *C. glabrata*, and *PDR1* underlies pan-azole resistance in *C. glabrata*. Conversely, *ERG11* may be unrelated to resistance towards long-tailed azoles in *C. auris*, confirming earlier observations from in vitro studies^64–66^ and showing that resistance mechanisms are not equivalent for all azoles (Supplementary Results).

Beyond these ‘known genes’ our results hint to other players. To focus on the most-relevant potentially conserved mechanisms, we considered OGs associated with resistance in more than one drug–species combination (Supplementary Table 3). These included *PDR1*, *ERG11* and 13 other OGs, which are often (12/13 OGs) related to ‘core’ resistance mechanisms towards multiple drugs of the same species. We identified six such OGs in *C. glabrata* that were related to various azoles and micafungin resistance—that is, four adhesin family members (*CAGL0J01727g*, *PWP4*/*AWP13*, *AWP4*/*AWP9* and *EPA19*/*EPA11*), the orthologue of *Saccharomyces cerevisiae NET1* (putative chromatin-silencing ribosomal RNA regulator) and *CAGL0K07502g* (a protein with unknown function). The link between adhesins and resistance could be explained by their role in biofilm formation, a known resistance mechanism^67,68^. In addition, the role of *NET1* is consistent with studies linking chromatin silencing with azole resistance in *C. glabrata*^69^ as well as with the observation that its deletion in *S. cerevisiae* increases sensitivity to some compounds^70,71^. Similarly, we found six ‘core’ OGs in *C. auris*: *B9J08_005550* (with RNA-binding activity) related to fluconazole and voriconazole resistance, *B9J08_004248* and *B9J08_004896* (putative RNA-dependent DNA polymerases) related to amphotericin B and multiple azole resistance, *B9J08_004249* and *B9J08_005494* (putative zinc-binding transcription factors) associated with amphotericin B and fluconazole resistance, and the orthologue of *S. cerevisiae MRPS35* (mitochondrial ribosomal protein) related to itraconazole and voriconazole resistance. These results suggest that different aspects of gene regulation (transcription and RNA life-cycle regulation) are key for multidrug resistance in *C. auris*. In addition, the role of *MRPS35* is consistent with the observations that its deletion decreases resistance to some compounds in *S. cerevisiae*^71^ and that mitochondrial regulation is linked to drug efflux in *C. albicans*^72^.

**Extended Data Fig. 10:**
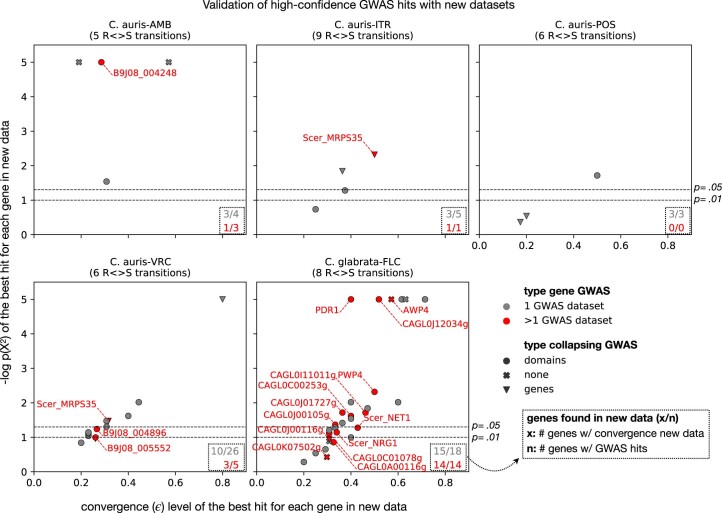
Confirmation of high-confidence GWAS hits with an independent dataset. To validate the GWAS results we tested whether we could find similar signatures of genotype–phenotype convergence in datasets published between June 2020 and June 2023, which were not used in the GWAS analysis. We could find sufficient data to do this (with at least five sharp resistance transitions) for five species–drug combinations (each of the panels of this figure). The number in parenthesis shows the number of phenotype transitions. The drugs analysed are fluconazole (FLC), itraconazole (ITR), posaconazole (POS), voriconazole (VRC) and amphotericin B (AMB). For each gene with a high-confidence hit in the GWAS (Supplementary Table 3), we ran a genotype–phenotype association testing for an array of parameters and types of groupings equivalent to those used in the GWAS analysis (see Methods). The small insets in the lower-right corner of the plot show the number of genes with high-confidence GWAS hits (n) that also had some degree of convergence (at least two nodes in the tree with both genotype and phenotype transitions, see Methods) in the new independent datasets (x). Each of the points represents the −log[*p(X*^*2*^*)*] and convergence level (*ε*) for the best hit related to a given gene in the new dataset, which indicates the significance and strength of the genotype–phenotype associations. This *p(X*^*2*^*)* is an empirical p value coming from a one-sided test, with no correction (see Methods). The colours represent whether the gene belongs to an OG that was found in one (grey) or more (red) GWAS datasets. The symbols represent whether the GWAS hits came from domain, gene or no grouping. We also show the gene names for all genes that belong to an OG found in more than one GWAS dataset.

On another note, we found one OG related to fluconazole resistance in both *C. glabrata* and *C. auris* affecting the orthologues of *S. cerevisiae NRG1* and *NRG2*, respectively, both of which are transcriptional repressors. These *NRG1* and *NRG2* convergent associations suggest that this is a drug-resistance mechanism that is conserved across species. This is consistent with the fact that both *NRG1*- and *NRG2*-null mutants impact azole resistance and biofilm formation in *S. cerevisiae*^73,74^. To validate these high-confidence hits, we investigated whether equivalent genotype–phenotype associations were detected in independent datasets. This was the case for most genes (18/22, 82%) belonging to OGs with GWAS hits in more than one drug–species combination, further confirming the importance of these novel gene families (Supplementary Results and Extended Data Fig. 10). In summary, we detected several lesser-known gene families associated with resistance in multiple datasets, which illuminate core and conserved functions related to antifungal drug resistance. These results may guide future confirmatory experimental work, which is necessary and could provide information on the most important drivers as well as suggest relevant therapeutic targets.

## Conclusion

Understanding human-associated adaptation in pathogens is a long-standing question because it underlies virulence, hospital transmission and drug-resistance mechanisms. Our current knowledge is limited due to insufficient sampling, a lack of multispecies studies as well as an exclusive focus on SNPs and on specific genes. We have addressed these gaps in six major *Candida* species by analysing the publicly available genomes and phenotypes of approximately 2,000 (mostly clinical) strains. Our collection is a valuable resource due to its unprecedented size, the common analysis framework in multiple species, the consideration of complex variants (SVs and CNVs) and the availability of phenotypes. This underscores the value of depositing genomic and clinical data in public repositories that can be mined to generate new knowledge.

First, we used the generated variants to find genes affected by recent potentially clinically relevant selection. We found hundreds of affected gene families and pathways, mostly species-specific, suggesting highly variable, multifactorial adaptive mechanisms. In addition, we predicted novel conserved adaptive processes involving drug resistance and cell-adhesion functions, which are interesting pan-fungal therapeutic targets. We next analysed the variants, genes and pathways associated with clinical resistance towards all major antifungal drugs in three *Candida* species. Beyond confirming the implication of known drivers of resistance, which validates our approach, our results identified potential novel players related to adhesion, biofilm formation and transcriptional regulation. These novel mechanisms involve genes underlying cross-resistance towards multiple drugs of the same species and also gene families driving resistance in multiple species. Beyond the general trends discussed here, our catalogue of selection signatures and drivers of drug resistance is valuable to validate gene functions inferred from non-clinical studies (such as drug-resistance genes predicted from in vitro evolution). Finally, our analyses reveal an important role of the generally neglected complex variants (CNV and SV) and suggest an unexpected involvement of (para)sexual recombination in the spread of resistance mechanisms.

In summary, we provide novel insights and valuable resources that improve our understanding of selection and drug resistance across major *Candida* pathogens. Our findings may guide future confirmatory experiments, which could improve therapeutic and diagnostic options.

## Methods

### Generation of the filtered variant-calling dataset for each *Candida* species

We used the NCBI SRA toolkit (v2.10.9; https://github.com/ncbi/sra‑tools) to download all paired-end whole-genome re-sequencing datasets for the NCBI taxon identifiers^75^ related to each species (*C. albicans*, *C. auris*, *C. glabrata*, *C. tropicalis*, *C. orthopsilosis* and *C. parapsilosis*) from the NCBI SRA database^76^ (accessed 9 June 2020). For each sequencing run, we used fastQC (v0.11.9; https://www.bioinformatics.babraham.ac.uk/projects/fastqc) and trimmomatic (v0.38)^77^ with default parameters to remove adaptors and trim the reads. Finally, we ran perSVade (v0.6)^78^ to align (with BWA MEM (v0.7.17); http://bio-bwa.sourceforge.net/bwa.shtml) the trimmed reads to the reference genome (included in Supplementary Table 1) and calculate the coverage per window using mosdepth (v0.2.6)^79^. We filtered out low-quality runs with a read depth of <40× or <90% coverage of the reference. Note that a few of these runs could be redundant, as a given strain may have been re-sequenced multiple times. Accordingly, we found that 2.64% of all strains (as annotated in the NCBI SRA; see Supplementary Table 1) have multiple runs. However, we consider strain annotations to be impractical as unique identifiers of biological samples given that strain information can be missing or inaccurate in the NCBI SRA record. For instance, different clonal isolates from a given patient may have equal strain annotations, although these are clearly different biological samples. In addition, many strain names in the NCBI SRA are alphanumeric identifiers that do not correspond to standard strain definitions. Thus, we decided to use ‘sequencing runs’ as a proxy for isolates/strains and throughout the paper we use them indistinctly.

We next used the aligned reads to call variants using perSVade (v0.6)^78^, which calls and functionally annotates SNPs, small indels, CNVs and SVs. Structural variants are complex variants for which we could find the precise underlying rearrangements (such as tandem duplications, inversions or balanced translocations). Conversely, CNVs are variants generating large (>600 base pairs (bp)) duplications and deletions (inferred from changes in read depth) with unknown underlying rearrangements. Technically, CNVs are a type of SV but we differentiate them because the method used to infer them is different, and some CNV-like SVs (e.g. tandem duplications) may be detectable with the coverage-based method but not with the SV-detection method. By considering these two types of variants, we provide a comprehensive characterization of SVs. Note that any CNV that had an equivalent SV was not considered.

The small variant-calling pipeline integrates the results of three callers—that is, GATK Haplotype Caller (v4.1.2)^80^, freebayes (v1.3.1)^81^ and BCFtools (v1.9; https://github.com/samtools/bcftools). The CNV-calling pipeline detects deletions and duplications from coverage alterations using two algorithms—HMMcopy (v1.32.0)^82^ and AneuFinder (v1.18.0)^83^. The SV-calling pipeline finds rearrangements with GRIDSS (v2.9.2)^84^ (which uses split reads, discordantly paired reads and de novo assembly signatures) and summarizes them into actual SVs using CLOVE (v0.17)^85^. The called SVs are tandem duplications, deletions, inversions, translocations, copy–paste insertions, cut–paste insertions, inverted copy–paste insertions, inverted cut–paste insertions, inverted translocations and unclassified breakpoints (Extended Data Fig. 1). In addition, perSVade automatically selects the optimal GRIDSS- and CLOVE-filtering parameters for each sample based on simulations of SVs, which is useful for *Candida* species (where SV callers have not been tested extensively). PerSVade also integrates SVs and CNVs, which may be partially redundant, so that any CNV overlapping an equivalent SV would be discarded. Finally, this pipeline uses VEP (v100.2)^86^ to annotate the functional effect of each variant and RepeatModeler (v2.0.1)^87^, followed by RepeatMasker (v4.0.9)^88^ to annotate which variants overlap repeats. Note that for the functional annotation, we used the general feature format (GFF) files corresponding to each genome (included in Supplementary Table 1), with the exception of *C. tropicalis* and *C. parapsilosis* (which lacked annotations of the mitochondrial DNA). For these two species, we generated the mitochondrial DNA annotations using AUGUSTUS (v3.2.3)^89^ with default parameters and ‘candida_albicans’ as the train species.

We ran perSVade with custom parameters adapted to either haploid (*C. glabrata* and *C. auris*) or diploid (*C. albicans*, *C. tropicalis*, *C. parapsilosis* and *C. orthopsilosis*) species. For small-variant calling, we used ‘–ploidy 1–run_ploidy2_ifHaploid’ for haploid species, which runs the calling in both haploid and diploid mode or ‘–ploidy 2’ for diploid species, and ‘–coverage 12’ to discard positions with a read depth of <12×. Note that we ran the variant calling in diploid mode for the haploid organisms to take into account that they may have heterozygous variants in duplicated regions. For CNV calling, we used ‘–window_size_CNVcalling 300’ to call CNVs based on windows of 300 bp and ‘–min_CNVsize_coverageBased 600’ to discard CNVs <600 bp. For SV calling, we used ‘–min_chromosome_len 100000’ (to use only large chromosomes for SV simulations), ‘–simulation_ploidies auto’ (which results in parameter optimization based on haploid SVs for haploid species or heterozygous SVs for diploid species) and ‘–range_filtering_benchmark theoretically_meaningful_NoFilterRepeats’ (to run parameter optimization without filtering out repetitive elements). In addition, we used a custom function (‘get_integrated_SV_CNV_df_severalSamples’ (v0.6)^78^) from the perSVade source code to integrate the CNVs and SVs from different samples in a way that equivalent variants get the same identifier. This is not a trivial task given that the algorithms used often lack single-bp resolution and thus the same variant in different samples may get slightly different coordinates. To solve this, the get_integrated_SV_CNV_df_severalSamples function from perSVade uses bedmap from the bedops suite (v2.4.39)^90^ to cluster variants from the same type that reciprocally overlap by >75% of their total length and where their breakpoints are <50 bp from each other. In addition, we ran perSVade with custom NCBI translation codes (https://www.ncbi.nlm.nih.gov/Taxonomy/Utils/wprintgc.cgi) to perform functional variant annotations. We set the genomic DNA code to one for *C. glabrata* (standard code) and 12 for *C. albicans*, *C. tropicalis*, *C. parapsilosis*, *C. auris* and *C. orthopsilosis*. We set the mitochondrial DNA code to four for *C. albicans*, *C. tropicalis*, *C. parapsilosis* and *C. orthopsilosis*, and three for *C. auris* and *C. glabrata*. This procedure yielded the raw variant calls and their corresponding functional annotations. We discarded all runs where any of these steps (read trimming, alignment or variant calling) could not be performed due to file truncation or incompatible file formats.

To get the high-confidence variants, we applied extra filtering to discard artifacts. For small variants, we kept variants that passed the filters in at least two callers and where the fraction of reads covering the variant was ≥0.9 (for haploid configuration) or ≥0.25 (for diploid configuration). For CNVs, we filtered variants based on both the predicted relative copy number (which in a diploid may be zero for a homozygous loss, 0.5 for a heterozygous loss, 1.5 for a trisomy and 2.0 for a tetrasomy) and the relative coverage (measured as the ratio between the median coverage of the region under CNV and the median coverage across the whole genomic DNA). For deletions, we required copy number = 0 and relative coverage ≤ 0.1 for haploid species, and copy number ≤ 0.5 and relative coverage ≤ 0.6 for diploid species. For duplications, we required copy number ≥ 2.0 and relative coverage ≥ 1.7 for haploid species, and CN ≥1 .5 and relative coverage ≥ 1.3 for diploid species. For SVs, we calculated the variant allele frequency (VAF; as in https://github.com/PapenfussLab/gridss/issues/234) for each breakend forming each SV to discard variants with low VAF that may not be real haploid/diploid events. We kept SVs fulfilling two criteria: (1) they should have at least one breakend with VAF ≥ 0.8 for haploid species or VAF ≥ 0.3 for diploid species and (2) all breakends should have VAF ≥ 0.2 for haploid species or VAF ≥ 0.1 for diploid species. These filters yielded the high-confidence variant calls used in this paper. Note that for haploid species, we used the small variants called in haploid configuration in all analyses described below, except for the GWAS analyses, where we also used the heterozygous small variants from duplicated regions. In addition, this strategy assumes that all strains have the canonical ploidy of the species. Although the assumption may not be necessarily accurate in all cases, ploidy switches are rarely observed in such haploid species^91^ and we have no accurate way to infer ploidy directly from the sequences.

### Strain-tree generation

To reconstruct a phylogenetic tree for all strains of a given species, we used a different approach depending on the species ploidy. For haploid species, we generated a pseudo-genome sequence for each strain based on the reference genome but substituting the reference sequences according to filtered haploid SNPs. To avoid the biases introduced by CNVs and indels, these pseudo-genomes only included positions matching the following criteria in all strains: (1) ≥12× coverage, (2) absence of indels and (3) absence of heterozygous SNPs. In addition, we only considered variable positions. We used Biopython (v1.78)^92^ and bedmap to obtain the aligned pseudo-genomes, with 285,345 sites in *C. auris* and 311,174 sites in *C. glabrata*. We then obtained the unrooted tree, using IQ-TREE (v2.1.2)^93^, from these aligned pseudo-genomes using ‘-m TEST + ASC’ to use default automatic model selection and ascertainment bias correction (which is necessary to calculate meaningful branch lengths). Next, we used midpoint rooting (that is, no out-group was assumed) to obtain the final tree, which has support values from 1,000 bootstraps. Note that the heterozygous SNP patterns (along the tree) in these haploids were visually inspected to pinpoint runs that may be mixed or contaminated strains (which are expected to have many heterozygous SNPs). Two such *C. auris* samples that had heterozygous SNPs with a VAF of approximately 35% were found and discarded from subsequent analyses.

It was not possible to use an analogous method for diploid species due to the high heterozygosity in *C. albicans*^28^, *C. tropicalis*^24^ and *C. orthopsilosis*^36^. Inspired by refs. ^24,94^, we implemented a tree-generation method to take into account both homozygous and heterozygous SNPs. We generated 100 pseudo-genome sequences for each strain based on the reference genome but substituted the reference sequences according to filtered SNPs (only those that had defined heterozygous or homozygous genotype calls). These pseudo-genomes only included positions matching the following criteria in all strains: (1) ≥12× coverage and (2) absence of indels. All 100 pseudo-genomes included all homozygous SNPs and a random selection of heterozygous SNPs (each heterozygous SNP with a probability of 0.5 to be included). We then obtained one unrooted tree for each of these 100 aligned pseudo-genomes (with only variable positions) with IQ-TREE using ‘-m GTR+F+ASC+G4’ (equivalent to the ‘GTRGAMMA’ model used previously^24^), required to have a consistent model and ascertainment bias correction. The pseudo-genomes had 319,439–320,188 sites for *C. albicans*, 765,044–766,422 sites for *C. tropicalis*, 11,627–11,827 sites for *C. parapsilosis* and 575,685–576,053 sites for *C. orthopsilosis*. We rooted all 100 trees with midpoint rooting and generated a final consensus tree with branch lengths using IQ-TREE (-con argument), followed by the consensus.edges function from phytools (v0.7_90)^95^. Note that the branch support for this consensus tree was derived from the number of re-sampled trees including a given branch. Supplementary Table 1 includes all the used trees in Newick format. In addition, we provide the tree-generation pipeline as a stand-alone software package that can be useful (Code availability).

### Clade definition

To define meaningful clades in each tree, we first identified potential ‘clade-qualifying’ nodes as those with support ≥ 95 and long subtending branches (above a ‘min_relative_branch_length’ threshold). For a given min_relative_branch_length threshold, the clades would be clade-qualifying nodes where none of the children were also clade-qualifying nodes. We defined the ‘relative_branch_length’ for each node of each tree as the actual branch length normalized to the furthest distance between any two nodes. Thus, the min_relative_branch_length was the minimum relative_branch_length required for ‘clade-defining’ nodes. Note that the choice of a meaningful value for min_relative_branch_length was not trivial, and some values may leave out many strains without an assigned clade. To identify a reasonable min_relative_branch_length for each tree, we tried a range of values (between 0.001 and 0.2) and calculated, for each value, the total number of clades and the fraction of samples assigned to some clade. The final min_relative_branch_length was defined as the value that maximized the number of samples with a clade and minimized the total number of clades. We were able to find such optimal values, resulting in 4–24 clades (depending on the species) and >90% of strains within some clade for all species (Supplementary Fig. 1a).

To evaluate our clade definition, we compared it with previous population genomics studies for *C. albicans*^28^, *C. auris*^11^, *C. glabrata*^12^, *C. tropicalis*^24^ and *C. orthopsilosis*^36^ (Fig. 1b). Most clades (21/21 in *C. albicans*, 22/24 in *C. glabrata*, 4/5 in *C. orthopsilosis*, 2/4 in *C. auris* and 2/3 in *C. tropicalis*) were determined to be new (the strains within the clade were not included in the previous study) or to have a one-to-one strain correspondence with the previous study. To verify the absence of artifactual clades, we manually inspected the inconsistencies (Supplementary Table 1). We found that our clades 15 and 8 from *C. glabrata* were grouped into clade 5 in ref. ^12^, but our larger dataset provides higher resolution supporting the split of this clade in two. This is consistent with previous reports suggesting that clade 5 from ref. ^12^ is polyphyletic^29^. In addition, we found that one *C. auris* strain (SRR10852068) had been previously assigned to clade 3 (ref. ^11^) but it appears as clade 2 in our analysis (clade 1 in ref. ^11^), which suggests that there may have been a previous^11^ misclassification. This means that our clade definition in *C. auris* is fully consistent with ref. ^11^, except for this strain. Furthermore, we found that our tree topology in *C. orthopsilosis* is different around clade 4 (compared with ref. ^36^), resulting in some unclassified samples. Finally, we describe three highly divergent clades in *C. tropicalis* (Figs. 1b and 2a), whereas a previous study^24^ only assigned clades for one of them (our clade 3). This explains the inconsistency in our clade assignment. Together, these findings suggest that our clade assignments are largely consistent with previous findings. Supplementary Table 1 lists all current and former clade assignments.

### Generation of the strain metadata and definition of drug resistance

To obtain relevant metadata information (type of isolate and drug-susceptibility information) for all datasets with variant calls, we compiled two types of information. First, we used either the BioSampleParser package (https://github.com/angelolimeta/BioSampleParser) or Entrez-Direct utilities (v13.9)^96^ (only if BioSampleParser failed) to get the BioSample annotations (http://www.ncbi.nlm.nih.gov/biosample/) for each sequencing dataset. This provided the already accessible machine-ready metadata, including the strain identifiers. We then manually curated the literature associated with each of these strains to get the information about the strain type as well as the available drug-susceptibility information. From a total of 1,987 samples, we found 1,705 clinical isolates, 30 environmental strains, 49 genome-engineered strains, 201 strains from directed evolution experiments and 2 reference samples. We were able to find minimum inhibitory concentrations (MIC) or reports (statements in the literature) on susceptibility to amphotericin B (464 strains), beauvericin (five strains), 5-flucytosine (162 strains), terbinafine (one strain), miconazole (11 strains), ketoconazole (69 strains), isavuconazole (47 strains), voriconazole (250 strains), posaconazole (214 strains), itraconazole (151 strains), fluconazole (796 strains), micafungin (462 strains), caspofungin (463 strains) and anidulafungin (141 strains). To define discrete susceptibility profiles for each strain (susceptibility, S; intermediate susceptibility, I; resistance, R), we relied on either breakpoints for MIC data or direct reports of R or S (when MIC data were not available). We defined the breakpoints (BPs) for MICs based on either EUCAST recommendations (v10.0; https://www.eucast.org/), previous work^11,97,98^ or manually curated breakpoints based on our data (Supplementary Fig. 3). If MIC data were available, we defined each strain as R (MIC ≥ 2BP), S (MIC ≤ BP / 2) or I (BP / 2 < MIC < 2BP). Supplementary Table 1 includes all this metadata.

### Diversity analysis

To measure the pairwise genetic distance (number of variants kb^−1^) across all pairs of isolates in a given species, we counted the filtered variants unique to each strain of the pair. To measure the number of genes with protein-altering variants between each pair of isolates, we calculated the number of proteins that were altered by these unique variants (according to the functional annotation of perSVade). For small variants, we considered either haploid mutations (for haploid species) or both homozygous and heterozygous variants (for diploid species). For SVs and CNVs, we considered all variants.

We calculated the minor-allele frequency (MAF) of each haploid small variant, SV and CNV as MAF = (number of strains with variant)/(number of strains). This may be an oversimplification for SVs and CNVs but we considered it appropriate given that we could not get precise genotype calls for such complex variants. For each diploid small variant, we calculated it as:

$$\mathrm{MAF}=\left(\mathop{\sum }\limits_{i=1}^{n}{\mathrm{GT}}_{i}\right)/(\mathrm{number}\,\mathrm{of}\,\mathrm{strains})$$

Where *n* is the number of strains with the variant, *i* refers to the strain (from one to *n*) and GT_*i*_ is either 0.5 (for heterozygous calls) or 1.0 (for homozygous variants). Note that we only considered diploid small variants with a genotype call (homozygous or heterozygous) that was consistent across all algorithms that identified a given variant. In addition, only MAFs for variants with a MAF < 0.5 were considered. Extended Data Fig. 1b includes the MAF distributions.

### Investigating mechanisms of SV formation

To understand the mechanisms of SV and CNV formation, we first investigated whether each variant overlaps RepeatMasker annotations^88^. We extracted the regions under SVs and CNVs (duplicated, inverted, deleted or translocated) and ran RepeatMasker on them using standard libraries and species-specific RepeatModeler^87^ libraries. The module ‘infer_repeats’ of perSVade^78^ was used to run these programmes. If ≥10% of the altered region (duplicated, inverted, deleted or translocated) was covered by a RepeatMasker annotation, this was considered as the formation mechanism. These included insertions of transposable elements and expansions or contractions of transfer RNA, rRNA or simple repeats. We could not find such overlaps for most variants (Extended Data Fig. 2), which suggests that other mechanisms are essential for SV and CNV formation. For all of the remaining variants, we investigated the role of homologous regions in SV formation, which could be relevant^37,38^. We checked whether each variant had breakpoints with exact microhomology (2–10 bp are identical), inexact microhomology (2–10 bp are similar), exact homology (>10 bp are equal) or inexact homology (>10 bp are homologous) between the breakends. Variants with microhomology may have been generated by microhomology-mediated end joining (a double-strand-break-repair pathway), and variants with long homology could be attributable to meiotic non-allelic homologous recombination^37^. If none of these signatures were found, we classified the variant as ‘other’, which may be related to non-homologous end joining to repair double-strand breaks^37^. Note that we did not consider variants that were potentially biased by overlapping simple repeats and low-complexity regions for this analysis. For CNVs, such variants were those with simple repeats of low-complexity regions spanning ≥25% of the CNV (inferred with RepeatMasker), which may affect coverage calculations. For SVs, these were variants where at least one breakend overlapped any such repetitive elements, inferred with bedmap. Extended Data Fig. 2 includes the results of this analysis.

### Gene annotations

We obtained broad gene annotations (gene name, type of gene, location, description and *Saccharomyces cerevisiae* orthologues) from the *Candida* Genome Database (CGD) chromosomal feature files^99^ (available in Supplementary Table 1). The gene length was calculated from the GFF annotations, considering untranslated regions, if available. To get protein functional annotations, we first obtained the protein sequences by retrieving spliced transcripts from each GFF using gffread (v0.12.1)^100^ and then translating these transcripts using Biopython. We next ran Interproscan (v5.52-86.0)^101^ on these proteins with the arguments ‘-appl Pfam,ProSitePatterns,ProSiteProfiles,PANTHER,TIGRFAM,SFLD,SUPERFAMILY,Gene3D,Hamap,Coils,SMART,CDD,PRINTS,PIRSR,MobiDBLite,PIRSF’ (to run several annotation modules), --pathways (to get MetaCyc and Reactome annotations) and -goterms (to get automatic GO annotations). To obtain information on orthologous groups (hereafter referred to as ‘gene families’), we ran OrthoFinder (v2.5.2)^102^, with the arguments ‘-M dendroblast -S diamond’, on the proteomes of all *Candida* species. To get the set of GO annotations shown in all the tables, we mixed annotations from both Interproscan and CGD (see Supplementary Table 1).

We applied some extra steps to get the pathway annotations for GWAS and enrichment analyses (see below). To map each gene to the complete set of MetaCyc pathways, we took all annotations from Interproscan and added the parent pathways (using Pathway Tools (v25.0)^103^). MetaCyc pathways where the taxonomic range did not include Ascomycota were discarded. Similarly, to map each gene to the set of Reactome pathways, we took the Interproscan annotations and added the parents (using the files ReactomePathways.txt and ReactomePathwaysRelation.txt from https://reactome.org/download/current/; accessed 4 October 2021). Given that Reactome has several mammalian-specific pathways, we only kept annotations under the following groups: ‘Metabolism of proteins’, ‘Autophagy’, ‘Transport of small molecules’, ‘Gene expression (Transcription)’, ‘Cellular responses to stimuli’, ‘Reproduction’, ‘Digestion and absorption’, ‘Signal transduction’, ‘Extracellular matrix organization’, ‘DNA repair’, ‘Chromatin organization’, ‘Cell cycle’, ‘Metabolism’, ‘Organelle biogenesis and maintenance’, ‘DNA replication’, ‘Programmed cell death’, ‘Vesicle-mediated transport’, ‘Metabolism of RNA’, ‘Cell–cell communication’, ‘Protein localization’ and ‘DNA replication and repair’. In addition, we only considered pathways annotated for ‘*Saccharomyces cerevisiae*’ and ‘*Schizosaccharomyces pombe*’. Finally, to map each gene to all GO terms, we used both annotations from CGD and Interproscan, and added all the parent terms (using GOATOOLS (v1.1.6)^104^ and the obo file from http://purl.obolibrary.org/obo/go/go-basic.obo; accessed 30 June 2021). In addition, to ensure that the annotated terms are meaningful in each species, we only kept GO terms that were defined in some gene of the CGD-curated dataset (see Supplementary Table 1).

### Measuring signatures of recent selection

The measurement of selection in such population genomic data is often achieved through the use of sweep detection- or *π*_N_/*π*_S_-based (similar to dN/dS but for population genomic data^12,40^) methods^41^. *Candida* species mostly propagate clonally, which suggests that a *π*_N_/*π*_*S*_-based method (where synonymous SNPs reflect near-neutral evolution and can be useful to correct biases in mutation rates across genes) is more suitable to detect signatures of selection. However, standard approaches were unfit for our question because we wanted to measure recent selection for various variant types (discussed below in more detail). Thus, to understand the signatures of recent positive selection, we developed a custom method to identify genes that recently acquired non-synonymous or functional variants in a highly recurrent manner (variants appearing often in different parts of the tree). The sections below explain this method in detail.

#### Obtaining recent variants

To only consider recent variants, we defined monophyletic clusters of (likely) clonally propagating strains with a recent common ancestor (they should be under nodes with support ≥ 95 where all leaf strains have ≤1 SNP kb^−1^ to each other). The pairwise SNP kb^−1^ values were calculated using the approach described in the ‘Diversity analysis’ section; however, positions with <12× coverage in any strain were discarded (using mosdepth and bedmap). This 1 SNP kb^−1^ threshold was not trivial to set, as a high threshold may group very divergent strains together, and a low threshold may leave many strains without a cluster and would thus not be considered by our analysis. We tested this trade-off for several thresholds and found that 1 SNP kb^−1^ was a reasonable value, where most strains were classified into some cluster (98% in *C. glabrata*, 99% in *C. auris*, 78% in *C.* *tropicalis*, 59% in *C. albicans*, 100% in *C. parapsilosis* and 36% in *C. orthopsilosis*; Supplementary Fig. 1b,c). Note that the large fraction of unassigned *C. orthopsilosis* samples (64%) may limit our power to detect selection in this species. Next, we then ran ASR on all variants to define those that appeared after the diversification of each clonal cluster. For this, we ran Pastml (v1.9.34)^105^ with ‘–prediction_method ALL’ (to use the six available ASR methods) on each variant independently using the strain tree generated as described in the ‘Strain-tree generation’ section. To avoid having branches with a length of zero, we added a pseudocount to each branch length (10% of the shortest leaf with a non-zero branch length) for the ASR using ete3 (v3.1.2)^106^. Variants were considered as ‘recent’ in a given strain if they were not predicted to be present in the common ancestor of the clonal cluster by any of the ASR methods implemented in Pastml. Loss-of-heterozygosity events were not specifically considered.

#### Defining functional types of variants

To measure selection by different variant types, we grouped these recent SNPs, indels, CNVs and SVs into functionally equivalent categories according to the effects on coding regions (taken from the ‘Consequence’ field of perSVade). Non-synonymous SNPs (nsyn_SNPs) were SNPs with ‘stop_lost’ or ‘missense_variant’ consequences. Synonymous SNPs (syn_SNPs) were SNPs with ‘synonymous_variant’ or ‘stop_retained_variant’ consequences. In-frame indels (if_INDELs) were indels with ‘start_retained_variant’, ‘inframe_deletion’ or ‘inframe_insertion’ consequences. Duplications (DUPs) were SVs or CNVs with ‘transcript_amplification’ consequence. Deletions (DELs) were truncating small variants (with ‘stop_gained’, ‘protein_altering_variant’, ‘frameshift_variant’, ‘start_lost’ or ‘coding_sequence_variant’ consequences), gene-deleting SVs or CNVs (with ‘transcript_ablation’ consequence) or transcript-breaking SVs (with frameshift_variant, inframe_deletion, start_retained_variant, inframe_insertion, start_lost, stop_lost, coding_sequence_variant, protein_altering_variant, stop_gained, ‘5_prime_UTR_variant’, ‘3_prime_UTR_variant’, ‘splice_region_variant’ or ‘intron_variant’ consequences). Our selection detection method identified genes with either an excess of recurrent nsyn_SNPs (using syn_SNPs to correct for neutral evolution) or with particularly high numbers of recurrent if_INDELs, DUPs and DELs (see below). We thus only considered protein-coding genes with no pseudogene annotation (according to the chromosomal feature files from CGD; ‘Gene annotations’ section). In addition, we discarded all variants that were potentially biased by overlapping simple repeats and low-complexity regions for this analysis. For CNVs, these variants were those with simple repeats of low-complexity regions spanning ≥25% of the CNV (inferred using RepeatMasker), which may affect coverage calculations. For SVs and small variants, these were variants where some part of the variant overlapped any such repetitive elements, as inferred with bedmap.

#### Finding genes under selection by non-synonymous SNPs

To find genes under selection by non-synonymous SNPs, we implemented a selection detection method inspired by the *π*_N_/*π*_S_ (ratio between non-synonymous (π_N_) and synonymous (π_S_) diversity) approach, where synonymous SNPs reflect neutral evolution and can be useful to correct biases in mutation rates across genes. Given our focus on the few recent variants that appeared within clusters of clonal strains, we considered that we had insufficient mutations to infer selection based only on raw *π*_N_/*π*_S_ values, as is commonly done^12,29^. As synonymous SNPs are the least common, strains with some adaptive non-synonymous variants (*π*_N_ > 0) may have *π*_S _= 0, which does not allow for *π*_N_/*π*_S_ calculations. In addition, even in strains with some synonymous SNP, the low variant counts would probably result in inaccurate *π*_N_/*π*_S_ calculations due to single variants dramatically changing the ratio. Thus, we reasoned that we lacked resolution to detect selection for a given gene in each strain, as previously done when considering all (not only recent) variants^29^. We instead devised a strategy to measure average per-gene selection pressures. Given the inaccurate nature of such *π*_N_/*π*_S_ values, we considered that simply measuring the average *π*_N_/*π*_S_ for a given gene across all strains (as done previously^12^) may not be appropriate for our purposes. These constraints justified the need for a novel method that was more suited to detect recent selection.

To solve this, we use alternative metrics and an empirical statistical method to pinpoint genes with an excess of recurrent non-synonymous SNPs. To avoid problems with solely relying on *π*_N_/*π*_S_ calculations, but still capture average selective pressures, we defined genes with *π*_N_ > *π*_S_ in a high number of strains and clusters (higher than expected under an empiric model of neutral evolution) as genes under recent selection (Extended Data Fig. 3). For each gene, we define ‘strains under selection’ as those with a *π*_N_ > *π*_S_, which suggests accelerated evolution and potentially positive selection^35^. We then calculated the selection score (*S*) for each gene as the harmonic mean between the fraction of strains under selection (*π*_N_ > *π*_S_) and the fraction of clusters that have a strain under selection. We used the harmonic mean ($h(x,y)=(2\cdot x\cdot y)/(x+y)$) because it is a value between zero and it is only high if both values are high. This ensures that genes with high *S* values have *π*_N_ > *π*_S_ in several strains and clusters, suggesting that they bear the strongest signatures of recent selection. In addition, by considering both the number of strains and the number of divergent clusters, we corrected for possible stochastic errors derived from biased sampling of some clades and/or recent clonal population expansions could be unlinked to selection. We calculated diversity (*π*_N_ or *π*_S_) for each gene in each sample as:

$$\mathrm{Diversity}(\pi )={n}_{\mathrm{recent},\mathrm{gene}}/(c\cdot f)$$

where *n*_recent,gene_ is the number of recent SNPs (either non-synonymous for *π*_N_ or synonymous for *π*_S_), *c* the length of the coding sequence (CDS) that does not overlap repeats or low-complexity regions and *f* is either 0.75 for *π*_N_ or 0.25 for *π*_S_. Note that *f* is a normalization parameter to take into account that synonymous variants are less likely to happen and we set the *f* as done previously^12^. We used the bedtools (v2.30.0)^107^ ‘subtract’ and ‘merge’ modules to calculate the CDS lengths. Note that we considered that diploids have two copies of each gene (*c* is twice the annotated CDS length), so that heterozygous SNPs add one to *n*_recent,gene_ and homozygous SNPs add two.

One of the biases for the *S* calculation is that, given that we considered only recent variants, the *π*_N_ and *π*_S_ values could be low or zero for some genes, leading to high *S* values due to stochastic biases from low variant counts. To provide a statistical framework and find genes with significantly high *S* values, we calculated the empiric probability (*P*) that a gene has a *S* greater than or equal to that observed under a neutral model of evolution. To do this, we obtained a distribution of *S* values generated randomly (on the same strains used to calculate the real *S*) by a model considering the neutral mutation rate of each gene. We used synonymous SNPs as a proxy for such a neutral mutation rate. To calculate a synonymous SNP mutation rate (*r*_S_), we used information from all the synonymous SNPs (not only recent variants) present in each strain, so that *r*_S_ is defined (for each gene) as:

$${r}_{\mathrm{S}}=\mathrm{mean}\left({n}_{\mathrm{all,gene}}/{n}_{\mathrm{all,all}}\right)$$

This reflects a mean mutation rate across strains, where *n*_all,gene_ is the number of all synonymous SNPs in the gene for a given strain and *n*_all,all_ is the number of all synonymous SNPs in any gene. For calculating *r*_S_ in each gene, we only used strains with *n*_all,gene_ ≥1 and *n*_all,all_ ≥10 (good strains), and we filtered out genes with <3 good strains. We assume that the synonymous mutation rate per gene is similar across all strains and between recent and ancestral variants (those that appeared before the cluster diversification). Under these assumptions, *r*_S_ represents the probability of having a synonymous SNP in the gene for each synonymous SNP in any gene. In addition, assuming that non-synonymous SNPs are three times more frequent than synonymous SNPs, we defined a ${r}_{\mathrm{N}}=3\cdot {r}_{\mathrm{S}}$, which represents (under neutral evolution) the probability of having a non-synonymous SNP in the gene for each synonymous SNP in any gene.

We used these probabilities to generate random numbers of recent SNPs (expected by neutral evolution) from a binomial distribution where *n*_recent,all_ (for a given strain, the total number of recent SNPs in any gene) is the ‘number of tries’ and *r* is the ‘probability of SNP for each try’. For each gene and 10,000 samples we generated, in each strain:

$$\mathrm{random}\,\mathrm{neutral}\,\mathrm{diversity}\,({\pi }_{\mathrm{R,i}})=\mathrm{binomial}({n}_{\mathrm{recent},\mathrm{all}},r)/(c\cdot f)$$

where *i* reflects the sample index (from 1 to 10,000), *r* is *r*_N_ for non-synonymous random neutral diversity (*π*_N,R,i_) or *r*_S_ for synonymous random neutral diversity (*π*_S,R,i_), *c* is the length of the CDS that does not overlap repeats or low-complexity regions and *f* is either 0.75 for *π*_N,R,i_ or 0.25 for *π*_S,R,i_. We then calculated, for each gene and each sample, a random neutral selection score *S*_R,i_ as the harmonic mean between the fraction of strains under ‘selection’ (*π*_N,R,i_ > *π*_S,R,i_) and the fraction of clusters that have a strain under ‘selection’. We calculated the final empirical probability *P*(*S*), which indicates the likelihood of observing a given *S* under neutral evolution, as:

$$P(S)=\left(\mathop{\sum }\limits_{i=1}^{10,000}1\,\mathrm{if}\,({S}_{\mathrm{R,i}}\ge S)\right)/10,000$$

To validate this neutral model, we reasoned that the observed *π*_S_ values (considering recent variants) should fall within the neutral distribution of *π*_S,R,i_. We thus calculated, for each strain, whether the observed *π*_S_ is extreme in the neutral distribution (>95% of samples with *π*_S,R,i_ > *π*_S_ or >95% of samples with *π*_S,R,i_ < *π*_S_). We found that most strains in the majority of genes have non-extreme *π*_S_ values (Extended Data Fig. 3c), suggesting that the null model is generally reasonable. To discard possible biases, we filtered out genes where ≥10% of strains had such extreme *π*_S_ values. In addition, to discard genes with low variability, we only considered genes with *π*_N_ > *π*_S_ in ≥2 clusters and ≥3 strains.

Finally, genes with convergent signs of recent positive selection by non-synonymous SNPs were defined as those with an FDR-corrected *P*(*S*) < 0.05.

#### Finding genes under selection in-frame indels, duplications and deletions

To find genes where in-frame indels, duplications and deletions are selected, we implemented a different approach given that the concept of synonymity does not apply here. For each gene and variant type (in-frame indels, duplications and deletions), we calculated the *S* score as the harmonic mean between the fraction of strains that have a recent variant and the fraction of clusters that have a strain with a variant. Genes with high *S* values are likely to be those with the most frequent recurrent variants, suggesting selection on them. To discard genes with low variability, we only considered genes with recent variants in ≥2 clusters and ≥3 strains. Finally, we defined genes under selection by these variants as those with an *S* above the 90th percentile of considered genes (Extended Data Fig. 4b). One limitation of this approach is that recurrent variant acquisition could be sometimes unrelated to selection, given that some genomic regions may have higher mutation rates for these types of variants. However, given our focus on functional variants we consider it a valid proxy to identify genes potentially under recent selection. In addition, an observation of an excess of recurrent deletions (in deletion genes) may reflect relaxation of purifying selection rather than positive selection. Nevertheless, we consider that deletion genes inform about the process of recent adaptation in *Candida* pathogens and thus included them in our analyses.

#### Strain filtering

We filtered out some strains to get meaningful *S* score calculations. To ensure that the inferred genes may be under clinically relevant selective processes (like adaptation to the host, hospital environments or antifungal drugs), we only considered clinical isolates. To avoid biases derived from low coverage and pseudogenization, we filtered out some strains for each gene. For non-synonymous SNPs and in-frame indels, we wanted to discard strains where the gene may be broken, so that we required the following criteria to accept strains: (1) ≥24× median coverage, (2) ≥95% of the gene is covered and (3) absence of truncating small variants or transcript-breaking SVs or CNVs (defined earlier). This ensured that the definition of ‘excess of nsyn_SNPs and/or if_INDELs’ is only based on isolates where the gene was complete and with proper coverage (also with no deletion mutations). For deletions and duplications, we wanted to consider strains where the cluster’s ancestor had the intact gene, so that we required the absence of truncating small variants or transcript-breaking SVs or CNVs in the ancestor (by all ASR methods used here). In addition, to ensure that all strains used had some degree of divergence to measure the *S*, we only considered strains that acquired at least one synonymous SNP in any gene after the cluster diversification.

The list of genes under positive selection by different variant types is in Supplementary Table 2, and Fig. 3a includes a cartoon that explains how selection was calculated. Note that Supplementary Table 2 includes both the genes under selection as well as the *S* selection scores and *P* values for all genes in which *S* could be calculated.

#### Validation of the clinical relevance of the recent selection signatures

To validate that these selection signatures often reflect clinical adaptation, we checked the overlap between genes with recurrent mutations across clonal serial isolates and those under recent selection (Supplementary Results). We first mined the literature associated with our dataset to pinpoint pairs of serial clinical isolates from the same patient (Supplementary Table 1). To discard redundancies and ensure that the compared strains were truly serial, we only kept one run for each time point in each patient (the one with the highest average coverage). For each pair of serial isolates, we then identified non-synonymous SNPs, in-frame indels, duplications and deletions (defined as described earlier) that appeared in the latter isolate and we only considered pairs of isolates with <1 new SNP kb^−1^ for further analysis. In addition, for a given gene, we only considered pairs of isolates that met a quality control criteria matching the strain filtering used in the recent selection analysis (‘Strain filtering’ section). For instance, for non-synonymous SNPs and in-frame indels mutations, we only considered pairs where both isolates had (1) ≥24× median coverage, (2) ≥95% of the gene covered and (3) absence of truncating small variants or transcript-breaking SVs or CNVs. Similarly, for deletion and duplication variants, we only considered pairs where the first isolate had the gene meeting these three criteria. Finally, we identified ‘genes with signs of selection’ in these serial isolates as those that had a new variant in at least two pairs.

To test whether there was a significant overlap between these genes and those under recent selection (according to the approach defined in Fig. 3 and in the earlier subsections of ‘Measuring signatures of recent selection’), we considered, for each species and type of variant, whether each gene (out of all protein-coding genes) belongs to any of the following categories:

**Table:** 

		Gene under recent selection	
		Yes	No
Gene recurrently mutated in serial isolates	Yes	w	y
	No	x	z

We used statsmodels (v0.11.1) to perform a one-sided Fisher’s exact test (where the null hypothesis is that there is no positive association between these two sets of genes) for each of these tables. We considered that if *P* < 0.05 was obtained for this test, it reflects significant enrichment of clinical selection signatures in the genes under recent selection defined here.

### Calculating the significance of the overlaps between orthologous groups

We used an empirical approach to calculate the significance of the overlap between OGs with genes under selection between either pairs of species or pairs of variant types of a given species (Fig. 3 and Supplementary Results). We tried to answer the following question: if we observe *O* overlapping OGs between two sets of *n*,*m* genes (i.e. *n* genes under selection in *C. glabrata* and *m* genes under selection in *C. auris*), what is the empirical probability (*P*(*O*)) of having an overlap ≥*O* when randomly sampling genes? To answer this question for each pair of *n*,*m* gene sets (to compare), we generated 10,000 sets of randomly sampled *n*_*i*_*,m*_*i*_ genes. For each pair of random gene sets, we obtained the corresponding OGs and calculated the number of overlapping groups *O*_*i*_. We then calculated *P*(*O*) as:

$$P(O)=\left(\mathop{\sum }\limits_{i=1}^{10,000}1\,\mathrm{if}\,({O}_{i}\ge O)\right)/10,000$$

For example, there are 25 genes (*n* = 25) under selection by deletions (from 21 OGs) and 92 genes (*m* = 92) under selection by SNPs (from 90 OGs) in *C. glabrata* (Fig. 3b and Supplementary Table 2). There are six OGs with genes under selection by both SNPs and deletions (*O* = 6), and the probability *P*(6) of having ≥6 overlapping OGs when taking 25 and 92 random genes is 0.0001. We consider this overlap significant because *P* < 0.05.

### Functional enrichment of genes under recent selection

To get the domains and pathways enriched in genes under selection, we ran a Fisher’s exact test on each gene set (selected in each species, by each variant type) for all relevant (described earlier) GO terms, Reactome, MetaCyc pathways and Interproscan annotations (a proxy for domains). We defined enriched groups (pathways or domains) as those with raw *P* < 0.05, FDR-corrected *P* < 0.05 and odds ratio ≥ 2. Note that we ran the FDR correction independently for the following sets of groupings: domains, Reactome, MetaCyc pathways, GO biological process, GO molecular function and GO cellular component terms. We used statsmodels (v0.11.1)^108^ to perform the Fisher’s test and FDR correction. Supplementary Table 2 includes the results of these enrichments. For all pathway types (MetaCyc, Reactome and GO), we discarded very general annotations (they are in 25% of genes).

To visualize the enriched groups across different species and variant types (Fig. 4 and Supplementary Fig. 2), we performed some clustering of the groups for easier interpretation. For domains, Reactome and MetaCyc pathways, we mapped each gene to the OGs and hierarchically clustered the groups (see domains in Supplementary Fig. 2) according to the Jaccard distance between OG sets in different species.

To visualize only a subset of representative GO terms (out of significant terms in all species; Fig. 4), we performed a redundancy-reduction step inspired by the REVIGO algorithm^109^. To define these representatives, we iterated through all pairs of terms with a Lin semantic similarity^104^ of ≥0.5 (pairs sorted by descending similarity). For each pair of terms, we defined a ‘rejected’ (non-representative) term following a hierarchical algorithm. If one term was very general (the median percentage of genes with that term (across species) was ≥5%) and the other was not, we rejected the general term. Alternatively, if the terms had clearly different *P* values (the median *P* across species of one term was less than half of the median *P* of the other,) we rejected the term with the highest *P*. Alternatively, if one term was a parent of the other, we rejected the child unless both terms were similar (the Jaccard index between the children of both terms was ≥0.75). If none of these conditions were met, we rejected the second term after numeric sorting of the GO identifiers. At the end we defined ‘representative terms’ as those that were not rejected in any pairwise comparisons. For each non-representative term, we assigned the representative term as the closest representative term (in terms of Lin’s semantic similarity). The output of this process is shown in Fig. 4, where each row is one representative term (hierarchically clustered by semantic similarity) and the *P* value is the lowest across all significant terms (in each species-type variant) mapped to that representative. This visualization ensures that similar significant GO terms appear in the same row, improving the interpretation of the shared functions under selection in different species and types of variant. Note that the key change from the original REVIGO is that our algorithm can select representatives across sets of GO terms from different species.

### Convergence-based GWAS

To find the variants underlying resistance, we performed a convergence-based GWAS. Briefly, we used ASR on each variant and the drug-resistance phenotype to find nodes of the strain tree with variant and/or phenotype transitions (Fig. 5a). Nodes with variant or phenotype transitions are those that acquired or lost the variant or resistance phenotype when compared with the parent node. We identified variants whose transition is statistically correlated with the transition in the drug-resistance phenotype. The following sections describe in detail how we ran this analysis.

#### Selecting strains and building a tree for each species and drug

To maximize our power to detect variant–phenotype associations, we treated drug resistance as a dichotomous trait and only analysed strains with either strong resistance (R strains) or strong susceptibility (S strains; ‘Generation of the strain metadata and definition of drug resistance’ section and Supplementary Fig. 3), discarding intermediate phenotypes. In addition, to make sure that the associations are clinically relevant, we only considered clinical isolates. We only ran the GWAS pipeline for drugs with ≥5 R and S clinical strains in a given species, which we could find for *C. albicans*, *C. glabrata* and *C. auris*. Note that although we could find ≥5 R and S strains for *C. tropicalis*–caspofungin, we decided to not perform a GWAS on this dataset due to potential technical biases. The resistance breakpoint for this drug was based on calculating the MIC at 24 h in a liquid-based assay with multiple concentrations^97^. However, the susceptibility data come from growth after 48 h in solid plates in only three concentrations^24^. Thus, although the susceptibility data may be interesting for other purposes, we considered that in this case the S/R discretization may not be accurate and thus did not perform a GWAS for *C. tropicalis*–caspofungin. Further experimental and sequencing efforts are needed to increase available data and investigate the determinants of clinical antifungal resistance in *C. tropicalis*, *C. orthopsilosis* and *C. parapsilosis*.

To have a balanced set of R/S isolates and reduce redundancy, we first pruned the strain tree to keep only R/S strains and then selected three representative isolates for each monophyletic node (where all strains are either R or S). To select these representatives, we performed a multidimensional scaling representation of all strains within a monophyletic node based on pairwise branch distances using sklearn (v0.24.2)^110^ and chose the three strains that are closest to uniformly spaced along the first axis of the multidimensional scaling. This strategy ensured that the representative strains included the highest diversity possible of each monophyletic node. We then built one tree for each species–drug combination considering only the representative R or S samples using the same pipeline as described in the ‘Strain-tree generation’ section. This tree was used to do the convergence-based GWAS.

#### Defining groups of variants for collapsed GWAS running

To define a set of variants for GWAS, we took all the filtered SNPs, indels, SVs and CNVs found in each sample. In addition, to take into account the role of aneuploidies (whole chromosome losses or deletions) in drug resistance, we defined aneuploid chromosomes as additional variants for GWAS testing. To identify aneuploidies, we used bedmap to find chromosomal windows (5,000 bp each) under duplication (if the median copy number based on called CNVs (copy number) was ≥1.8) or deletion (if the copy number was ≤0.2). Chromosomes that had ≥50% of windows under deletion or duplication were considered aneuploid. For small variants, we used a different set of variants depending on the ploidy of each species. For diploids, we kept both homozygous and heterozygous calls. For haploids, we kept all haploid variants and the diploid heterozygous variants from duplicated regions (positions with a copy number of ≥2 according to perSVade’s outputs).

To consider that different variants may drive similar resistance by altering the same genomic feature, we wanted to collapse variants into groups. This allowed us to test the association between the transition in any variant of a group and the phenotype transition. We collapsed variants taking into account the following: (1) the type of variant (‘all variants’, ‘small variants’, ‘CNVs’, ‘SVs’, ‘SVs and CNVs’, ‘small variants and SVs’ or ‘small variants and CNVs’), (2) the type of mutation (‘all mutations’, ‘non-synonymous’, ‘truncating’ or ‘non-synonymous that are not truncating’) and (3) the type of collapsing (at the level of ‘domains’, ‘genes’, ‘Reactome’, ‘GO’ or ‘MetaCyc’). We ran one GWAS for each combination of ‘variant type’, ‘type of mutation’ and ‘type of collapsing’, with the exception of domain and pathway-level collapsing, where we only considered types of mutations that were either non-synonymous, truncating or non-synonymous that are not truncating. Note that for the domain and pathway-level collapsing, we only considered protein-coding genes because these are the ones that we could map to such functional annotations. Finally, we ran a total of 113 GWAS analyses for each species and drug: one for the non-collapsed variants (where we tested each variant individually) and 112 for each combination of collapsing modes. For example, one of these GWAS analyses involved collapsing truncating SVs and small variants into genes (type of variant, small variants and SVs; type of mutation, truncating; and type of collapsing, genes), testing whether the truncation of each gene by small variants or SVs was correlated to the resistance. To avoid redundancy with the single-variant uncollapsed GWAS runs, we only considered groups with ≥2 variants.

To define this type of mutation, we used the perSVade functional annotations of each variant in each gene. Truncating variants were defined as those that had at least one of the following consequences on each gene: ‘stop_gained’, ‘protein_altering_variant’, ‘frameshift_variant’, ‘start_lost’, ‘coding_sequence_variant_BND’, ‘intron_variant_BND’, ‘non_coding_transcript_exon_variant_BND’, ‘transcript_ablation’, ‘non_coding_transcript_variant_BND’ and ‘coding_sequence_variant’. Note that 'BND' refers to 'break-end', so that consequences ending with '_BND' imply that there is a rearrangement overlapping certaing gene regions (i.e. introns or coding sequences). Non-synonymous variants were defined as those that had at least one of the following consequences on each gene: stop_gained, protein_altering_variant, frameshift_variant, start_lost, coding_sequence_variant_BND, intron_variant_BND, non_coding_transcript_exon_variant_BND, transcript_ablation, non_coding_transcript_variant_BND, ‘inframe_insertion’, coding_sequence_variant, ‘missense_variant’, inframe_deletion, stop_lost and transcript_amplification. Finally, variants wer defined as non-synonymous that are not truncating if they had non-synonymous consequences but no truncating consequence in a given gene.

To define the type of collapsing, we considered the gene, domain and pathway annotations as described in the section ‘Gene annotations’. For domain collapsing, we grouped variants overlapping each Interproscan annotation and also each window of 10, 25, 50 or 100 amino acids from all proteins. A variant was considered to have altered a domain if it overlapped it by at least 1 bp according to bedmap’s output. For example, we grouped all variants affecting a given domain from a gene and tested whether the transition in any of these variants was correlated to the phenotype transition. For gene collapsing, we grouped variants according to the consequences on genes annotated in the perSVade outputs. We thus tested whether the transition in any variant from a given gene correlated with the phenotype transition. Finally, for pathway collapsing, we extended the gene collapsing to the GO, Reactome and MetaCyc annotations. To avoid having variant groupings that were too general, we discarded pathways involving ≥5% of all genes in each species. For example, we grouped all variants affecting any gene from a given pathway together and tested whether the transition in any of these variants correlated with the phenotype transition.

#### Running the GWAS analysis

To measure the association of each group of variants (or single variants without grouping) to the resistance phenotype to each drug in each species, we used a custom pipeline inspired by the hogwash synchronous algorithm^58^. For simplicity, the paragraphs below mention ‘groups’ to indicate both groups of variants (that is, the ones that belong to a gene) or single variants.

One of the challenges of this analysis was that there are no studies in *Candida* species using similar convergence-based GWAS methods, suggesting that previous methods (designed for bacteria, like hogwash) may not be directly transferable. For example, hogwash used a maximum likelihood (ML) method to run ASR, but using maximum parsimony (MP) could be more accurate in some of our datasets. To address this, we ran the analysis using different parameters, changing the ASR methods, the branch-support thresholds and the methods to calculate empirical *P* values (see below). This allowed us to define the optimal parameters for our datasets, as described in the section ‘Filtering GWAS results’. The following paragraphs describe how we measured the associations by different parameter combinations.

The first step to run convergence-based GWAS was to infer ancestral states for all variants and resistance phenotypes. For this, we used the same ASR pipeline as described in the ‘Obtaining recent variants’ section but using the strain tree generated for each drug and species. This yielded, for different ASR methods, a state of one (presence of the variant or phenotype in the node), zero (absence of the variant or phenotype) or not available (NA; unknown state due to uncertain ASR results) in each node. To test the effect of different ASR methods (implemented in Pastml^105^), we considered the results from the marginal posterior probabilities approximation (MPPA) ML method, the DOWNPASS MP method and the consensus between the ML and MP methods. We defined the ML and MP consensus state as one (if both ML and MP were one, ML was one and MP was NA or ML was NA and MP was one), zero (if both ML and MP were zero, ML was zero and MP was NA or ML was NA and MP was zero) or NA if none of these conditions were met. In addition, to discard lowly supported branches, we set all nodes with a branch support below a ‘min_branch_support’ threshold (either 50 and 70) to NA states. This means that for each group, we ran six different association measurements using the ML, MP or ML and MP ASR methods and a min_branch_support of 50 or 70. Note that loss-of-heterozygosity events are not specifically considered.

To measure the association of each group to the resistance we identified the following types of nodes:

- Genotype transition nodes, where at least one variant has a state of one in the node and a state of zero in the parent (or vice versa).
- Genotype no-transition nodes, where all the variants have the same state (zero or one) in the parent and the node.
- Phenotype-transition nodes, where the phenotype has a state of one in the node and a state of zero in the parent (or vice versa).
- Phenotype no-transition nodes, where the phenotype has the same state (zero or one) in the parent and the node.

Note that many nodes were not assigned to any of these types due to low support or uncertain ASR results (which generated NA states). We only ran the analysis on nodes assigned to one of these types for both genotypes and phenotypes. In addition, to avoid biases from considering nodes with long branches, we discarded branches longer than 25% of the sum of all branch lengths in the tree (similar to hogwash’s approach). To calculate the association of genotype and phenotype, we considered the following two-by-two table indicating the number of nodes belonging to each type:

**Table:** 

	Genotype transition nodes (*n*_Gt_)	Genotype no-transition nodes (*n*_Gnt_)
Phenotype-transition nodes (*n*_Pt_)	*n*_Gt,Pt_	*n*_Gnt,Pt_
Phenotype no-transition nodes (*n*_Pnt_)	*n*_Gt,Pnt_	*n*_Gnt,Pnt_

For example, *n*_Gt,Pt_ indicates the number of nodes that are both genotype transition and phenotype-transition nodes. To measure the strength of the association for each group, we considered the epsilon statistic (as defined in hogwash):

$$\varepsilon =2\cdot {n}_{\mathrm{Gt,Pt}}/\left({n}_{\mathrm{Gt}}+{n}_{\mathrm{Pt}}\right)$$

This is a value between zero and one summarizing how often the transition in the phenotype is explained by a transition in the genotype as well as how often the transition in the genotype underlies a transition in the genotype. If *ε* = 1, the association is complete, meaning that there cannot be a genotype transition without a phenotype change and vice versa.

To measure the statistical significance of the association, we calculated the probability (*P*; either parametric or empirical) of having an association as strong (or stronger) as that observed by chance. To obtain parametric *P* values, we used scipy.stats (v1.5.2)^111^ to calculate the *P*_Fisher_ of each tested group. To infer empiric *P* values, we considered either ${n}_{\mathrm{Gt,Pt}}$ or the *χ*^2^ values of the two-by-two table (calculated using scipy.stats) as test statistics measuring the strength of the association. To generate a null distribution of test statistics for a given group, we generated 10,000 trees with randomly reshuffled phenotypes and real genotypes, only considering nodes with clear transition states for both genotypes and phenotypes. We then calculated, for each random sample *i*, the two-by-two association matrix and the corresponding ${\chi}_{i}^{2}$ and ${n}_{\mathrm{Gt,Pt,i}}$ statistics. We defined two empiric *P* values as

$$P({\chi}^{2})=\left(\mathop{\sum }\limits_{i=1}^{10,000}1\,\mathrm{if}\,({\chi}_{i}^{2}\ge {\chi}^{2})\right)/10,000$$

$$P({n}_{\mathrm{Gt,Pt}})=\left(\mathop{\sum }\limits_{i=1}^{10,000}1\,\mathrm{if}\,({n}_{\mathrm{Gt,Pt,i}}\ge {n}_{\mathrm{Gt,Pt}})\right)/10,000$$

To obtain each set of null phenotypes, we reshuffled the original per-strain resistance and ran ASR and phenotype state inference to define null phenotype-transition or no-transition nodes. Finally, we used the Bonferroni-corrected $P({\chi}^{2})$, $P({n}_{\mathrm{Gt,Pt}})$ or ${P}_{\mathrm{Fisher}}$ as indicators of significance (using statsmodels.stats.multitest).

A limitation of using such *P* values is that Bonferroni correction can be conservative as there is no independence between groups due to linkage between variants. This is also true for other widely used multiple-testing correction algorithms such as the FDR method used in hogwash. To address this, we calculated additional *P* values using the empiric maxT method, which has been proposed to be useful in GWAS^112,113^. Briefly, we first calculated the maximum *χ*^2^ and *ε* (across all groups) values for each random phenotype sample *i* (1,000 samples in total from the 10,000 mentioned earlier). This yielded a distribution of ${\max }{({\chi}^{2})}_{i}$ and $\max{(\varepsilon )}_{i}$ null statistics, which we used to calculate the maxT *P* values for each group as:

$$P({\chi}^{2})(\mathrm{maxT})=\left(\mathop{\sum }\limits_{i=1}^{1,000}1\,\mathrm{if}\,(\mathrm{max}{({\chi}^{2})}_{i}\ge {\chi}^{2})\right)/1,000$$

$$P(\varepsilon )({\mathrm{maxT}})=\left(\mathop{\sum }\limits_{i=1}^{1,000}1\,\mathrm{if}\,({\max }{(\varepsilon )}_{i}\ge \varepsilon )\right)/1,000$$

Note that these *P* values are already corrected for multiple testing because the null distribution of statistics considers all of the tested groups.

There are four differences between our approach to calculate *P* values and that used by hogwash. First, hogwash only uses${n}_{\mathrm{Gt,Pt}}$, which is not a statistic per se (it could be inadequate in some cases); we also considered the *X*^2^ value because it is a common statistic to measure associations from two-by-two tables. Similarly, we calculated the $P_{\mathrm{Fisher}}$ value, which is not considered in hogwash. Second, hogwash uses genotype reshuffling, which may be biased in trees with highly variable branch lengths (as we discussed in https://github.com/katiesaund/hogwash/issues/87) and motivated us to use phenotype reshuffling. Third, hogwash uses FDR correction (instead of Bonferroni) on *P* values, which may give misleading results in our dataset where there is high dependence between groups. Fourth, we calculated parametric and maxT *P* values, which are not considered in hogwash. All in all, this means that for each group, min_branch_support and ASR method, we obtained five association *P* values that may define significantly associated hits.

To maximize computational efficiency, we implemented several steps (some of them are improvements in comparison to hogwash). First, to focus on relevant groups, we only tested associations for groups with ${n}_{\mathrm{Gt,Pt}}\ge 2$, ${n}_{\mathrm{Gnt,Pnt}}\ge 1$ and an odds ratio (of the two-by-two table) of ≥1 (similar to the approach of hogwash). Second, we parallelized many steps to optimize resource consumption. Third, to avoid redundancy in variant ASR, we grouped variants into sets of fully linked variants and ran ASR for only one representative of each group. Fourth, to avoid redundancy in association tests, we merged the groups that have the same variants to only run the association test on one representative group. Fifth, to minimize the burden of *P* value inference, we first calculated empiric *P* values on 1,000 null samples, and we only used 10,000 samples if the *P* based on 1,000 samples was <0.1. All of the computational optimization steps were necessary to run the analysis on such a high number of species, drug and parameter combinations.

In summary, we applied a custom GWAS pipeline to each species and drug, resulting in an association *P* value in each group for each ASR method, min_branch_support and type of *P* value. Our approach is more comprehensive than current implementations like hogwash because we use more ASR methods, consider different types of *P* values and optimize many steps of the process. This pipeline can be used as a stand-alone software on any input dataset (see ‘Code availability’). The following sections explain how we chose the optimal parameters (ASR method, *P-*value type and min_branch_support) to define the high-confidence non-redundant set of groups underlying drug resistance.

#### Filtering GWAS results

To obtain enough power to detect associations, we only considered datasets (one for each species and drug) with at least five resistance transitions according to the ML and MP consensus ASR methods and a min_branch_support of 70. This resulted in 12 analysed species–drug pairs, comprising seven antifungal drugs (fluconazole, itraconazole, posaconazole, voriconazole, anidulafungin, micafungin and amphotericin B) in the three species (Supplementary Table 1 and Fig. 5).

To find a meaningful filtering strategy, we evaluated the significantly associated genes yielded by different parameter and filter combinations. We considered combinations of varying ASR methods (ML, MP or ML and MP), min_branch_support (50 or 70), types of *P* value (Bonferroni $P({\chi}^{2})$, Bonferroni $P({n}_{\mathrm{Gt,Pt}})$, Bonferroni $P_{\mathrm{Fisher}}$, $P({\chi}^{2})(\mathrm{maxT})$ and/or $P(\varepsilon)(\mathrm{maxT})$), minimum *ε* (0, 0.1, 0.2, 0.3, 0.4 or 0.5) and minimum ${n}_{\mathrm{Gt,Pt}}$ (two or three). For example, the most conservative parameter and filter combination would be to use the GWAS results based on the ML ASR method and a min_branch_support of 70, and define groups with *P* < 0.05 by all five types of *P* values, *ε* ≥ 0.5 and ${n}_{\mathrm{Gt,Pt}}\ge 3$ as significant. To obtain significant genes, we applied each set of parameters and filters to the raw GWAS results from both the single-variant analysis (only for non-synonymous variants) and the collapsing of non-synonymous variants at the gene and domain level. Any gene affected by significant variants or domains would also be considered as a gene yielded by the given parameter and filter combination.

We reasoned that ‘appropriate’ sets of parameters and filters should meet two criteria. First, they should yield <100 significant genes to skip overly permissive parameters. Second, appropriate parameters should minimize the false-positive burden derived from multiple testing. To test if a given parameter and filter set addressed this burden, we applied it to the single-variant GWAS results (yielding *N* significant variants) and calculated the empirical probability of having ≥*N* significant variants (*P*(*N*)) in a null dataset with random phenotypes (which lack true associations). To calculate *P*(*N*), we generated, for each species and drug combination, 50 datasets with randomly reshuffled phenotypes and then ran a per-variant GWAS analysis on each set as described in ‘Running the GWAS analysis’. For each random dataset *i*, we used the tested parameters and filters on the raw per-variant GWAS results and obtained *N*_*i*_ significant variants, which allowed us to calculate *P*(*N*) as:

$$P(N)=\left(\mathop{\sum }\limits_{i=1}^{50}1\,\mathrm{if}\,({N}_{i}\ge N)\right)/50$$

Parameters addressing the multiple-testing burden should have $P(N) < 0.05$, indicating that the observed number of significant associations is higher than what would be expected solely by random multiple testing. Note that this analysis implied a high computational cost, which is why we only used 50 re-samples. In addition, different combinations of species and drugs may require different parameters because the underlying trees and drug-resistance evolution modes may be different. After analysing this trade-off for 2,232 filter combinations, we could find appropriate parameters yielding at least one significant gene for most datasets (11/12; all except *C. glabrata*–posaconazole), suggesting that our parameter range yields meaningful GWAS hits (Supplementary Fig. 4a).

We found several appropriate filters for a given dataset (Supplementary Fig. 4a), suggesting that additional criteria were necessary to select the final optimal parameters. We reasoned that the presence of known resistance genes (*ERG11* in *C. albicans*, *ERG11* and *TAC1b* in *C. auris*, and *PDR1* in *C. glabrata* providing resistance to azoles; *FKS1* and *FKS2* in *C. glabrata*, and *FKS1* in *C. auris* providing resistance to echinocandins) among the list of significant hits could be such a criterion. To understand whether this is the case, we analysed how often the appropriate filters yielded such expected genes in 11 datasets (all except *C. auris*–amphotericin B, where we could not define expected genes). We found such expected genes in five of the datasets, but not in the other six (Supplementary Fig. 4a). To understand whether this lack of expected genes was due to limited power, we investigated whether the omission of multiple-testing considerations (*P* value corrections and *P*(*N*) constraints) would yield the expected genes (Supplementary Fig. 4c). We found that the omission of multiple-testing considerations was sufficient to yield the expected *ERG11*, *PDR1* and *TAC1b* genes in four of the six datasets (posaconazole, itraconazole and *C. albicans–*fluconazole; Supplementary Fig. 4c). This suggests that the expected genes may have mild associations to resistance but we lacked enough power to detect them without risking false positives derived from multiple testing. Conversely, none of the parameter combinations yielded the expected *FKS* genes in the other two datasets (*C. auris*–anidulafungin and *C. glabrata*–micafungin; Supplementary Fig. 4a,c), suggesting that association is probably absent in our dataset (further discussion on these datasets in Supplementary Results). In summary, expected genes may be useful to select the final filters in 5/12 datasets but not in the others due to power limitations and a lack of expected associations. We thus defined ‘potentially good’ filters as those that either yielded expected genes (in these 5/12 datasets) or that yielded some significant gene (in the remaining 7/12 datasets).

To choose the optimal parameters for each dataset, we first defined a rationally designed ‘base’ set of parameters: using the GWAS results based on the ML and MP ASR as well as a min_branch_support of 70, and defining groups with $P({\chi}^{2})(\mathrm{maxT}) < 0.05$, $P(\varepsilon )(\mathrm{maxT}) < 0.05$, *ε* ≥ 0.1 and ${n}_{\mathrm{Gt,Pt}}\ge 2$ as significant. For each dataset, we then defined the optimal set of filters as those that were potentially good for that dataset while having the least number of changes compared with the base filters (Supplementary Fig. 4b). This ensured sets of optimal parameters that were similar to one another, while adapted to each dataset, suggesting that they are useful to detect relevant associations. Note that in *C. glabrata*–posaconazole we used the base parameters because we could not find any potentially good filters, probably due to power limitations. We found that solely using maxT *P* values and filtering based on the convergence level is a suitable strategy in almost all datasets. Conversely, the choice of ASR methods and minimum branch support may be less universal, indicating that these parameters may need to be tailored to each dataset. However, the most common useful strategy is based on using a consensus between different ASR methods based on either ML or MP and requiring a minimum support of 70 (Supplementary Fig. 4b).

All in all, these were the parameters and filters used to define high-confidence GWAS results. Given that convergence-based GWAS approaches have been underused for the analysis of *Candida* pathogens, this collection of filters provides first insights into the most suitable parameters.

These parameter choices are relevant to evaluate the statistical power of our approach compared with other GWAS studies performed in fungal pathogens. For instance, previous studies in *Aspergillus fumigatus*^114^ and *C. glabrata*^22^ used *P* < 0.01 and *P* < 2.56 × 10^−7^, respectively as significance thresholds. Conversely, we mostly used *P* < 0.05 (for maxT *P* values), which at first glance may be interpreted as a sign that our filtering is overly permissive. However, note that in contrast to these previous studies, our maxT *P* values are corrected for multiple testing in a way that is tailored to each dataset (described earlier). Thus, we consider our *P* value threshold, although not directly comparable to previous studies, as sufficiently conservative to address the multiple-testing burden. Accordingly, we found that all these parameter sets yield $P(N) < 0.05$ (see above), further indicating that the chosen filtering strategies have an adequate statistical power.

#### Removing redundancy in filtered GWAS results

Given that we collapsed variants into partially overlapping groups (that is, each variant may be in several groups), these high-confidence significant hits were expected to be highly redundant. For example, if a variant is associated with resistance we expect the genes, domains and pathways related to the variant may also be significant. To remove redundancy and keep only the relevant associations, we implemented a filtering strategy to always keep the strongest and most-specific results among clusters of redundant GWAS hits. In addition, to prioritize functional associations, we only focused on protein-altering variants. The following paragraphs describe our redundancy-removal algorithm for any set of input GWAS hits.

To define a list of non-redundant hits for a set of input hits, we iterated through all of the relevant variants (those that belong to a significant group), sorted by maximum *ε* (across all groups that contain the variant) in a non-ascending way. For each variant, we identified all of the (redundant) hits that involve the variant and selected one representative non-redundant hit (the one with the strongest most-specific association). To ensure proper redundancy reduction, in each iteration we discarded (redundant) hits with variants related to some already defined non-redundant hit. To find each non-redundant hit, we hierarchically sorted the redundant hits by *ε*, odds ratio, specificity of the type of collapsing, variant type, type of mutation and number of variants related to the hit. For *ε* and odds ratio, we prioritized the largest values to keep the strongest associations. For the type of collapsing, we prioritized uncollapsed variants, followed by domains, genes, MetaCyc pathways, GO terms and finally Reactome annotations. For the type of variant, we prioritized single variant types (e.g. SVs) over combinations of types (e.g. SVs and CNVs). For the type of mutation, we prioritized more specific types (e.g. truncating) over more general ones (e.g. non-synonymous). Finally, for the number of variants, we prioritized hits with the smallest numbers of variants to increase specificity.

In some cases we found that these criteria were insufficient to get a single representative non-redundant hit, given that multiple hits had the same *ε*, odds ratio, number of variants and grouping specificity levels. In these cases we applied additional hierarchical sorting, taking into account different parameters for each type of collapsing. For gene-level collapsing, we considered the conservation across *Candida* (prioritizing genes with orthologues in the highest number of species), whether the gene had a defined name, whether the gene had orthologues in *S. cerevisiae*, the number of annotated GO terms in CGD (prioritizing the largest) and the gene length (prioritizing shorter genes). For domain-level collapsing, we considered the type of annotation (prioritizing domain-like signatures (such as Pfam or PANTHER) over biochemical-like annotations (e.g. MobiDBLite)), the range of the protein covered (prioritizing the smallest), the start of the domain (prioritizing more amino-terminal annotations), the domain annotation description lengths (prioritizing annotations with longer descriptions in cases where they cover the same protein coordinates) and the alphabetical order of the description text (in few cases where two redundant domains had a description of equal length). For Reactome collapsing, we considered the fraction of genes with a given annotation (prioritizing annotations found in fewer genes), the source species of the pathway (prioritizing *S. cerevisiae* over *S. pombe* annotations), the number of parent pathways (prioritizing those with more parents), the length of the pathway description (prioritizing longer descriptions) and the alphabetical order of the description text (as with domains). For MetaCyc collapsing, we considered the fraction of genes with a given annotation (prioritizing annotations found in fewer genes), the number of parent pathways (prioritizing those with more parents), the length of the pathway description (prioritizing longer descriptions) and the alphabetical order of the description text. For GO collapsing, we considered the fraction of genes with a given annotation (prioritizing annotations found in fewer genes), the namespace (prioritizing biological process, followed by cellular component and then molecular function), the number of children terms (prioritizing those with fewer children), the level and depth of terms (prioritizing higher values), the length of the pathway description (prioritizing longer descriptions) and the alphabetical order of the description text.

To generate the final list of high-confidence non-redundant hits (found in Supplementary Table 3), we applied this redundancy-reduction algorithm to different subsets of all significant GWAS hits. To define a set of non-redundant hits covering all involved genes, we applied the redundancy-reduction pipeline to each group of hits affecting a given gene (through either gene/domain collapsing or single-variant analysis). Next, to define non-redundant significant pathways, we first discarded significant pathways that were based on variants already considered in the significant genes. In addition, we applied the redundancy-reduction pipeline to all remaining hits grouped by each type of collapsing (Reactome, GO and MetaCyc). This generated our final list of non-redundant GWAS hits, which includes (mostly) one hit for each significant gene and also one hit for each significant non-redundant pathway that does not involve significant genes.

#### Generating a set of comprehensive low-confidence non-redundant GWAS hits

The previous sections describe how we obtained the list of high-confidence non-redundant GWAS hits analysed in the main text and shown in Fig. 6. We also generated additional sets of non-redundant GWAS hits based on more relaxed filters (low-confidence hits; Supplementary Results). We generated six such low-confidence sets, one for each combination of ASR method (ML, MP, and ML and MP) and min_branch_support (50 and 70), defining significant groups as those with an (uncorrected) $P({\chi}^{2}) < 0.05$, *ε* ≥ 0 and ${n}_{\mathrm{Gt,Pt}}\ge 2$. After applying these filters, we obtained the set of non-redundant hits using the same algorithm described in the ‘Removing redundancy in filtered GWAS results’ section. These datasets probably include some false positives and may be unsuited for exploratory analysis but they could be useful (as an example) to validate hypotheses about specific genes (where the burden of multiple testing is less prominent). In Supplementary Results we provide some examples of such hypotheses that can only be tested using the low-confidence datasets. All the low-confidence non-redundant sets of GWAS hits are provided in Supplementary Table 3.

#### Validation of high-confidence GWAS hits

To validate the GWAS high-confidence results, we tested whether we could find similar signatures of genotype–phenotype convergence in datasets published between June 2020 and June 2023. To identify such datasets, we queried PubMed to find studies related to each species and the keywords ‘genome’ and ‘susceptibility’. We then manually curated the search results to pinpoint articles providing whole-genome sequences and susceptibility measurements of clinical isolates (for each species) to the previously analysed antifungal drugs (Fig. 5a). For each genome with susceptibility information, we trimmed the reads, mapped them and called variants (small variants, SVs and CNVs) as described in the ‘Generation of the filtered variant-calling dataset for each *Candida* species’ section. In addition, we obtained a strain tree for each species and drug combination (GWAS dataset) as described in ‘Strain-tree generation’. We then ran a GWAS analysis for each of these datasets as described in the ‘Defining groups of variants for collapsed GWAS running’ and ‘Running the GWAS analysis’ sections, considering the same combinations of groupings (skipping pathway-level collapsing) and parameters but only taking into account variants affecting genes with previously defined non-redundant high-confidence hits (see ‘Removing redundancy in filtered GWAS results’). To obtain enough statistical power, we only considered datasets with at least five resistance transitions according to the consensus ML and MP ASR methods and using a min_branch_support of 70. We could find such data for five combinations of species and drugs: *C. glabrata*–fluconazole, *C. auris*–amphotericin B, *C. auris*–itraconazole, *C. auris*–posaconazole and *C. auris*–voriconazole (Extended Data Fig. 10). All of the isolates used are listed in Supplementary Table 1.

To analyse the overlap between the results of the initial GWAS and this new analysis, we identified the best ‘new hit’ related to each gene with initial high-confidence hits (query gene). For each query gene, we ranked all new hits related to that gene in a hierarchical manner by $P({\chi}^{2})$, $P({n}_{\mathrm{Gt,Pt}})$, *ε*, min_branch_support, collapsing level, mutation type, variant type and ASR method. We prioritized lower values for $P({\chi}^{2})$ and $P({n}_{\mathrm{Gt,Pt}})$, and higher values for *ε* and min_branch_support. For the type of collapsing, we prioritized uncollapsed variants, followed by domains and then genes. For the type of variant, we prioritized single variant types (e.g. SVs) over combinations of types (such as SVs and CNVs). For the type of mutation, we prioritized more specific types (that is, truncating) over more general ones (that is, non-synonymous). Finally, for the ASR method we prioritized ML-, followed by MP- and then ML and MP-related hits. This ranking enabled us to define one new best hit (at the top of this ranking) for each query gene, which is necessary to compare the results of both GWAS analyses (Supplementary Results and Extended Data Fig. 10). Note that for some genes we could not find any such genes, as there were <2 nodes with convergence (both genotype and phenotype transitions).

### Reporting summary

Further information on research design is available in the Nature Portfolio Reporting Summary linked to this article.

### Supplementary information

Supplementary Information Supplementary results and discussion, Supplementary Figs. 1–4 and Supplementary Tables 1–3.

Reporting Summary

Supplementary Table 1 Strains used in this study.

Supplementary Table 2 Recent selection data.

Supplementary Table 3 GWAS associations.

## Data Availability

All of the NCBI SRA sequencing datasets analysed are in Supplementary Table 1. In addition, the variants are available at https://candidamine.org. Furthermore, the GitHub repository https://github.com/Gabaldonlab/Candida_Selection_DrugResistance contains the comma-separated-value (CSV) versions of the supplementary tables.

## Code availability

All of the code and software environments used to generate the datasets, results, tables and figures presented here are in https://github.com/Gabaldonlab/Candida_Selection_DrugResistance. Note that this GitHub repository contains the convergence-based GWAS and the tree-generation stand-alone pipelines, which may be useful beyond this project.
