# Supplementary material for: Recent gene selection and drug resistance underscore clinical adaptation across Candida species
Source: Nat Microbiol. 2024 Jan 4;9(1):284–307. doi: 10.1038/s41564-023-01547-z (PMC10769879; doi:10.1038/s41564-023-01547-z)
Supplement: Supplementary file 1 — Supplementary results and discussion, Supplementary Figs. 1–4 and Supplementary Tables 1–3. [file 41564_2023_1547_MOESM1_ESM.pdf]

nature microbiology

Analysis

https://doi.org/10.1038/s41564-023-01547-z

Recent gene selection and drug resistance underscore clinical adaptation across *Candida* species

In the format provided by the authors and unedited

This is the supplementary material to the paper 'Recent gene selection and drug resistance underscore clinical adaptation across *Candida* species'. This document includes Supplementary Results and Discussion, Supplementary Figures and Supplementary Table legends. Below is the content of this file:

SUPPLEMENTARY RESULTS AND DISCUSSION

The results shown in this section complement some of the main findings of this work, described in **Results and Discussion**.

Evolutionary interactions between paralogs in recent selection

To detect complex evolutionary recent selective interactions between in-paralogs of the same gene family we evaluated, for each species, the overlap between orthologous groups (OGs) affected by selection in different variant types (nsyn_SNPs, if_INDELs, DELs and DUPs) (**Figure 3B**). We find that most OGs are affected by a single variant type, as only 29/879 OGs have a gene affected by selection on different types. However, the overlap between OGs shaped by selection on nsyn_SNPs or DELs was higher than expected by chance ($p<0.05$, see **Online Methods**) in three species (**Figure 3B, Supplementary Table 2**), suggesting that these variants may yield complex evolutionary outcomes in some families. For example, DELs are selected in adhesins *EPA6*, *EPA7* and *EPA2* in *C. glabrata*, while nsyn_SNPs are selected in *EPA1* and *EPA15*. Similarly, some paralogs from the *FGR51* group in *C. albicans* (involved in filamentous growth) have selected DELs, while others have selected nysn_SNPs. This could be explained by an antagonistic effect among family members, where some paralogs are intrinsically more adaptable (and thus acquire gain of function (GoF) nsyn_SNPs), while the others tend to be deleted. In addition, both DELs and nsyn_SNPs were selected in *MRR1a* (a MDR gene) from *C. auris*, *MIT1* (related to pseudohyphal growth), *IRA1* (encoding a GTPase-activating protein) and *GPR1* (related to invasive growth) from *C. glabrata* and *RIO2* (involved in caspofungin sensitivity) from *C. albicans*. These could be instances where loss of function (LoF) nsyn_SNPs were selected, which may be equivalent to DELs. Overall, these results suggest that gene loss can be a major driver of recent adaptation.

Validation of the clinical relevance of recent selection signatures

The signatures of recent selection inferred in this work (see **Results and Discussion**, **Figure 3**) reflect clinical adaptation under the assumption that recently-appeared variants (in monophyletic groups comprising only closely related clinical isolates) are highly enriched in those acquired in a clinical context. This is an important assumption that may not be always met, as some of the variants appearing within pairs of close clinical strains may be unrelated to the clinics. For example, two isolates found in different patients, even if they are evolutionary close, may have evolved for a short period of time in the environment, so that some of their variants may be related to environmental adaptation. To test our assumption and further validate

our method we took advantage of the fact that several of these close strains are clonal serial isolates of the same patient. We reasoned that selective signatures identified among pairs of such serial isolates are most likely clinically-related, as variants differing across such strains appeared within the human host. Thus, if our assumption of clinical relevance is valid, we should expect a significant overlap when comparing the signs of selection in such serial isolates and those inferred by our recent selection-detection method (**Figure 3A**).

To identify genes possibly under selection in serial clonal isolates we mined the literature associated with our datasets to find strains that were sequential serial isolates of a given patient. We could find >5 pairs of such serial isolates in our dataset for *C. albicans* (37 pairs), *C. auris* (23 pairs) and *C. glabrata* (6 pairs) (**Supplementary Table 1**). Since some of these pairs may include divergent strains co-infecting a given patient, we only considered pairs of strains where the latter isolate had acquired <1SNP/kb. Thus, we analyzed 31 pairs for *C. albicans*, 23 pairs for *C. auris* and 6 pairs for *C. glabrata*. We identified as 'genes with signs of clinical selection' in these serial isolates as those that acquired a nsyn_SNPs, if_INDEL, DEL and/or DUP variant in the latter isolate in at least two pairs. This yielded between 1 - 1505 such genes, depending on the species and type of variant (12 combinations, as there are three species and four variant types).

To test whether genes under recent selection (**Figure 3**) are enriched in those implicated in clinical adaptation, we analyzed the overlap between such genes and those recurrently mutated in serial isolates. Consistent with our initial hypothesis, we find that in most species - type variant combinations (11/12, all except C. glabrata - nsyn_SNPs) there are several overlapping (between 1-292) such overlapping genes. To validate that such overlaps are not the result of randomly comparing two sets of genes, we performed a Fisher test for each combination. In most combinations with overlapping genes (10/11, all except C. auris - nsyn_SNPs) we find that the probability of observing such overlaps by chance is very low ($p<0.05$, see Online Methods), further supporting our approach.

In summary, the fact that we find statistically significant overlaps between these genes supports our approach of measuring clinically-relevant signatures of adaptation by finding genes affected by recent selection.

Recent-selection has been unique for each *Candida* species

Our analyses revealed that most of the pathways enriched in genes under selection are not shared across taxa, suggesting mostly species-specific mechanisms of adaptation (see **Results and Discussion**, **Figure 4**). In *C. glabrata*, these species-specific processes include response to oxygen-containing compounds, regulation of filamentous growth, regulation of actin nucleation, pigment catabolism, tRNA methylation, bud-site selection, G-protein signaling and nuclear membrane proteins. In *C. auris*, rRNA binding, the TTT complex (regulating the phosphatidylinositol-3-related kinase) and the host cellular component. In *C. tropicalis*,

flocculation, regulation of transcription (related to the histone deacetylase complex), oligosaccharide synthesis, glucan degradation and protein mannosylation. In *C. albicans*, response to temperature stimulus, inositol phosphate dephosphorylation, cell wall and extracellular region. Finally, in *C. orthopsilosis* we find carbohydrate metabolism (including hexose transport) functions.

These processes may be used to infer mechanisms of host adaptation and/or drug resistance that are particularly important in each of the *Candida* pathogens. Thus, in the paragraphs below we propose speculative scenarios explaining why changes in these species-specific functions could be adaptive (**Figure 4**). For instance, in *C. glabrata* the changes in filamentous growth could reflect a modulation of pseudohyphae formation, which may promote virulence by increasing macrophage escape and host invasion, as previously proposed[1,2]. In addition, the duplications in tRNA methylation genes may be adaptive because they provide general stress tolerance, given that upregulation of such genes has been observed as a response to various stresses in this species (i.e. cell wall, heat and oxidative stresses, faced in the host)[3]. Conversely, the changes in actin nucleation in this species could reflect adaptations to azoles, as these drugs induce actin cytoskeleton remodeling[4]. Similarly, the alterations in bud site selection, which is also related to the actin cytoskeleton[5], could also reflect such antifungal adaptation. In addition, the changes in (cAMP-related) G protein-coupled receptor signaling may also represent adaptations to azoles, given that cAMP-related pathways articulate the physiological response to this antifungal[6]. In summary, we propose various host and drug adaptation mechanisms that are unique in *C. glabrata*, including the changes in i) pseudohyphae formation, ii) general stress responses, iii) actin cytoskeleton and iv) cAMP signaling. Such a large number of distinct functions are consistent with the large distance between this species and the other CTG-clade *Candida* species[7].

However, we also find relevant species-specific functions under selection within the CTG taxa, suggesting that some of the important host adaptation / drug resistance mechanisms also differ between these closer species. For instance, in *C. auris* the changes in rRNA binding proteins may be an adaptation to echinocandins, as the transcriptomic response to caspofungin involves upregulation of ribosomal genes[8]. In addition, the changes in the TTT complex may reflect the importance of phosphatidylinositol 3-kinase signaling for adhesion and filamentation in this species, as previously proposed in *C. albicans*[9]. Furthermore, the selective processes affecting genes from the host cellular component in *C. auris* may be related to the lower ability of phagocytic cells to engulf and kill this pathogen, as compared to *C. albicans*[10]. Conversely, in *C. albicans*, the changes in temperature responses may enhance host survival and virulence during invasive infections that trigger fever[11]. Conversely, the changes in inositol phosphate dephosphorylation may be related to changes in virulence through altered filamentation, as inositol phosphate changes are related to this process[12]. Finally, the changes in the cell wall could be related to adaptation to cell wall stressors, such as echinocandins[13].

On another line, in *C. tropicalis* the changes in histone deacetylases (HDACs) are consistent with the role of these proteins in modulating virulence, biofilm formation and host dissemination in *Candida* species[14]. In addition, changes in oligosaccharide synthesis, glucan degradation and protein mannosylation may reflect the importance of protein glycosylation for virulence, cell wall integrity, interactions with the immune system and/or hyphal growth[15]. Finally, the changes in carbohydrate metabolism and transport in *C. orthopsilosis* may reflect the importance of energy obtention processes and/or sugar-related signaling[16] in this species. Taken together, these speculative scenarios suggest multiple host and drug adaptation mechanisms that are unique in *C. auris, C. albicans, C. tropicalis* or *C.orthopsilosis*. These involve changes in i) ribosomal regulation, ii) inositol signaling, iii) interaction with phagocytes, iv) temperature responses, v) cell wall integrity, vi) HDAC-mediated regulation, vii) protein glycosylation and viii) carbohydrate metabolism. Such differences in recent adaptation reveal the unique selective constraints of each *Candida* pathogen, which hints to relevant species-specific cell functions and/or therapeutic targets. This discussion, although highly speculative, opens many interesting research questions that may be pursued through further experimentation.

Performing convergence-based GWAS in *Candida* species

To understand how to best do GWAS in our datasets we evaluated the types of groupings (in terms of types of variants and various collapsing levels) that yield significant groups (**Figure 6**). We performed one 'collapsed' GWAS for different combinations of 'variant type' (SVs, CNVs and/or small variants), 'mutation type' (non-synonymous and/or truncating) and 'functional level' (domains, genes or pathways (GO, Reactome, MetaCyc)) (**Online Methods**, **Figure 6**). For example, in one of these GWAS we tested the genotype-phenotype association for each gene (functional level), considering truncating (mutation type) small variants and SVs (variant type). In doing so, we gained several novel insights about how different GWAS strategies work. First, we often find stronger associations if we include SVs and CNVs in addition to small variants (55% of hits consider SVs/CNVs), suggesting that such complex variants partially underlie resistance changes. Second, in some tests we find stronger associations if we consider only truncating variants (24% of hits consider only truncations), indicating that gene truncation is a major driver of drug resistance. Third, collapsing variants at the gene, domain and/or pathway level yields most of our significant results (60.4% of hits involve domains, 14.5% involve genes, 17.2% involve pathways and 7.9% involve no collapsing). This suggests that collapsing is essential to detect convergence that would be missed if only testing single variants (**Figure 6**). Of note, pathway collapsing can be essential to find associations in datasets with insufficient strains for typical variant-focused or gene-based collapsing (i.e. *C. glabrata* posaconazole) (**Figure 6**). Similarly, domain collapsing is key to find regions of proteins underlying resistance (**Figure 6**).

Recombination partly underlies the emergence of drug resistance

To understand the landscape of the genotype-phenotype associations from our GWAS we evaluated the Manhattan plots that show the correlation of each variant with the drug resistance phenotype (**Extended Data Figure 5,6**). We find that there are significant variants in some datasets (1 in *C. albicans*/fluconazole, 65 in *C. glabrata*-fluconazole, 52 in *C. glabrata*/micafungin, 6 in *C.auris*/amphotericin B, 43 in *C.auris*/fluconazole, 66 in *C.auris*-voriconazole), distributed across the genome. Each of these single-variant GWAS hits may be explained by either i) *de novo* acquisition of the same variant in independent resistant lineages or ii) mating of susceptible and resistant isolates, propagating the resistance variant into the resulting offspring through (para)sexual recombination. The fact that there are often >10 such variants distributed across the genome suggests that recent genome-wide recombination explains most of these hits. To further validate this role of recombination we checked whether significant SNPs are linked to each other. Accordingly, 83.3%-100% of these SNPs (depending on the dataset) are linked to at least one other significant SNPs (two SNPs are considered linked if they are together and may underlie resistance transitions in >1 strain). In addition, visual examination of the SNPs in the tree (**Extended Data Figure 7**) suggests that there are linked haplotypes of significant SNPs. Note that we focused on SNPs because they are the most reliable variants for such linkage analysis. As mentioned in the main text, these results support the idea that recombination has played a role in the emergence of drug resistance. However, we cannot fully discard that some of the single-variant GWAS hits come from independent *de novo* acquisitions. For instance, the few variants that are not linked to other variants associated with resistance may be unrelated to recombination events.

Analysis of known drivers of drug resistance

Finally, to validate our GWAS filtering strategy and gain insights into known mechanisms of antifungal drug resistance we inspected GWAS results for genes that are known drivers of resistance (see **Supplementary Table 3**). Variants in *ERG11* (the target of azoles[17]) are associated to resistance in *C. auris* fluconazole (non-synonymous small variants in the PTHR24286 PANTHER signature) and *C. auris* voriconazole (non-synonymous small variants in the protein region 101-150). In addition, variants in *C. glabrata*'s *PDR1* (a transcription factor (TF) driving expression of the *CDR1* efflux pumps[18]) are associated to fluconazole resistance (non-synonymous small variants and CNVs (including truncations) in the protein region 901-1000) and voriconazole resistance (non-synonymous small variants). Similarly, variants in *TAC1b* (a TF driving expression of azole efflux pumps[19]) are associated with voriconazole resistance in *C. auris* (non-synonymous small variants in the protein region 211-235). Finally, variants in *FKS1* (the target of echinocandins) are associated to micafungin resistance in *C. auris* (non-synonymous small variants in the protein region 580-679 in gene *B9J08_000964*, which includes a 'hotspot' region that has been previously associated to

resistance[20,21]). These findings suggest that our GWAS pipeline yields relevant results and confirm these genes as important drivers of resistance.

However, we missed expected genes in some datasets: *ERG11* in *C. albicans* fluconazole and *C. auris* itraconazole/posaconazole, *PDR1* in *C. glabrata* posaconazole, *TAC1b* in *C.auris* fluconazole/itraconazole/posaconazole and *FKS* genes in *C. glabrata* micafungin and *C. auris* anidulafungin. Since our filtering strategy was conservative to limit false positives (see **Online Methods**), we hypothesize that the lack of such genes may result from limited statistical power in some datasets impeding the finding of all true associations. To test this and to better dissect these lack of associations we evaluated whether different sets of relaxed filters would yield the significant genes (**Online Methods** and **Supplementary Figure 4C**), and we also checked the corresponding set of low-confidence GWAS hits (**Supplementary Table 3**). We find that this is sometimes true, since some filters yielded the previously missed *ERG11* in *C. albicans* fluconazole, *PDR1* in *C. glabrata* posaconazole and *TAC1b* in *C.auris* fluconazole/itraconazole/posaconazole. This suggests that the lack of high-confidence associations around the expected genes is sometimes related to limited statistical power, but that these genes actually play a role in resistance. However, we could not find relaxed filters yielding significant hits involving *ERG11* in *C. auris* itraconazole/posaconazole nor *FKS* genes in *C. glabrata* micafungin and *C. auris* anidulafungin, suggesting a true lack of association. All in all, these results suggest that *ERG11* is key in *C. albicans* fluconazole and *C. auris* fluconazole / voriconazole resistance, *TAC1b* drives pan-azole resistance in *C. auris*, *FKS* mutations are drivers of micafungin resistance in *C. auris* and *PDR1* underlies pan-azole resistance in *C. glabrata*. Conversely, *ERG11* may be unrelated to resistance towards some azoles in *C. auris*, while *FKS* mutations could be less important in our *C. glabrata* micafungin and *C. auris* anidulafungin datasets. Although most of these results are confirmatory, we consider them relevant because i) they validate previously-established mechanisms with a statistically powerful approach and ii) they overly validate our GWAS strategy.

The lack of correlation between *FKS* mutations and resistance in *C. glabrata*/micafungin and *C. auris*/anidulafungin was puzzling because there is strong evidence that this is the major driver of echinocandin resistance[20]. To further understand this observation we visualized the trees and the actual MICs (**Extended Data Figure 9**). In both species/drug datasets there is a mix between highly-resistant strains (MICs of 0.5-1 in *C. glabrata*/micafungin and 8-16 in *C. auris*/anidulafungin) and (more prevalent) intermediately-resistant strains (MICs of 0.06-0.1 in *C.glabrata*/micafungin and 2-4 in *C. auris*/anidulafungin) (**Extended Data Figure 9**). As expected, the highly resistant samples often have canonical hotspot *FKS* mutations, but there are not enough such samples (only 1 in each drug) to drive a significant association. However, the intermediately-resistant samples lack *FKS* mutations, suggesting that some degree of echinocandin resistance can exist without these mutations. We can find other variants and groups correlated to echinocandin resistance in these drugs (13 hits in *C. glabrata* micafungin and 4 in *C.*

auris anidulafungin), which may be driving the resistance phenotype (**Supplementary Table 3**). For *C. glabrata*/micafungin the top hit is a G993S variant in the putative rRNA regulator *CAGL0H02783g* (ortholog of *NET1* in *S. cerevisiae*) (**Extended Data Figure 9A**). For *C. auris*/anidulafungin the top hit are variants (the most important is a SV) around the putative filamentation-related glycoprotein *B9J08_003526* (ortholog of *MUC1* in *C. albicans*) (**Extended Data Figure 9B**). These examples suggest that non-*FKS* functions like filamentous growth or ribosomal function could be important for intermediate echinocandin resistance. In addition, this illustrates a common limitation of GWAS analyses: resistance phenotypes are not 100% dichotomous which may complicate the interpretation of results. In addition, this example shows how, despite only considering strains with strong resistance/susceptibility, the strains with 'strong resistance' (in the context of the whole MIC distribution (**Supplementary Figure 3**)) could be further stratified in various levels of resistance, each entailing diverse underlying evolutionary mechanisms.

Beyond confirming previously known drivers, which underscores the validity of our approach, our analyses of azole resistance driver genes in *C. auris* provide some rather novel findings. Despite an overall similarity of mechanisms across different azoles, there was one relevant unexpected exception: *ERG11* variants were related to resistance towards fluconazole and voriconazole in *C. auris*, but they appear to be unrelated to itraconazole or posaconazole resistance. This is consistent with previous studies re-introducing *C. auris* *ERG11* variants in susceptible strains showed a strong impact on fluconazole / voriconazole resistance, but a minimal or nonexistent effect on itraconazole / posaconazole susceptibility[22,23]. From a chemical structure point of view, this could be due to the fact that fluconazole and voriconazole are short-tailed azoles, while itraconazole and posaconazole are long-tailed compounds[22,24]. Thus, our findings indicate that the role of *ERG11* variants in azole resistance is limited to some, perhaps only short-tailed, azoles in *C. auris*. These results underscore the need of studying resistance towards different drugs, even if they have a presumably similar mechanism of action.

Validation of high-confidence GWAS hits

To validate the GWAS high-confidence results we tested whether we could find similar signatures of genotype-phenotype convergence in datasets published between June 2020 and June 2023, which were not used in the analysis (see **Online Methods**). This served as an independent validation of our results, which is important given the exploratory nature of our approach. We could find sufficient data for five combinations of species and drugs (having at least five sharp resistance transitions): *C. glabrata* - fluconazole, *C. auris* - amphotericin B, *C. auris* - itraconazole, *C. auris* - posaconazole and *C. auris* - voriconazole (**Extended Data Figure 10**). For each gene with a high-confidence hit in the GWAS (**Supplementary Table 3**), we ran a genotype-phenotype association testing using various parameters and types of groupings equivalent to those used in the GWAS analysis. We thus obtained, for each gene with high confidence hits in a GWAS dataset (hereafter referred as 'GWAS hit') a 'new hit', which refers to the testing group in the new dataset

related to that gene that yielded the lowest association p values and highest convergence level (ε). Note that we only considered as 'new hits' those genes where some association testing yielded a sign of convergence (at least two nodes with genotype and phenotype transitions). We then compared the 'GWAS hits' and 'new hits' to understand the reliability of the high confidence GWAS results (see **Online Methods** and **Extended Data Figure 10**).

Out of 78 genes with 'GWAS hits' we found 52 genes (66.67%) with matching 'new hits', suggesting that most of the high-confidence GWAS hits show an equivalent degree of convergence in the new dataset. To provide a statistical framework to this analysis we modified the definition of 'new hits' to only include those with a low association $p(X^2)$ ($p<0.1$ or $p<0.05$, see **Extended Data Figure 10**). Applying these p value thresholds reduced the number of overlapping genes, as only 38/78 (48.72%) and 28/78 (35.90%) yielded 'new hits' (for $p<0.1$ and $p<0.05$, respectively). These findings further confirm the association with resistance for at least 35.90 - 66.67% of the genes with high-confidence GWAS hits. To validate the genes belonging to OGs with hits in multiple GWAS datasets (considered as the most relevant findings of our analysis, see **Results and Discussion**) we performed a similar comparison but only on such genes (22 in total). We find 18/22 genes (81.82%) with 'new hits', and applying stringent p value thresholds yields 14/22 (63.64%) and 10/22 (45.45%) genes (for $p<0.1$ and $p<0.05$, respectively) (**Extended Data Figure 10**). These findings confirm the association with resistance for most of these genes (45.45% - 81.82%) that were found in multiple GWAS datasets. Note that, even if all the hits from the GWAS came from true associations we would not expect a 100% overlap in this analysis, as the new datasets had mostly smaller sample sizes and limited statistical power (**Extended Data Figure 10**). Thus, we consider that the high overlaps provide support to our GWAS approach.

In summary, we performed an independent validation of our high-confidence GWAS results, which further confirms most of the genes implicated in >1 dataset. Most importantly, these validated genes include newly identified resistance players, such as *AWP4*, *PWP4*, *NET1* and *NRG1* in *C. glabrata* - fluconazole and *MRPS35* in *C. auris*' azoles (**Extended Data Figure 10**). Further experimental validation will be key to fully confirm the role of these genes in antifungal drug resistance.

Analysis of low-confidence GWAS hits can be useful to test specific hypotheses about drug resistance genes

In the main text and **Figure 6** we describe the high-confidence non-redundant (NR) GWAS hits, which provide relevant exploratory insights. However, since our filtering strategy was rather conservative to minimize false positives, it can miss true associations due to limited statistical power, as shown, for instance, by the absence of high-confidence *ERG11* hits in *C. albicans* fluconazole (see above). One of the aims of this

work is to provide a comprehensive GWAS dataset useful to validate the clinical importance drug resistance mechanisms inferred in other studies (i.e. *in vitro* evolution approaches). This motivated us to also provide additional sets of NR GWAS hits based on more relaxed filters (low-confidence hits), obtained as described in **Online Methods**, and available at **Supplementary Table 3**. As an example, these datasets could be useful to validate hypotheses about specific genes (where the burden of multiple testing is less prominent).

To illustrate this we tested the clinical validity of the findings reported in a recent *in vitro*-evolution study on *C. glabrata*, which suggested that chromosome E duplications and mutations in *ERG11*, *PDR1*, *CDR1*, *CNE1*, *EPA13*, *FKS1*, *FKS2*, *ERG3*, *ERG4* are related to fluconazole and anidulafungin resistance[25]. As mentioned above, *PDR1* mutations are correlated to all azoles tested in *C. glabrata*. *CDR1* had low-confidence hits (non-truncating small variants and CNVs) in voriconazole (region 401-500 of the protein) and fluconazole (ABC transporter signature in the protein region 165-325). *CNE1* had low-confidence hits in fluconazole, involving non-synonymous small variants (some truncating) in protein region 380-383. *EPA13* had low-confidence hits involving various protein regions, small variants and CNVs (some truncating) in fluconazole and posaconazole. These hits suggest that *PDR1, CDR1, CNE1* and *EPA13* could be important for clinical azole resistance, as predicted from the *in vitro* experiment. However, we could not find any hits involving *ERG11* nor chromosome E (containing *ERG11*) duplications, suggesting that this gene may not be related to azole resistance in the clinics (as previously reported[21,26]). This lack of expected *ERG11* hits motivated us to understand whether mutations in other genes of the ergosterol biosynthetic pathway could have an analogous role to the *in vitro* effects of *ERG11* changes. Accordingly, the GO term 'ergosterol biosynthetic process' is a high-confidence hit in voriconazole. In addition, we find low-confidence hits affecting *UPC2A* (a transcription factor regulating azole resistance), *ERG4* and *ERG13* (enzymes implicated in ergosterol biosynthesis, regulated by *UPC2A*[27]) in fluconazole, posaconazole and/or voriconazole. Based on these results, we speculate that, while *ERG11* may be unrelated to clinical azole resistance, these other members of the ergosterol biosynthesis pathway do, resulting in similar outcomes (i.e. higher ergosterol production which compensates azole inhibition). A possible explanation for this difference is that *ERG11* mutations may yield a higher fitness cost during human infection as compared to mutations in other members of ergosterol biosynthesis. This could explain why such mutations drive resistance *in vitro*, but not in clinical isolates. On another note, we could not find echinocandin-related hits involving *ERG3*, *ERG4, FKS1* or *FKS2*, likely because our *C. glabrata* micafungin dataset does not include enough strains with a strong resistance (mentioned above).

All in all, by using our GWAS dataset we could validate the clinical relevance of antifungal drug resistance mechanisms inferred from *in vitro* studies in *C. glabrata*. Beyond this example, our dataset will be useful to validate future findings in other species and drugs.

SUPPLEMENTARY FIGURES

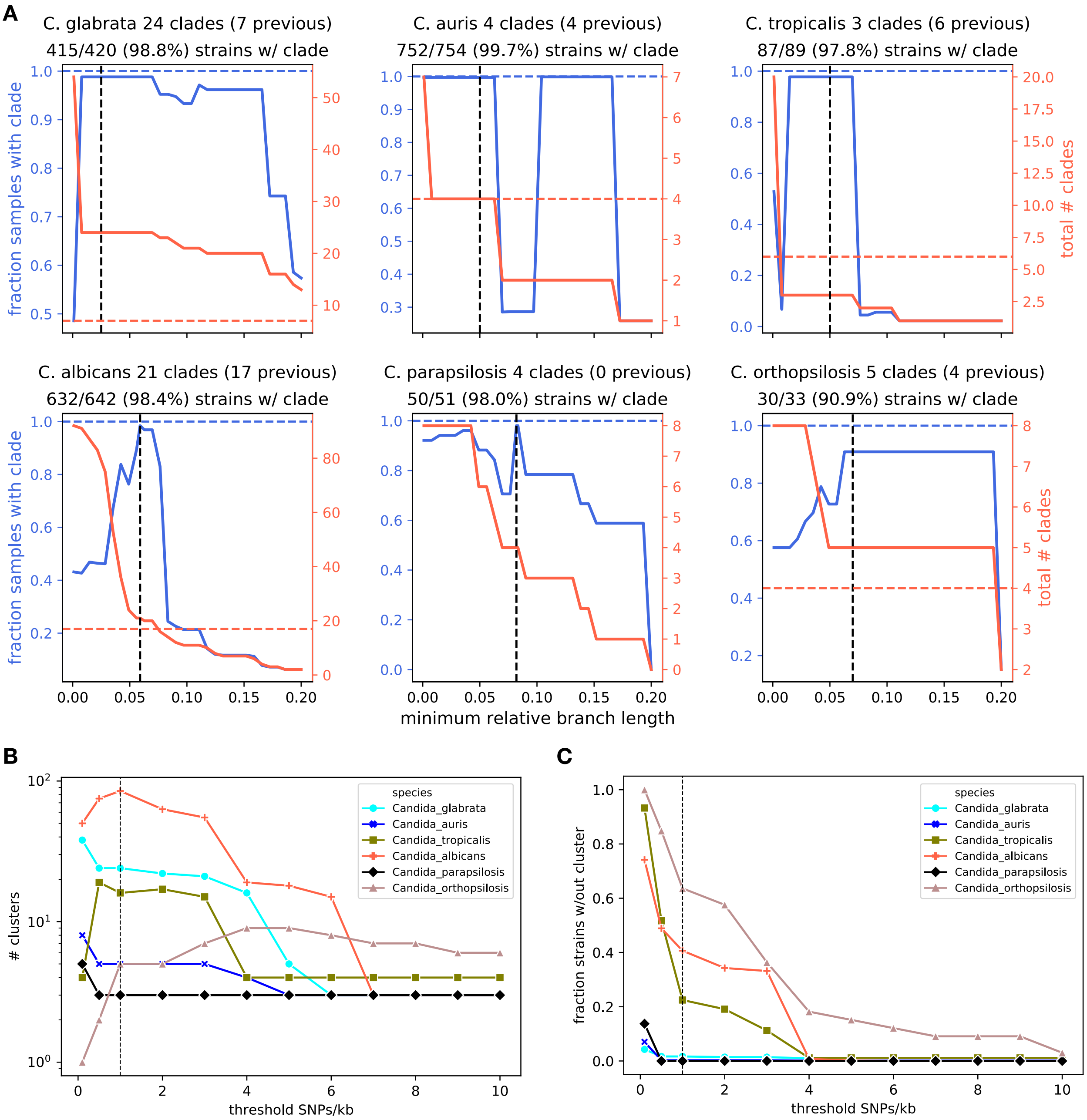

A
C. glabrata 24 clades (7 previous)
415/420 (98.8%) strains w/ clade
C. auris 4 clades (4 previous)
752/754 (99.7%) strains w/ clade
C. tropicalis 3 clades (6 previous)
87/89 (97.8%) strains w/ clade
C. albicans 21 clades (17 previous)
632/642 (98.4%) strains w/ clade
C. parapsilosis 4 clades (0 previous)
50/51 (98.0%) strains w/ clade
C. orthopsilosis 5 clades (4 previous)
30/33 (90.9%) strains w/ clade
fraction samples with clade
total # clades
minimum relative branch length
B
clusters
threshold SNPs/kb
C
fraction strains w/out cluster
threshold SNPs/kb
species
Candida_glabrata
Candida_auris
Candida_tropicalis
Candida_albicans
Candida_parapsilosis
Candida_orthopsilosis

Supplementary Figure 1. Defining clades and clusters of strains in a systematic manner. (A) To define clades in a systematic manner we had to choose a minimum branch length threshold (see **Online Methods**). These plots show the relationship between the minimum branch length that defines a clade (see **Online Methods** and **Figure 1B**) and the fraction of strains assigned to some clade (blue) or the total number of clades (red). To define the final set of clades, we set a minimum branch length threshold that maximized the number of strains with a clade and minimized the total number of clades (dashed vertical lines). The title of each subplot indicates the number of clades resulting from the selected thresholds, the number of clades defined in previous studies (also in the red dashed line) and the fraction of strains within some clade. **(B,C)** To define clusters of clonal clinical strains in the analysis of selection (**Online Methods**) we had to define a threshold for the maximum number of SNPs/kb allowed between two strains of a given cluster. These plots show the relationship between different thresholds and the number of clusters (B) or the fraction of strains that can be assigned to a cluster (C). We chose 1 SNP/kb (black dashed line) as a reasonable value because most strains were into some cluster without a very high divergence threshold. We added a pseudocount of 1 cluster to all points of (B) to show log scales.

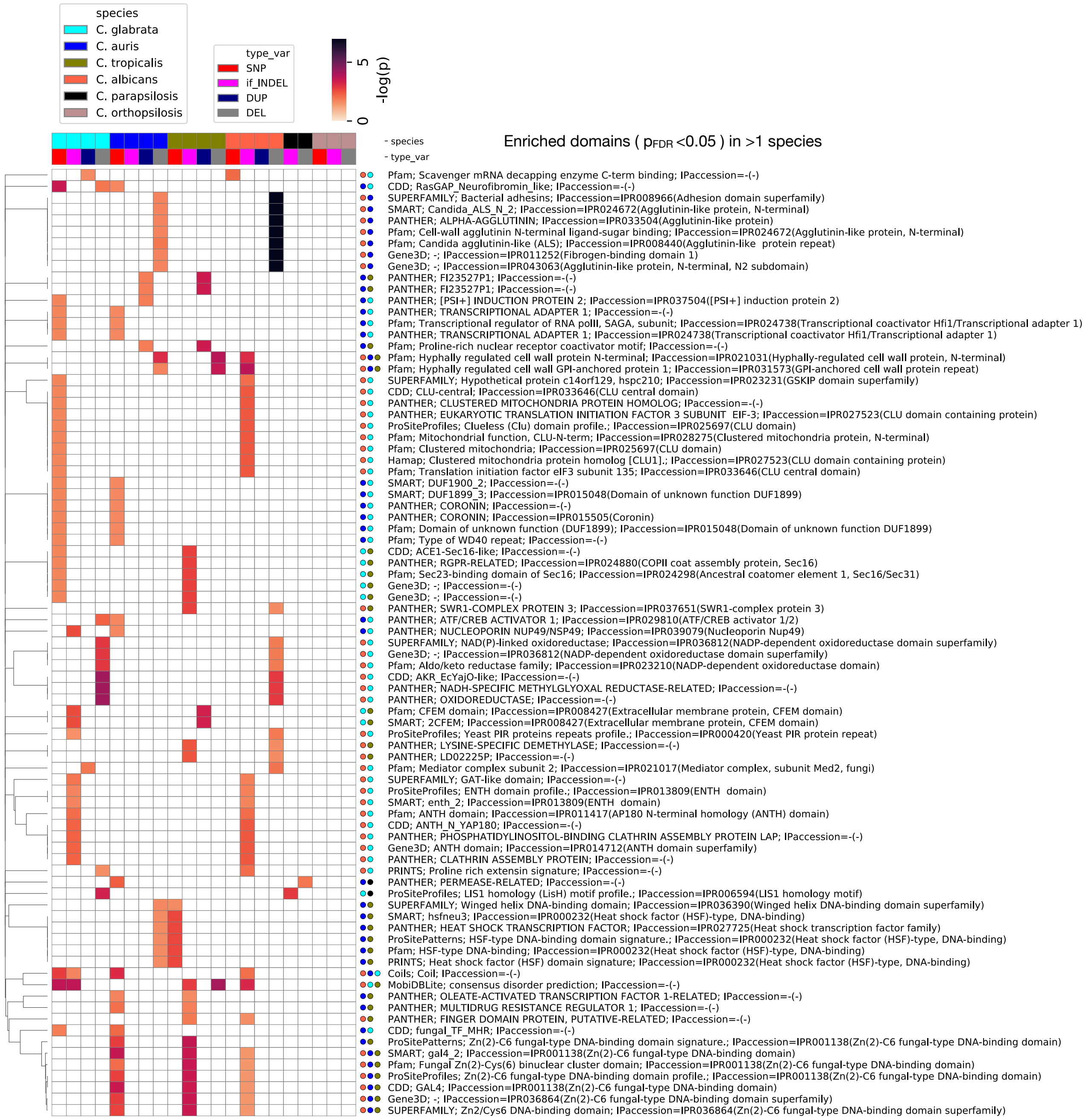
species
C. glabrata
C. auris
C. tropicalis
C. albicans
C. parapsilosis
C. orthopsilosis
type_var
SNP
if_INDEL
DUP
DEL
-log(p)
0
5
species
type_var
Enriched domains (p_{FDR} <0.05) in >1 species
Pfam; Scavenger mRNA decapping enzyme C-term binding; IPaccession=-(-)
CDD; RasGAP_Neurofibromin_like; IPaccession=-(-)
SUPERFAMILY; Bacterial adhesins; IPaccession=IPR008966(Adhesion domain superfamily)
SMART; Candida_ALS_N_2; IPaccession=IPR024672(Agglutinin-like protein, N-terminal)
PANTHER; ALPHA-AGGLUTININ; IPaccession=IPR033504(Agglutinin-like protein)
Pfam; Cell-wall agglutinin N-terminal ligand-sugar binding; IPaccession=IPR024672(Agglutinin-like protein, N-terminal)
Pfam; Candida agglutinin-like (ALS); IPaccession=IPR008440(Agglutinin-like protein repeat)
Gene3D; -; IPaccession=IPR011252(Fibrogen-binding domain 1)
Gene3D; -; IPaccession=IPR043063(Agglutinin-like protein, N-terminal, N2 subdomain)
PANTHER; FI23527P1; IPaccession=-(-)
PANTHER; FI23527P1; IPaccession=-(-)
PANTHER; [PSI+] INDUCTION PROTEIN 2; IPaccession=IPR037504([PSI+] induction protein 2)
PANTHER; TRANSCRIPTIONAL ADAPTER 1; IPaccession=-(-)
Pfam; Transcriptional regulator of RNA polII, SAGA, subunit; IPaccession=IPR024738(Transcriptional coactivator Hfi1/Transcriptional adapter 1)
PANTHER; TRANSCRIPTIONAL ADAPTER 1; IPaccession=IPR024738(Transcriptional coactivator Hfi1/Transcriptional adapter 1)
Pfam; Proline-rich nuclear receptor coactivator motif; IPaccession=-(-)
Pfam; Hyphally regulated cell wall protein N-terminal; IPaccession=IPR021031(Hyphally-regulated cell wall protein, N-terminal)
Pfam; Hyphally regulated cell wall GPI-anchored protein 1; IPaccession=IPR031573(GPI-anchored cell wall protein repeat)
SUPERFAMILY; Hypothetical protein c14orf129, hspc210; IPaccession=IPR023231(GSKIP domain superfamily)
CDD; CLU-central; IPaccession=IPR033646(CLU central domain)
PANTHER; CLUSTERED MITOCHONDRIA PROTEIN HOMOLOG; IPaccession=-(-)
PANTHER; EUKARYOTIC TRANSLATION INITIATION FACTOR 3 SUBUNIT EIF-3; IPaccession=IPR027523(CLU domain containing protein)
ProSiteProfiles; Clueless (Clu) domain profile.; IPaccession=IPR025697(CLU domain)
Pfam; Mitochondrial function, CLU-N-term; IPaccession=IPR028275(Clustered mitochondria protein, N-terminal)
Pfam; Clustered mitochondria; IPaccession=IPR025697(CLU domain)
Hamap; Clustered mitochondria protein homolog [CLU1].; IPaccession=IPR027523(CLU domain containing protein)
Pfam; Translation initiation factor eIF3 subunit 135; IPaccession=IPR033646(CLU central domain)
SMART; DUF1900_2; IPaccession=-(-)
SMART; DUF1899_3; IPaccession=IPR015048(Domain of unknown function DUF1899)
PANTHER; CORONIN; IPaccession=-(-)
PANTHER; CORONIN; IPaccession=IPR015505(Coronin)
Pfam; Domain of unknown function (DUF1899); IPaccession=IPR015048(Domain of unknown function DUF1899)
Pfam; Type of WD40 repeat; IPaccession=-(-)
CDD; ACE1-Sec16-like; IPaccession=-(-)
PANTHER; RGPR-RELATED; IPaccession=IPR024880(COPII coat assembly protein, Sec16)
Pfam; Sec23-binding domain of Sec16; IPaccession=IPR024298(Ancestral coatomer element 1, Sec16/Sec31)
Gene3D; -; IPaccession=-(-)
Gene3D; -; IPaccession=-(-)
PANTHER; SWR1-COMPLEX PROTEIN 3; IPaccession=IPR037651(SWR1-complex protein 3)
PANTHER; ATF/CREB ACTIVATOR 1; IPaccession=IPR029810(ATF/CREB activator 1/2)
PANTHER; NUCLEOPORIN NUP49/NSP49; IPaccession=IPR039079(Nucleoporin Nup49)
SUPERFAMILY; NAD(P)-linked oxidoreductase; IPaccession=IPR036812(NADP-dependent oxidoreductase domain superfamily)
Gene3D; -; IPaccession=IPR036812(NADP-dependent oxidoreductase domain superfamily)
Pfam; Aldo/keto reductase family; IPaccession=IPR023210(NADP-dependent oxidoreductase domain)
CDD; AKR_EcYajO-like; IPaccession=-(-)
PANTHER; NADH-SPECIFIC METHYLGLYOXAL REDUCTASE-RELATED; IPaccession=-(-)
PANTHER; OXIDOREDUCTASE; IPaccession=-(-)
Pfam; CFEM domain; IPaccession=IPR008427(Extracellular membrane protein, CFEM domain)
SMART; 2CFEM; IPaccession=IPR008427(Extracellular membrane protein, CFEM domain)
ProSiteProfiles; Yeast PIR proteins repeats profile.; IPaccession=IPR000420(Yeast PIR protein repeat)
PANTHER; LYSINE-SPECIFIC DEMETHYLASE; IPaccession=-(-)
PANTHER; LD02225P; IPaccession=-(-)
Pfam; Mediator complex subunit 2; IPaccession=IPR021017(Mediator complex, subunit Med2, fungi)
SUPERFAMILY; GAT-like domain; IPaccession=-(-)
ProSiteProfiles; ENTH domain profile.; IPaccession=IPR013809(ENTH domain)
SMART; enth_2; IPaccession=IPR013809(ENTH domain)
Pfam; ANTH domain; IPaccession=IPR011417(AP180 N-terminal homology (ANTH) domain)
CDD; ANTH_N_YAP180; IPaccession=-(-)
PANTHER; PHOSPHATIDYLINOSITOL-BINDING CLATHRIN ASSEMBLY PROTEIN LAP; IPaccession=-(-)
Gene3D; ANTH domain; IPaccession=IPR014712(ANTH domain superfamily)
PANTHER; CLATHRIN ASSEMBLY PROTEIN; IPaccession=-(-)
PRINTS; Proline rich extensin signature; IPaccession=-(-)
PANTHER; PERMEASE-RELATED; IPaccession=-(-)
ProSiteProfiles; LIS1 homology (LisH) motif profile.; IPaccession=IPR006594(LIS1 homology motif)
SUPERFAMILY; Winged helix DNA-binding domain; IPaccession=IPR036390(Winged helix DNA-binding domain superfamily)
SMART; hsfneu3; IPaccession=IPR000232(Heat shock factor (HSF)-type, DNA-binding)
PANTHER; HEAT SHOCK TRANSCRIPTION FACTOR; IPaccession=IPR027725(Heat shock transcription factor family)
ProSitePatterns; HSF-type DNA-binding domain signature.; IPaccession=IPR000232(Heat shock factor (HSF)-type, DNA-binding)
Pfam; HSF-type DNA-binding; IPaccession=IPR000232(Heat shock factor (HSF)-type, DNA-binding)
PRINTS; Heat shock factor (HSF) domain signature; IPaccession=IPR000232(Heat shock factor (HSF)-type, DNA-binding)
Coils; Coil; IPaccession=-(-)
MobiDBLite; consensus disorder prediction; IPaccession=-(-)
PANTHER; OLEATE-ACTIVATED TRANSCRIPTION FACTOR 1-RELATED; IPaccession=-(-)
PANTHER; MULTIDRUG RESISTANCE REGULATOR 1; IPaccession=-(-)
PANTHER; FINGER DOMAIN PROTEIN, PUTATIVE-RELATED; IPaccession=-(-)
CDD; fungal_TF_MHR; IPaccession=-(-)
ProSitePatterns; Zn(2)-C6 fungal-type DNA-binding domain signature.; IPaccession=IPR001138(Zn(2)-C6 fungal-type DNA-binding domain)
SMART; gal4_2; IPaccession=IPR001138(Zn(2)-C6 fungal-type DNA-binding domain)
Pfam; Fungal Zn(2)-Cys(6) binuclear cluster domain; IPaccession=IPR001138(Zn(2)-C6 fungal-type DNA-binding domain)
ProSiteProfiles; Zn(2)-C6 fungal-type DNA-binding domain profile.; IPaccession=IPR001138(Zn(2)-C6 fungal-type DNA-binding domain)
CDD; GAL4; IPaccession=IPR001138(Zn(2)-C6 fungal-type DNA-binding domain)
Gene3D; -; IPaccession=IPR036864(Zn(2)-C6 fungal-type DNA-binding domain superfamily)
SUPERFAMILY; Zn2/Cys6 DNA-binding domain; IPaccession=IPR036864(Zn(2)-C6 fungal-type DNA-binding domain superfamily)

Supplementary Figure 2. Many Interproscan annotations are enriched in multiple species across genes under selection. This heatmap represents the Interproscan annotations enriched in genes under selection in >1 species. This is shown in a similar way to **Figure 4**. The enrichment p values were calculated with a one-sided Fisher's exact test, followed by False Discovery Rate (FDR)-based correction. The domains are clustered according to the Jaccard distance between the OGs affected in different sets of genes. The circles represent the species where each pathway was found to be enriched, which is useful to see functions altered in multiple taxa. Note that **Supplementary Table 2** includes all the enrichments.

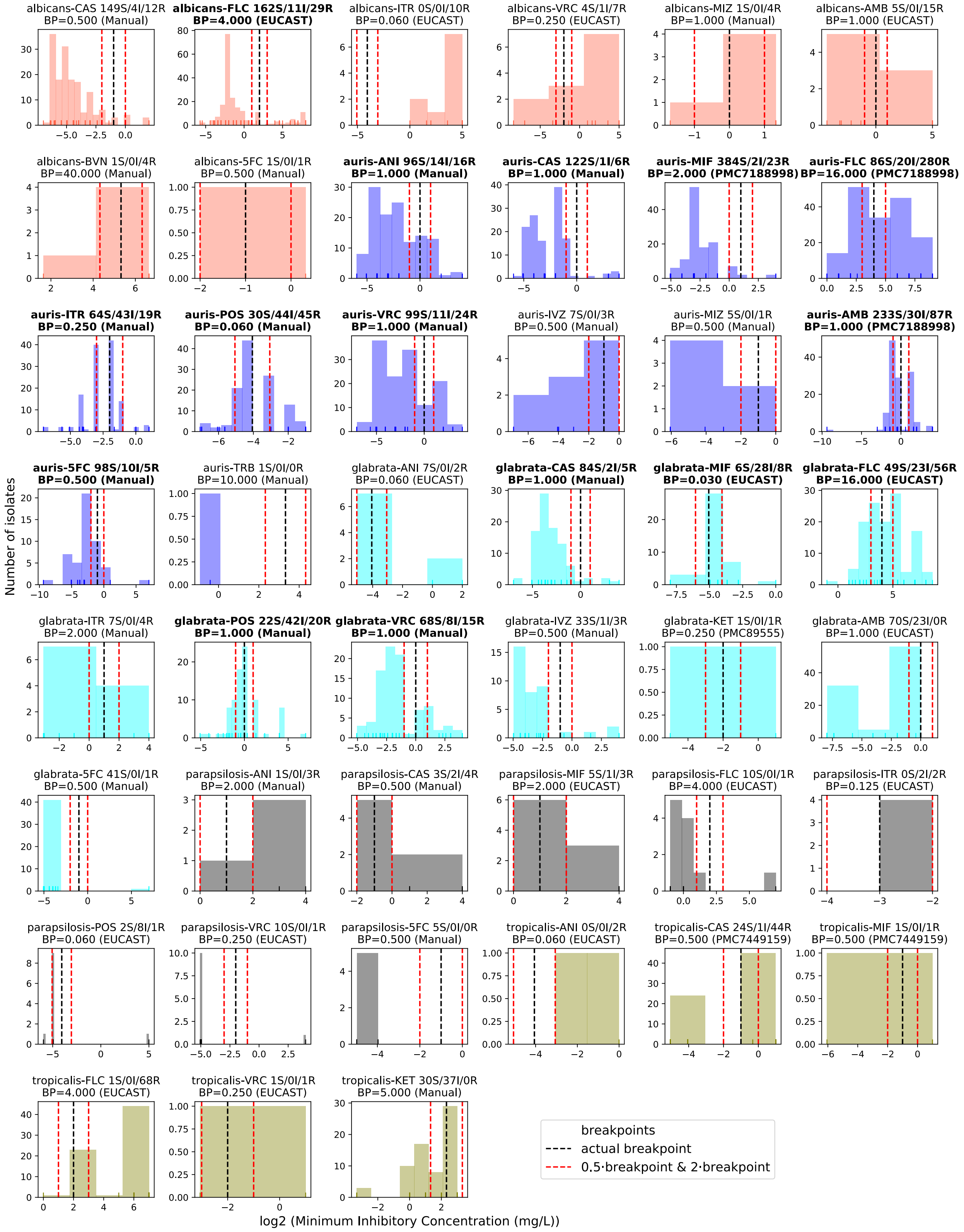
albicans-CAS 149S/4I/12R BP=0.500 (Manual)
albicans-FLC 162S/11I/29R BP=4.000 (EUCAST)
albicans-ITR 0S/0I/10R BP=0.060 (EUCAST)
albicans-VRC 4S/1I/7R BP=0.250 (EUCAST)
albicans-MIZ 1S/0I/4R BP=1.000 (Manual)
albicans-AMB 5S/0I/15R BP=1.000 (EUCAST)
albicans-BVN 1S/0I/4R BP=40.000 (Manual)
albicans-5FC 1S/0I/1R BP=0.500 (Manual)
auris-ANI 96S/14I/16R BP=1.000 (Manual)
auris-CAS 122S/1I/6R BP=1.000 (Manual)
auris-MIF 384S/2I/23R BP=2.000 (PMC7188998)
auris-FLC 86S/20I/280R BP=16.000 (PMC7188998)
auris-ITR 64S/43I/19R BP=0.250 (Manual)
auris-POS 30S/44I/45R BP=0.060 (Manual)
auris-VRC 99S/11I/24R BP=1.000 (Manual)
auris-IVZ 7S/0I/3R BP=0.500 (Manual)
auris-MIZ 5S/0I/1R BP=0.500 (Manual)
auris-AMB 233S/30I/87R BP=1.000 (PMC7188998)
auris-5FC 98S/10I/5R BP=0.500 (Manual)
auris-TRB 1S/0I/0R BP=10.000 (Manual)
glabrata-ANI 7S/0I/2R BP=0.060 (EUCAST)
glabrata-CAS 84S/2I/5R BP=1.000 (Manual)
glabrata-MIF 6S/28I/8R BP=0.030 (EUCAST)
glabrata-FLC 49S/23I/56R BP=16.000 (EUCAST)
glabrata-ITR 7S/0I/4R BP=2.000 (Manual)
glabrata-POS 22S/42I/20R BP=1.000 (Manual)
glabrata-VRC 68S/8I/15R BP=1.000 (Manual)
glabrata-IVZ 33S/1I/3R BP=0.500 (Manual)
glabrata-KET 1S/0I/1R BP=0.250 (PMC89555)
glabrata-AMB 70S/23I/0R BP=1.000 (EUCAST)
glabrata-5FC 41S/0I/1R BP=0.500 (Manual)
parapsilosis-ANI 1S/0I/3R BP=2.000 (Manual)
parapsilosis-CAS 3S/2I/4R BP=0.500 (Manual)
parapsilosis-MIF 5S/1I/3R BP=2.000 (EUCAST)
parapsilosis-FLC 10S/0I/1R BP=4.000 (EUCAST)
parapsilosis-ITR 0S/2I/2R BP=0.125 (EUCAST)
parapsilosis-POS 2S/8I/1R BP=0.060 (EUCAST)
parapsilosis-VRC 10S/0I/1R BP=0.250 (EUCAST)
parapsilosis-5FC 5S/0I/0R BP=0.500 (Manual)
tropicalis-ANI 0S/0I/2R BP=0.060 (EUCAST)
tropicalis-CAS 24S/1I/44R BP=0.500 (PMC7449159)
tropicalis-MIF 1S/0I/1R BP=0.500 (PMC7449159)
tropicalis-FLC 1S/0I/68R BP=4.000 (EUCAST)
tropicalis-VRC 1S/0I/1R BP=0.250 (EUCAST)
tropicalis-KET 30S/37I/0R BP=5.000 (Manual)
breakpoints
actual breakpoint
0.5·breakpoint & 2·breakpoint
Number of isolates
log2 (Minimum Inhibitory Concentration (mg/L))

Supplementary Figure 3. **Definition of drug resistant (R) and susceptible (S) strains.** Per-strain distribution of Minimum Inhibitory Concentrations (MICs), in log_2 space, for various species and drugs in which this data was available. The colors represent different species. The drugs are amphotericin B (AMB), beauvericin (BVN), 5-flucytosine (5FC), terbinafine (TRB), miconazole (MIZ), ketoconazole (KET), isavuconazole (IVZ), voriconazole (VRC), posaconazole (POS), itraconazole (ITR), fluconazole (FLC), micafungin (MIF), caspofungin (CAS) and anidulafungin (ANI). We defined as strains with high resistance (R) those that had a MIC ≥ 2·breakpoint, and as strains with high susceptibility (S) those with a MIC ≤0.5·breakpoint. The vertical black lines indicate the actual breakpoints, and the red lines indicate these thresholds above and below the breakpoint. We set the breakpoints (BP in the title) either using EUCAST recommendations (https://www.eucast.org/), based on previous studies (PMC7449159[28], PMC89555[29] and PMC7188998[30]) or by manually looking at the distribution (specified as 'Manual' in the title). We performed GWAS on datasets with at least 5R and 5S clinical strains (plots with bold titles), except in the *C. tropicalis* data because the MIC inference was non-standard[31] (see also **Online Methods**). In the title, S/I/R indicate the number of strains belonging to each susceptibility type ('I' means intermediate susceptibility). Note that these S/I/R numbers also include strains in which MIC was not available, but instead we found explicit reports of resistance/susceptibility.

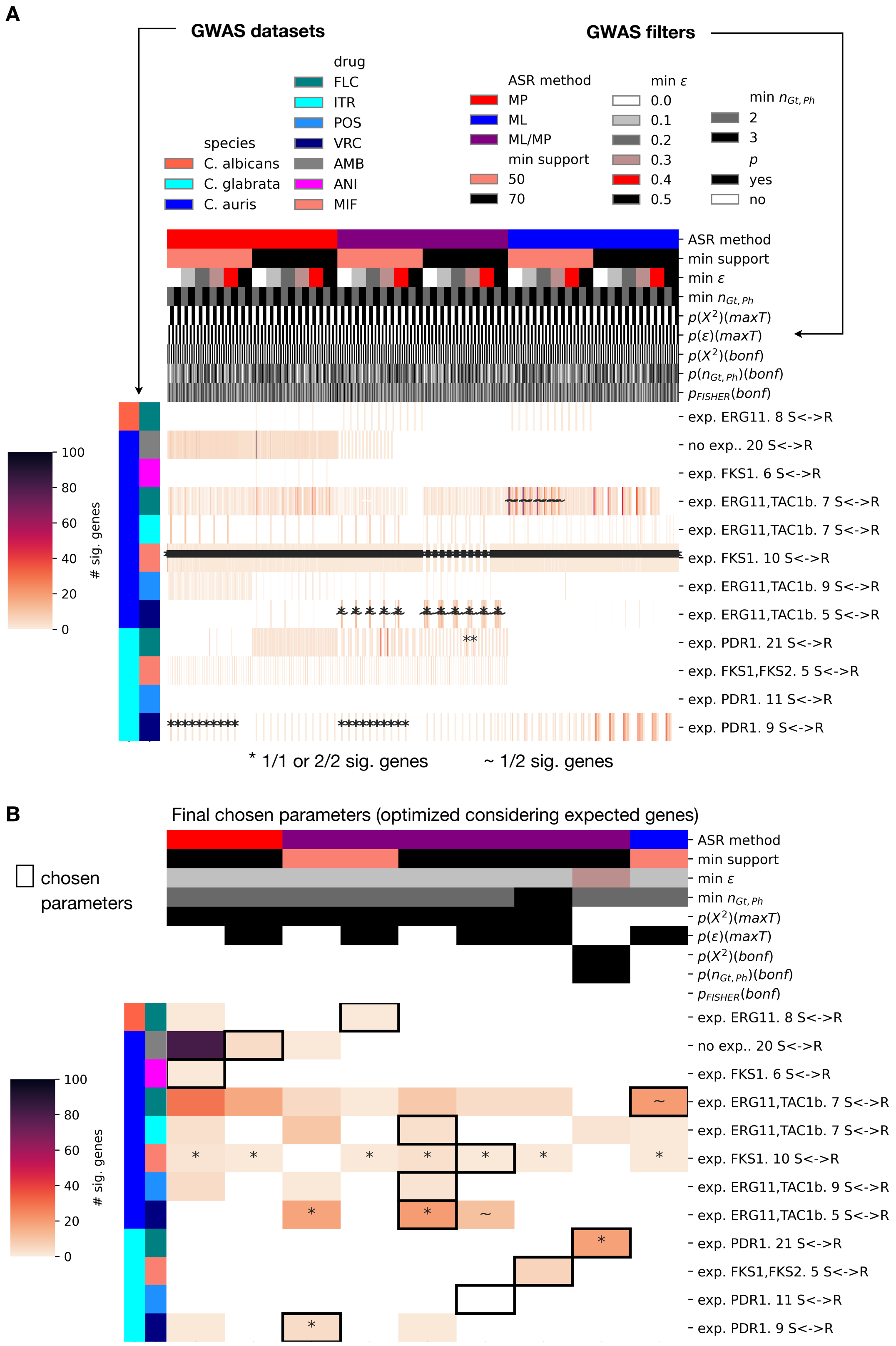
A
GWAS datasets
GWAS filters
species
C. albicans
C. glabrata
C. auris
drug
FLC
ITR
POS
VRC
AMB
ANI
MIF
ASR method
MP
ML
ML/MP
min support
50
70
min ε
0.0
0.1
0.2
0.3
0.4
0.5
min $n_{Gt,Ph}$
2
3
p
yes
no
ASR method
min support
min ε
min $n_{Gt,Ph}$
$p(X^2)(maxT)$
$p(\varepsilon)(maxT)$
$p(X^2)(bonf)$
$p(n_{Gt,Ph})(bonf)$
$p_{FISHER}(bonf)$
sig. genes
100
80
60
40
20
0
exp. ERG11. 8 S<->R
no exp.. 20 S<->R
exp. FKS1. 6 S<->R
exp. ERG11,TAC1b. 7 S<->R
exp. ERG11,TAC1b. 7 S<->R
exp. FKS1. 10 S<->R
exp. ERG11,TAC1b. 9 S<->R
exp. ERG11,TAC1b. 5 S<->R
exp. PDR1. 21 S<->R
exp. FKS1,FKS2. 5 S<->R
exp. PDR1. 11 S<->R
exp. PDR1. 9 S<->R
* 1/1 or 2/2 sig. genes
~ 1/2 sig. genes
B
Final chosen parameters (optimized considering expected genes)
chosen parameters
ASR method
min support
min ε
min $n_{Gt,Ph}$
$p(X^2)(maxT)$
$p(\varepsilon)(maxT)$
$p(X^2)(bonf)$
$p(n_{Gt,Ph})(bonf)$
$p_{FISHER}(bonf)$
sig. genes
100
80
60
40
20
0
exp. ERG11. 8 S<->R
no exp.. 20 S<->R
exp. FKS1. 6 S<->R
exp. ERG11,TAC1b. 7 S<->R
exp. ERG11,TAC1b. 7 S<->R
exp. FKS1. 10 S<->R
exp. ERG11,TAC1b. 9 S<->R
exp. ERG11,TAC1b. 5 S<->R
exp. PDR1. 21 S<->R
exp. FKS1,FKS2. 5 S<->R
exp. PDR1. 11 S<->R
exp. PDR1. 9 S<->R

C

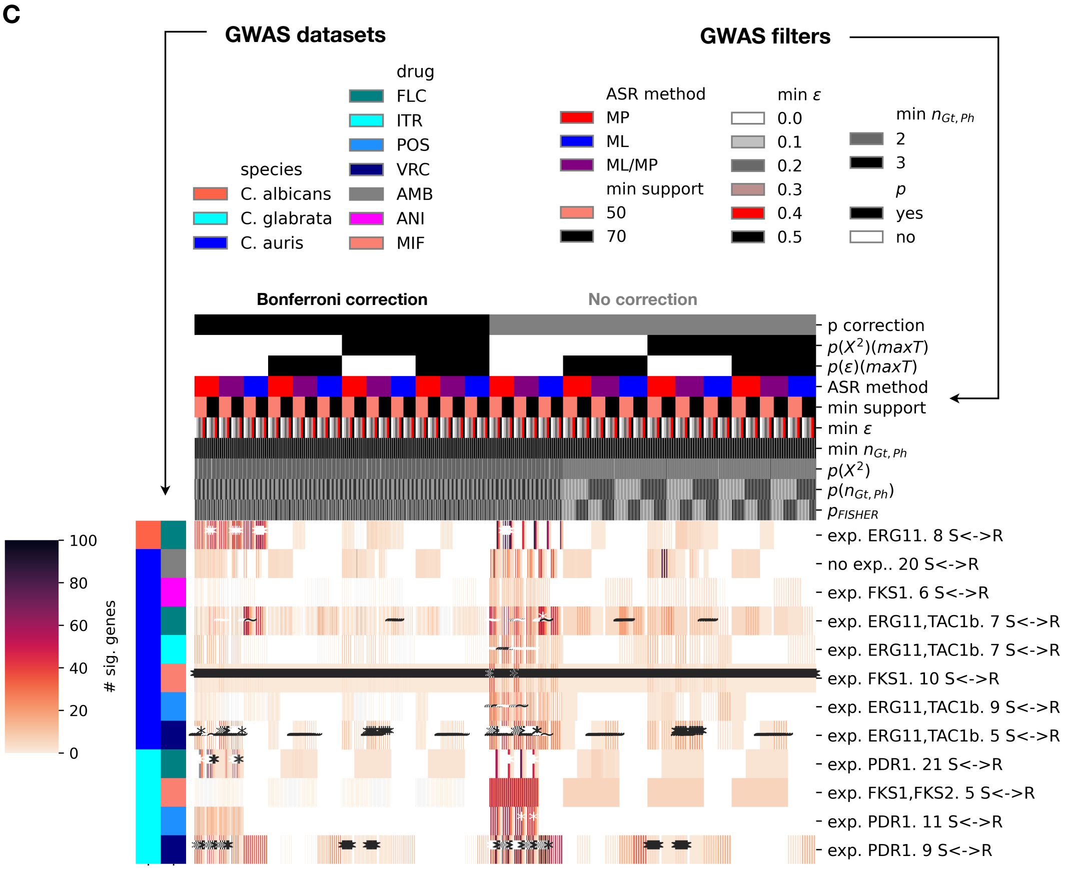
GWAS datasets
GWAS filters
species
C. albicans
C. glabrata
C. auris
drug
FLC
ITR
POS
VRC
AMB
ANI
MIF
ASR method
MP
ML
ML/MP
min support
50
70
min ε
0.0
0.1
0.2
0.3
0.4
0.5
min $n_{Gt,Ph}$
2
3
p
yes
no
Bonferroni correction
No correction
p correction
$p(X^2)(maxT)$
$p(\varepsilon)(maxT)$
ASR method
min support
min ε
min $n_{Gt,Ph}$
$p(X^2)$
$p(n_{Gt,Ph})$
p_{FISHER}
sig. genes
100
80
60
40
20
0
exp. ERG11. 8 S<->R
no exp.. 20 S<->R
exp. FKS1. 6 S<->R
exp. ERG11,TAC1b. 7 S<->R
exp. ERG11,TAC1b. 7 S<->R
exp. FKS1. 10 S<->R
exp. ERG11,TAC1b. 9 S<->R
exp. ERG11,TAC1b. 5 S<->R
exp. PDR1. 21 S<->R
exp. FKS1,FKS2. 5 S<->R
exp. PDR1. 11 S<->R
exp. PDR1. 9 S<->R

Supplementary Figure 4. Various GWAS parameters and filtering criteria largely alter the resulting hits. (A) Heatmap showing how different parameter/filter combinations (columns) yield various numbers of genes with significant GWAS hits (color scale) in each dataset (rows) (see **Online Methods**). As GWAS parameters, we varied both the Ancestral State Reconstruction (ASR) method (using methods based on either Maximum Parsimony (MP), Maximum Likelihood (ML) or a ML/MP consensus) and the minimum branch support to consider nodes (50 or 70). As filters, we considered filtering based on any combination of five p values (requiring them to be <0.05 for significance): *p*(X^2)*, (maxT), p(ε), (maxT), p*(X^2) *(bonferroni-corrected)*, *p*($n_{Gt,Ph}$) *(bonferroni-corrected)* and/or p_{FISHER} *(bonferroni-corrected)*. Note that all these p values come from one-sided tests. The *p*(X^2)*, (maxT), p(ε), (maxT), p*(X^2) *(bonferroni-corrected)* and *p*($n_{Gt,Ph}$) *(bonferroni-corrected)* are empirical p values, while p_{FISHER} *(bonferroni-corrected)* was calculated with a Fisher's exact test (see **Online Methods**). In addition we applied various thresholds on the minimum ε (0-0.5) and the minimum $n_{Gt,Ph}$ (2 or 3). The symbols represent the fraction of expected genes (*ERG11, FKS, PDR1* and *TAC1b*, shown in the y ticks) that have significant associations ('*' means 1/1 or 2/2 genes and '~' means 1/2 genes). The number in the ticks (i.e. 7 S<->R) represent the number of high-confidence phenotype transitions observed in each dataset. The yes/no boxes indicate which types of p values where required to be p<0.05 for significance. Note that any instance with >100 significant genes or without proper multiple testing correction (inferred with an empiric approach described in **Online Methods**) are set to 0 (blank cells) in this plot. The drugs are fluconazole (FLC), itraconazole (ITR), posaconazole (POS), voriconazole (VRC), anidulafungin (ANI), micafungin (MIF) and amphotericin B (AMB). **(B)** The same as in (A), but showing only the subset of filters that we chose to define high-confidence hits in each dataset (see **Online Methods**). The black boxes indicate these chosen filters. In the **Supplementary results** we discuss why we don't find the expected genes in some echinocandins and azoles. **(C)** The same as (A), but showing the effect of the non-corrected p values (right columns) in addition to the bonferroni-corrected ones (left columns). In addition, to generate this plot we did not consider whether the multiple testing burden is addressed (see **Online Methods**). Note that the *maxT* p values are readily corrected for multiple testing, and we reordered the column color boxes (as compared to (A)) to show the results of different combinations of correction methods (considering bonferroni-corrected and/or *maxT* p-values).

SUPPLEMENTARY TABLE LEGENDS

Supplementary Table 1. Strains used in this study. The tab **'All strains'** includes the metadata for all strains used in this study. We include the species, BioProject, Run, strain, BioSample, numeric sample ID, clade, clonal cluster, type of strain, collection date, collection location, patient ID and collection time point for clinical serial isolates, coverage, link of the paper from which metadata was obtained and susceptibility profiles. The antifungal drugs are amphotericin B (AMB), beauvericin (BVN), 5-flucytosine (5FC), terbinafine (TRB), miconazole (MIZ), ketoconazole (KET), isavuconazole (IVZ), voriconazole (VRC), posaconazole (POS), itraconazole (ITR), fluconazole (FLC), micafungin (MIF), caspofungin (CAS) and anidulafungin (ANI). We report the MIC_{50} for all of them except AMB, which includes MIC_{90}. The 'resistance', 'susceptibility' and 'intermediante_susceptibility' columns indicate the discrete susceptibility profile (for the tested drugs) either stated in the literature or derived from breakpoints on the MIC data (see **Online Methods**). The 'strain' column refers to available strain annotations from the NCBI (missing for a fraction of runs). The 'type' column is either 'clinical' (isolated from patients), 'environmental' (from soil or sea), 'genome_engineered' (strains that had some genetic engineering), 'genome_engineered/inmouse_evol_clone' (strains that had some genetic engineering and underwent *in-mouse* evolution), 'inmouse_evol_clone' (strains that underwent *in-mouse* evolution), 'invitro_evol_clone' (strains that underwent *in-vitro* evolution), 'invitro_evol_population' (strains that underwent *in-vitro* evolution and the whole population was sequenced) , 'one_homozygous_chromosome' (strains with one homozygous chromosome) or 'reference' (reference strains). The 'mean_coverage' is the mean read depth across windows of the genome. The 'pct_covered' is the mean percentage of the window covered across windows of the genome. Note that the strains here are all the ones for which we did variant calling except two *C. auris* samples that may be a mix of divergent lineages (see **Online Methods**). The column 'cladeID_systematic' indicates the systematically-defined clades from this work (see **Online Methods**). The column 'cladeID_previous' indicates the clades defined in previous studies (see **Online Methods**). Finally, the 'clonal_cluster' indicates the cluster of close strains (used in the selection analysis (**Figure 3**)) to which each strain belongs. The tab '**Strains overview**' includes the number of strains per species ('# strains'), the numbers of each type of strain ('# clinical', '# environmental' and '# other'), the number of clade ('# clades') and the average intraspecific diversity ('median pairwise SNPs/kb'). The tab '**GWAS drugs overview**' shows, for each species and drug where we performed a GWAS, the numbers of resistant (R) and susceptible (S) strains, the number of resistance phenotype transitions ('R>S or S>R transitions'), and the fraction of clades that have some resistant or susceptible strain. The tab '**Reference genomes**' tab has the information about the reference genomes and annotations used. We merged the gDNA and mtDNA if their source was not the same to get the reference genome. Note that the gff annotations were taken from the equivalent source for each gDNA and mtDNA. CGD stands for Candida Genome Database. The 'CGD gene features source' refers to the CGD table with chromosomal feature files

from http://www.candidagenome.org/download/chromosomal_feature_files/. The 'CGD GO annotations file' is the name of the file at http://www.candidagenome.org/download/go/archive from which we got Gene Ontology annotations. The tab '**Strain trees**' shows the trees generated for each strain in newick format, based on the numeric sample IDs. The tab '**GWAS confirmation strains**' is equivalent to '**All strains**', but with the clinical isolates that were used for the validation of high-confidence GWAS hits (see **Supplementary Results** and **Online Methods**). Note that this tab has the MIC data and also the discrete I/S/R susceptibility information (see **Supplementary Figure 3** and **Online Methods** for clarification). Note that in the github repository of this project (see **Data and Code Availability**) we provide the csv versions of all these excel tabs, which are more suited for large-scale reanalysis.

Supplementary Table 2. Recent selection data. The tab '**Genes under selection'** includes the selection scores and p values for all genes with a significantly high selection score (*S*), meaning that they have an excess of either recent non-synonymous SNPs, deletions (DEL), duplications (DUP) or in-frame INDELs (if_INDEL). This type of variant is indicated in the column 'type_var'. The selection score *S* (column 'selection_score_S') is calculated as the harmonic mean between the fraction of strains with signs of positive selection in the gene ($\pi_N > \pi_S$ for SNP and variant presence for DEL/DUP/if_INDEL) (column 'fraction_strains_w_selection') and the fraction of clonal strain clusters that have some strain with a sign of positive selection (column 'fraction_clusters_w_selection'). The columns 'total_number_strains' and 'total_number_clusters' indicate the absolute number of strains and clusters used to calculate *S*. In addition, 'significant_selection' is a TRUE/FALSE boolean showing whether the gene has significantly high *S*. For SNPs, the 'fdr_p_S' represents the FDR-corrected p value of observing a selection score $\geq S$ under a neutral model of evolution. Columns 'chromosome', 'start', 'end', 'gene_name', 'Scerevisiae_orthologs' and 'description' are gene features obtained from CGD. The column 'gff_upmost_parent' indicates the ID of the gene in the gff file, which is our proxy for gene ID. The column 'orthofinder_orthocluster' is the ID of the Orthologous Group (OG) in which the gene belongs, according to orthofinder. We used these to calculate the number of species that have genes of this OG under selection (column 'n_species_orthogroup') and the number of types of variants that yield a gene under selection in this OG in a given species (column 'n_types_vars_in_species_orthogroup'). Finally, the columns 'biological_process_GO', 'molecular_function_GO' and 'cellular_component_GO' indicate the annotated Gene Ontology terms. The tab '**Genes under selection >1 species'** is a subset of the 'Genes under selection**'** tab (with the same columns), only including genes where the OG has genes under selection in >1 species. The tab '**Selection scores all genes**' is equivalent to the 'Genes under selection**'** tab (with the same columns), but including all genes for which we could measure a selection score *S*. Most of these genes do not have significant signs of selection (as indicated in the column 'significant_selection'), but we provide them because they may be useful to validate the *S* values for specific genes. The tab '**Functional enrichments**' includes the Gene Ontology (GO) terms, Interproscan annotations, MetaCyc and Reactome pathways enriched in the sets of

genes under selection by different species and types of variants. The column 'type_grouping' indicates the type of enrichment performed: GO Biological Process (GO_BP), GO Molecular Function (GO_MF), GO Cellular Component (GO_CC), Interposcan annotations (IP_domains), Metacyc or Reactome. 'ID' is the identifier of the enriched group. The columns 'ngenes_group_and_target', 'ngenes_no_group_target', 'ngenes_group_no_target', 'ngenes_no_group_no_target' indicate the numbers of genes belonging to the target set (genes under selection) and/or to the group (i.e. genes with a given GO term), which were used for the association test. 'OR' and 'p_raw' are the odds-ratio and p value resulting from the Fisher test, and 'p_fdr' is the FDR-corrected p value. The 'group_name' indicates the annotation description. Finally, the column 'genes' indicates the set of genes that drive the enrichment (they belong to the group tested and are also under selection by a given species and type of variant). Note that we considered as 'significant enrichments' those that had a p_fdr<0.05 and an OR ≥ 2. The tab '**Gene features**' includes the gene name, *S. cerevisiae* ortholog, orthofinder OG and description for all genes (where the gene ID is 'gff_upmost_parent'). This tab is useful to do further analyses where linking between genes and OGs is required. Note that in the github repository of this project (see **Data and Code Availability**) we provide the csv versions of all these excel tabs, which are more suited for large-scale reanalysis.

Supplementary Table 3. GWAS associations. The tab '**High-confidence GWAS hits**' includes all the GWAS hits that passed the strict, high-confidence filters (see **Online Methods**). Each row is one hit (it may be a variant, a domain, a gene or a pathway). This list is non-redundant, meaning that there is only one hit per gene or pathway in each GWAS dataset, and it includes hits for pathways that don't have any belonging gene as a hit. The columns 'type_vars', 'type_mutations' and 'type_collapsing' refer to the collapsing strategy used to find that hit (see **Online Methods** and **Figure 6**). The column 'group_name' is the unique identifier of the hit. For domain-level collapsing, it includes the ID of the domain, the gene name, and the range of the protein altered. The columns 'epsilon' and 'OR' indicate the strength of the association. The columns 'nodes_GenoAndPheno', 'nodes_noGenoAndNoPheno', 'nodes_GenoAndNoPheno' and 'nodes_noGenoAndPheno' indicate the numbers of nodes that have a genotype transition and/or a phenotype transition. In addition, 'orthogroups' refers to the Orthologous Groups (OGs) affected by that hit (only relevant for variants, domains and genes). The column 'n_spp_drug_worthogroups' refers to the number of GWAS datasets that have a hit affecting these OGs. Similarly, 'n_spp_drug_wpathway' indicates, for pathway hits, the number of GWAS datasets that have a hit involving a given pathway. On another line, the fields 'pval_chi_square_maxT', 'pval_epsilon_maxT', 'pval_chi_square_phenotypes', 'pval_GenoAndPheno_phenotypes' and 'pval_fisher' are the raw p values (one-sided) for each hit, while the 'pval_chi_square_phenotypes_bonferroni', 'pval_GenoAndPheno_phenotypes_bonferroni' and 'pval_fisher_bonferroni' include the corresponding bonferroni-corrected p values. Note that the 'maxT' p values are already corrected for multiple testing. Finally, 'description' is a text that indicates what is the hit, while 'biological_process_GO', 'cellular_component_GO', molecular_function_GO' are the GO terms

annotated for each gene (only relevant for variants, genes and domains). The tab '**High-confidence GWAS hits >1 dataset**' includes a subset of high-confidence hits (from 'High-confidence GWAS hits') where the implicated OGs or pathways are significant in >1 dataset. The tab '**Low-confidence GWAS hits**' includes the GWAS hits obtained with relaxed filters. There may be some false positives here (so that this tab is not useful for exploratory analysis), but these low-confidence hits may be suited to test hypotheses about specific drug resistance genes. The columns of this tab are the same as 'High-confidence GWAS hits', but also including 'ASR_method' and 'min_support', which refer to the GWAS parameters that yielded each hit. This table includes stacked low-confidence hits for six combinations of parameters, since 'ASR_method' can be 'DOWNPASS', 'MPPA' or 'MPPA,DOWNPASS' and 'min_support' can be '50' or '70'. This means that some hits could appear multiple times, since the different parameters often yield similar results. Note that in the github repository of this project (see **Data and Code Availability**) we provide the csv versions of all these excel tabs, which are more suited for large-scale reanalysis.

SUPPLEMENTARY REFERENCES

1. Galocha, M. *et al.* Divergent Approaches to Virulence in *C. albicans* and *C. glabrata*: Two Sides of the Same Coin. *Int. J. Mol. Sci.* **20**, 2345 (2019).
2. Brunke, S. *et al.* One small step for a yeast--microevolution within macrophages renders *Candida glabrata* hypervirulent due to a single point mutation. *PLoS Pathog.* **10**, e1004478 (2014).
3. Rai, M. N., Rai, R., Sethiya, P. & Parsania, C. Transcriptome analysis reveals a common adaptive transcriptional response of *Candida glabrata* to diverse environmental stresses. *Res. Microbiol.* 104073 (2023) doi:10.1016/j.resmic.2023.104073.
4. Bhakt, P., Shivarathri, R., Choudhary, D. K., Borah, S. & Kaur, R. Fluconazole-induced actin cytoskeleton remodeling requires phosphatidylinositol 3-phosphate 5-kinase in the pathogenic yeast *Candida glabrata*. *Mol. Microbiol.* **110**, 425–443 (2018).
5. Moseley, J. B. & Goode, B. L. The Yeast Actin Cytoskeleton: from Cellular Function to Biochemical Mechanism. *Microbiol. Mol. Biol. Rev.* **70**, 605–645 (2006).
6. Jain, P., Akula, I. & Edlind, T. Cyclic AMP Signaling Pathway Modulates Susceptibility of *Candida* Species and *Saccharomyces cerevisiae* to Antifungal Azoles and Other Sterol Biosynthesis Inhibitors. *Antimicrob. Agents Chemother.* **47**, 3195–3201 (2003).
7. Gabaldón, T., Naranjo-Ortíz, M. A. & Marcet-Houben, M. Evolutionary genomics of yeast pathogens in the Saccharomycotina. *FEMS Yeast Res.* **16**, fow064 (2016).
8. Zamith-Miranda, D. *et al.* Transcriptional and translational landscape of *Candida auris* in response to caspofungin. *Comput. Struct. Biotechnol. J.* **19**, 5264–5277 (2021).
9. Bruckmann, A., Künkel, W., Härtl, A., Wetzker, R. & Eck, R. A phosphatidylinositol 3-kinase of *Candida albicans* influences adhesion, filamentous growth and virulence. *Microbiol. Read. Engl.* **146 (Pt 11)**, 2755–2764 (2000).
10. Wang, Y. *et al.* Innate immune responses against the fungal pathogen *Candida auris*. *Nat. Commun.* **13**, 3553 (2022).

11. Casagrande Pierantoni, D. *et al.* How does temperature trigger biofilm adhesion and growth in *Candida albicans* and two non-*Candida albicans Candida* species? *Mycoses* **64**, 1412–1421 (2021).

12. Ma, T. *et al.* Role of the inositol polyphosphate kinase Vip1 in autophagy and pathogenesis in *Candida albicans*. *Future Microbiol.* **15**, 1363–1377 (2020).

13. Spettel, K. *et al.* Analysis of antifungal resistance genes in *Candida albicans* and *Candida glabrata* using next generation sequencing. *PloS One* **14**, e0210397 (2019).

14. Garnaud, C., Champleboux, M., Maubon, D., Cornet, M. & Govin, J. Histone Deacetylases and Their Inhibition in *Candida* Species. *Front. Microbiol.* **7**, 1238 (2016).

15. Mora-Montes, H. M. *et al.* Protein glycosylation in *Candida*. *Future Microbiol.* **4**, 1167–1183 (2009).

16. Van Ende, M., Wijnants, S. & Van Dijck, P. Sugar Sensing and Signaling in *Candida albicans* and *Candida glabrata*. *Front. Microbiol.* **10**, (2019).

17. Heimark, L. *et al.* Mechanism of azole antifungal activity as determined by liquid chromatographic/mass spectrometric monitoring of ergosterol biosynthesis. *J. Mass Spectrom. JMS* **37**, 265–269 (2002).

18. Ni, Q. *et al.* CgPDR1 gain-of-function mutations lead to azole-resistance and increased adhesion in clinical *Candida glabrata* strains. *Mycoses* **61**, 430–440 (2018).

19. Rybak, J. M. *et al.* Mutations in TAC1B: a Novel Genetic Determinant of Clinical Fluconazole Resistance in *Candida auris*. *mBio* **11**, e00365-20 (2020).

20. Perlin, D. S. Resistance to echinocandin-class antifungal drugs. *Drug Resist. Updat. Rev. Comment. Antimicrob. Anticancer Chemother.* **10**, 121–130 (2007).

21. Ksiezopolska, E. & Gabaldón, T. Evolutionary Emergence of Drug Resistance in *Candida* Opportunistic Pathogens. *Genes* **9**, 461 (2018).

22. Williamson, B. *et al.* Impact of Erg11 Amino Acid Substitutions Identified in *Candida auris* Clade III Isolates on Triazole Drug Susceptibility. *Antimicrob. Agents Chemother.* **66**, e0162421 (2022).

23. Rybak, J. M. *et al.* Delineation of the Direct Contribution of *Candida auris* ERG11 Mutations to Clinical Triazole Resistance. *Microbiol. Spectr.* **9**, e01585-21.

24. Matin, M. M. *et al.* Triazoles and Their Derivatives: Chemistry, Synthesis, and Therapeutic Applications. *Front. Mol. Biosci.* **9**, 864286 (2022).

25. Ksiezopolska, E. *et al.* Narrow mutational signatures drive acquisition of multidrug resistance in the fungal pathogen *Candida glabrata*. *Curr. Biol. CB* **31**, 5314-5326.e10 (2021).

26. Galocha, M. *et al.* Genomic evolution towards azole resistance in *Candida glabrata* clinical isolates unveils the importance of CgHxt4/6/7 in azole accumulation. *Commun. Biol.* **5**, 1–12 (2022).

27. Vu, B. G., Stamnes, M. A., Li, Y., Rogers, P. D. & Moye-Rowley, W. S. The *Candida glabrata* Upc2A transcription factor is a global regulator of antifungal drug resistance pathways. *PLoS Genet.* **17**, e1009582 (2021).

28. McTaggart, L. R., Cabrera, A., Cronin, K. & Kus, J. V. Antifungal Susceptibility of Clinical Yeast Isolates from a Large Canadian Reference Laboratory and Application of Whole-Genome Sequence Analysis To Elucidate Mechanisms of Acquired Resistance. *Antimicrob. Agents Chemother.* **64**, e00402-20 (2020).

29. Sanglard, D., Ischer, F., Calabrese, D., Majcherczyk, P. A. & Bille, J. The ATP binding cassette transporter gene CgCDR1 from *Candida glabrata* is involved in the resistance of clinical isolates to azole antifungal agents. *Antimicrob. Agents Chemother.* **43**, 2753–2765 (1999).

30. Chow, N. A. *et al.* Tracing the Evolutionary History and Global Expansion of *Candida auris* Using Population Genomic Analyses. *mBio* **11**, e03364-19 (2020).

31. O'Brien, C. E. *et al.* Population genomics of the pathogenic yeast *Candida tropicalis* identifies hybrid isolates in environmental samples. *PLoS Pathog.* **17**, e1009138 (2021).
