# Supplementary material for: Recent gene selection and drug resistance underscore clinical adaptation across Candida species
Source: Nat Microbiol. 2024 Jan 4;9(1):284–307. doi: 10.1038/s41564-023-01547-z (PMC10769879; doi:10.1038/s41564-023-01547-z)
Supplement: Supplementary file 2 — Reporting Summary [file 41564_2023_1547_MOESM2_ESM.pdf]

nature portfolio

Corresponding author(s): Toni Gabaldón

Last updated by author(s): Mar 5, 2023

Reporting Summary

Nature Portfolio wishes to improve the reproducibility of the work that we publish. This form provides structure for consistency and transparency in reporting. For further information on Nature Portfolio policies, see our Editorial Policies and the Editorial Policy Checklist.

Statistics

For all statistical analyses, confirm that the following items are present in the figure legend, table legend, main text, or Methods section.

n/a	Confirmed	
☐	☒	The exact sample size (*n*) for each experimental group/condition, given as a discrete number and unit of measurement
☐	☒	A statement on whether measurements were taken from distinct samples or whether the same sample was measured repeatedly
☐	☒	The statistical test(s) used AND whether they are one- or two-sided<br>*Only common tests should be described solely by name; describe more complex techniques in the Methods section.*
☒	☐	A description of all covariates tested
☐	☒	A description of any assumptions or corrections, such as tests of normality and adjustment for multiple comparisons
☐	☒	A full description of the statistical parameters including central tendency (e.g. means) or other basic estimates (e.g. regression coefficient) AND variation (e.g. standard deviation) or associated estimates of uncertainty (e.g. confidence intervals)
☐	☒	For null hypothesis testing, the test statistic (e.g. *F*, *t*, *r*) with confidence intervals, effect sizes, degrees of freedom and *P* value noted<br>*Give P values as exact values whenever suitable.*
☒	☐	For Bayesian analysis, information on the choice of priors and Markov chain Monte Carlo settings
☒	☐	For hierarchical and complex designs, identification of the appropriate level for tests and full reporting of outcomes
☒	☐	Estimates of effect sizes (e.g. Cohen's *d*, Pearson's *r*), indicating how they were calculated

Our web collection on statistics for biologists contains articles on many of the points above.

Software and code

Policy information about availability of computer code

Data collection	All the software used for data collection is described in https://github.com/Gabaldonlab/Candida_Selection_DrugResistance (section 'Software environments').
Data analysis	All the software used for data analysis is described in https://github.com/Gabaldonlab/Candida_Selection_DrugResistance (section 'Software environments').

For manuscripts utilizing custom algorithms or software that are central to the research but not yet described in published literature, software must be made available to editors and reviewers. We strongly encourage code deposition in a community repository (e.g. GitHub). See the Nature Portfolio guidelines for submitting code & software for further information.

Data

Policy information about availability of data

All manuscripts must include a data availability statement. This statement should provide the following information, where applicable:

- Accession codes, unique identifiers, or web links for publicly available datasets
- A description of any restrictions on data availability
- For clinical datasets or third party data, please ensure that the statement adheres to our policy

All the SRA sequencing datasets analyzed are in Supplementary Table S1. In addition, the variants are available at https://candidamine.org. Furthermore, the github repository https://github.com/Gabaldonlab/Candida_Selection_DrugResistance contains the csv versions of the supplementary tables.

Human research participants

Policy information about studies involving human research participants and Sex and Gender in Research.

Reporting on sex and gender	Not applicable.
Population characteristics	Not applicable.
Recruitment	Not applicable.
Ethics oversight	Not applicable.

Note that full information on the approval of the study protocol must also be provided in the manuscript.

Field-specific reporting

Please select the one below that is the best fit for your research. If you are not sure, read the appropriate sections before making your selection.

☐ Life sciences ☐ Behavioural & social sciences ☒ Ecological, evolutionary & environmental sciences

For a reference copy of the document with all sections, see nature.com/documents/nr-reporting-summary-flat.pdf

Ecological, evolutionary & environmental sciences study design

All studies must disclose on these points even when the disclosure is negative.

Study description	Study of the genomic signatures of recent selection and drug resistance in clinical isolates of six major Candida pathogens.
Research sample	All available whole-genome sequencing datasets at the NCBI Sequence Read Archive for Candida auris, Candida albicans, Candida glabrata, Candida tropicalis, Candida parapsilosis and Candida orthopsilosis. For the analyses that required phenotypes, we obtained them by manually curating the literature associated to each of the sequenced genomes.
Sampling strategy	We did not calculate required sample sizes, since our approach was to take all available sequenced isolates at the time of preparing this manuscript.
Data collection	We obtained the data from the SRA automatically with the API. In addition, we manually curated the associated literature to obtain phenotypes.
Timing and spatial scale	Not applicable.
Data exclusions	We filtered out low quality samples having a read depth <40x or covering <90% of the reference genome. In addition, we discarded two Candida auris samples that were likely a mix of divergent strains (described in the Online Methods). These filtering criteria were pre-established.
Reproducibility	We provide the code and software environments used to generate the datasets, results, tables and figures presented in this paper (in https://github.com/Gabaldonlab/Candida_Selection_DrugResistance). We did not generate new experimental data here, so that the consideration of the attempts to repeat experiments does not apply.
Randomization	This is mostly not relevant for our study since our approach was to take all available sequenced isolates, so that randomization does not generally apply. However, in the GWAS analysis we used a subset of samples, which were randomly selected to cover the highest diversity possible while yielding a balanced dataset of resistant/susceptible strains.
Blinding	Blinding was not relevant because all results are based on computational mesurements, were there should be no subjective bias depending on the prior knowledge of the type of strain analyzed.

Did the study involve field work? ☐ Yes ☒ No

Reporting for specific materials, systems and methods

We require information from authors about some types of materials, experimental systems and methods used in many studies. Here, indicate whether each material, system or method listed is relevant to your study. If you are not sure if a list item applies to your research, read the appropriate section before selecting a response.

Materials & experimental systems

n/a	Involved in the study
☒	☐ Antibodies
☒	☐ Eukaryotic cell lines
☒	☐ Palaeontology and archaeology
☒	☐ Animals and other organisms
☒	☐ Clinical data
☒	☐ Dual use research of concern

Methods

n/a	Involved in the study
☒	☐ ChIP-seq
☒	☐ Flow cytometry
☒	☐ MRI-based neuroimaging
